# Soy Formula Is Not Estrogenic and Does Not Result in Reproductive Toxicity in Male Piglets: Results from a Controlled Feeding Study

**DOI:** 10.3390/nu14051126

**Published:** 2022-03-07

**Authors:** Martin J. J. Ronis, Horacio Gomez-Acevedo, Kartik Shankar, Leah Hennings, Neha Sharma, Michael L. Blackburn, Isabelle Miousse, Harry Dawson, Celine Chen, Kelly E. Mercer, Thomas M. Badger

**Affiliations:** 1 Department of Pharmacology & Experimental Therapeutics, Louisiana State University Health Sciences Center, 1901 Perdido Str., New Orleans, LA 70112, USA; 2 Department of Biomedical Informatics, University of Arkansas for Medical Sciences, Little Rock, AR 72205, USA; gomezacevedohoracio@uams.edu; 3 Department of Pediatrics-Nutrition, University of Colorado Anschutz Medical Center, Aurora, CO 80045, USA; kartik.shankar@cuanschutz.edu; 4 Department of Pathology, University of Arkansas for Medical Sciences, Little Rock, AR 72205, USA; leah.hennings@buffaloriveranimalhealth.com; 5 Arkansas Children’s Nutrition Center, Little Rock, AR 72202, USA; shamaneha@uams.edu (N.S.); blackburnmichaell@uams.edu (M.L.B.); kelly.mercer@fda.hhs.gov (K.E.M.); badgerthomasm@uams.edu (T.M.B.); 6 Department of Biochemistry, University of Arkansas for Medical Sciences, Little Rock, AR 72205, USA; iracinemiousse@uams.edu; 7 USDA ARS Nutrition Center, Diet Genomics and Immunology Laboratory, Beltsville, MD 20705, USA; harry.dawson@usda.gov (H.D.); celine.chen@usda.gov (C.C.); 8 Department of Pediatrics, University of Arkansas for Medical Sciences, Little Rock, AR 72205, USA

**Keywords:** infant formula, soy, pig, testis, estradiol, genistein

## Abstract

Soy infant formula which is fed to over half a million infants per year contains isoflavones such as genistein, which have been shown to be estrogenic at high concentrations. The developing testis is sensitive to estrogens, raising concern that the use of soy formulas may result in male reproductive toxicity. In the current study, male White-Dutch Landrace piglets received either sow milk (Sow), or were provided milk formula (Milk), soy formula (Soy), milk formula supplemented with 17-beta-estradiol (2 mg/kg/d) (M + E2) or supplemented with genistein (84 mg/L of diet; (M + G) from postnatal day 2 until day 21. E2 treatment reduced testis weight (*p* < 0.05) as percentage of body weight, significantly suppressed serum androgen concentrations, increased tubule area, Germ cell and Sertoli cell numbers (*p* < 0.05) relative to those of Sow or Milk groups. Soy formula had no such effects relative to Sow or Milk groups. mRNAseq revealed 103 differentially expressed genes in the M + E2 group compared to the Milk group related to endocrine/metabolic disorders. However, little overlap was observed between the other treatment groups. These data suggest soy formula is not estrogenic in the male neonatal piglet and that soy formula does not significantly alter male reproductive development.

## 1. Introduction

Breastfeeding is the best choice for infant nutrition, and the American Academy of Pediatrics (AAP) suggests exclusive breastfeeding [1,2]. However, many women are unable to breastfeed and utilize infant formulas. AAP recommends feeding infant formulas for the first year. Of those infants fed formula, cow’s milk formula accounts for 69%, followed by soy formula at 12% [3]. Soy foods have been safely consumed in China and other Far Eastern countries for thousands of years. Moreover, soy infant formulas have been widely utilized for over 50 years, and many clinical studies report normal growth and development in infants consuming soy formula [4,5,6,7,8]. Nevertheless, some have suggested that soy formula use can result in adverse reproductive health outcomes out of concern for estrogen-like effects resulting from the presence of isoflavone polyphenols such as genistein bound to the soy protein [9,10,11,12]. As a result, in 2011, the National Toxicology Program raised its level of concern about the potential reproductive toxicity of soy formula from negligible to minimal [13]. Isoflavones are similar structurally to 17β estradiol (E2) [14,15]. Genistein has been shown to be estrogenic in certain species and/or at high concentrations in cell culture [5,14,16,17]. In addition, genistein and the other soy-associated isoflavones, daidzein and equol, have the capacity to bind weakly to estrogen receptors (ERs) α (ESRA) and β (ESRB), with a preference for ESRB [18,19,20]. These compounds are considered endocrine-disrupting compounds, which have the potential to interfere with reproductive development and fertility [21].

Testicular Sertoli, Leydig, and germ cells express ESRA and ESRB [18,22,23], and exposure to estrogens during early male development is well-known to interfere with growth and development of the testis, suppress androgen production, and reduce fertility [24]. With respect to soy isoflavones, Wisniewski et al. [25] reported that dietary genistein fed through gestation and lactation reduces testis size and testosterone concentrations in rats and disrupts reproductive behavior in adulthood. More recently, Meena et al. [26] have reported prenatal, i.p., genistein exposure-reduced fertility in adult male rats associated with a deterioration of testicular architecture, dose-dependent reductions in sperm production, and decreased plasma testosterone. In addition, Sharpe and co-workers [11,12] reported reduced serum testosterone and increased Leydig cell numbers in marmoset monkeys following postnatal feeding of soy infant formula, although no lasting effects were observed at adulthood.

In contrast, we have reported no effects on development of male reproductive organs or effects on fertility in rats fed soy protein isolate (SPI), the sole protein source in soy infant formula, throughout development [5,27]. Moreover, we have observed different gene expression profiles in many estrogen-responsive organs of adult and prepubertal rats and in the mammary glands of female neonatal piglets after feeding SPI and soy infant formula relative to those elicited by E2 [28,29,30,31,32,33,34]. These data suggest that the endocrine effects of soy products and E2 differ significantly.

The neonatal piglet is a good surrogate for many aspects of infant physiology and nutrition [34,35,36]. Piglets can be fed commercially available infant formulas. In the current study, we utilized this model to test the hypothesis that soy formula or supplementation of cow’s milk formula with genistein at levels found in soy formula, would activate ER-signaling pathways in the testis, resulting in feminization, and the results determined whether that hypothesis should be rejected. Therefore, groups of male piglets aged two days were fed either cow’s milk formula, cow’s milk formula supplemented with genistein at levels producing serum concentrations similar to those of soy formula-fed infants, soy formula, or sow milk via bowl feeding until postnatal day (PND) 21. To serve as positive control, an additional group was fed cow’s milk formula supplemented with E2 to activate ER signaling pathways for comparison purposes.

## 2. Materials and Methods

### 2.1. Animal Experiments

Piglets were housed at the Arkansas Children’s Hospital Research Institute (ACHRI). Animal maintenance and experimental treatments were conducted in accordance with the ethical guidelines for animal research established and approved by the Institutional Animal Care and Use Committee at the University of Arkansas for Medical Sciences. A detailed description of this protocol has been previously reported, and additional data from this study has been previously published [34]. In brief, litters containing 8–11 piglets from Dutch Landrace Duroc sows fed a soy-free diet were used. Piglets were selected at random from many different litters born on the pig farm on the same day to form the different diet groups and to avoid litter effects. At birth, male piglets were allowed to suckle at the farm for 2 days, then transferred to the animal facility at ACHRI. Piglets were then randomly assigned to one of 5 groups and fed commercially obtained sow milk by bowl feeding (Sow, *n* = 5), a cow’s milk-based formula (Milk, *n* = 6) (Similac Advance powder, Ross Products, Abbott Laboratories, Abbott Park, IL, USA), a soy-based formula (Soy, *n* = 6) (Enfamil Prosobee Lipil powder, Mead Johnson Nutritionals, River Point, Chicago, IL, USA), the same cow’s milk-based formula (Similac Advance, Ross Products, Abbott Laboratories, Abbott Park, IL, USA) supplemented with 2 mg/kg/d E2 (M + E2, *n* = 6) as a positive control, or the Similac Advance with pure genistein (84 mg/L diet) (M + G, *n* = 6) to produce serum levels comparable to those found in soy formula-fed piglets and infants for 21 days. Formula diets were modified to meet the needs of the growing pig. A detailed description of the nutrient composition of the sow milk and formula diets has previously been published [35]. Piglets were all housed individually and trained to drink from small bowls on a fixed schedule: the first week was every 2 h, the second week was every 4 h, and the third week was every 6 h, to provide 1.04 MJ/kg/d until day 21. In the M + E2 group, E2 was added to the food bowls at each feeding to achieve the serum E2 concentrations achieved in peripubertal piglets (15.6–30 pmol/L) [34]. In the M + G group, genistein was added to the food bowls to achieve a concentration (84 mg/L of diet) that was comparable to the genistein concentrations in SPI, 54 mg/L, (Dupont Nutrition and Biosciences, St. Louis, MO, USA). Piglets were euthanized after anesthetization, 6–8 h after the final feeding period. Blood was collected, and serum was stored at −20 °C until analysis for isoflavones and hormones. Testis, prostate, and numerous other tissues were weighed, and tissue samples were fixed in formalin or snap-frozen in liquid nitrogen and stored at −70 °C until analysis.

### 2.2. Serum Soy Isoflavones

Serum isoflavones were extracted and analyzed by LC-MS as previously published [34].

### 2.3. Serum Hormones

Follicle stimulating hormone (FSH) and luteinizing hormone (LH) and testosterone concentrations were measured using ELISA kits, NB-ES0109, BG-POR11481, and NB-ES104, respectively, from Novateinbio (Cambridge, MA, USA). Androstenedione and progesterone were measured using ELISA kits 07-109202 and 07-170102 from MP Biomedicals (Solon, OH, USA). Dihydrotestosterone (DHT) was measured using kit DSL-9600 from Diagnostic Systems Labs Inc. (Webester, TX, USA), and dehydroepiandrosterone (DHEA) and DHEA-sulfate (DHEA-S) concentrations were also measured by ELIA kits PN0980 and PN0953 from TSZ (Framingham, MA, USA). E2 concentrations were measured using a customized MULTI-SPOT^®^96-well Custom Steroid Hormone Panel Plate (Lot # Z0055407, Meso Scale Diagnostics, Rockville, MD, USA) using the MESO SQ120 QuickPlate instrument.

### 2.4. Testis Morphology and Cellularity

Bouin’s fixed testis were sectioned, paraffin-embedded, and stained with H&E. Testicular morphology (tubular volume/total volume) and cellularity (numbers of Leydig, Sertoli, and germ cells) were assessed in a blinded fashion in 3 fields from 4 different 1.5 × 1.5 cm sections per animal by a veterinary pathologist (L.H.) as described by Lanning et al. [36].

## 3. Gene Expression Analysis by mRNAseq

Total RNA was isolated from frozen testes through RNeasy-mini columns (Qiagen, Valencia, CA, USA), including on-column DNAse digestion. cDNA library construction from the mRNA was carried out using NEB-Next reagents (New England Biolabs, Ipswich, MA, USA) following the manufacturer’s instructions. Sequencing of the cDNA Libraries was performed with the Genome Analyzer IIX using the TruSeq v5 reagents (Illumina Inc., San Diego, CA, USA). For details of cDNA library preparation and sequencing procedures, please refer to Ronis et al. [37]. On average, approximately 10 million 36 bp single-end reads per sample were generated. The FASTQ files with raw data were submitted to the National Center for Biotechnology Information (NCBI) Sequence Read Archive database (SRA) under the accession numbers GSE168627.

Nucleotides below Q25 or reads containing more than two ambiguous nucleotides were removed before sequence alignments performed by the CLC Genomics Workbench version 20.01 (QIAGEN Bioinformatics, Redwood City, CA, USA). For gene expression calculation, reads were first mapped to a custom, manually curated nonredundant (NR) 13,547 gene library [38]. The sequences of these genes are in the Porcine Translational Research Database (PTR, http://tinyurl.com/hxxq.3ur, accessed on 10 January 2022). The remaining unmapped reads were mapped again to the Ensembl pig genome build 11.1 (WG) in search of expressed genes that are not covered by the PTR database.

Transcriptomes built from the mapping results were subjected to differential expression analysis. Group correlations estimated by the Pairwise method (JMP Genomics, version 9, SAS, Cary, NC, USA) showed that sample “Sow 2” had correlation values below 0.9. Therefore, it was excluded from further analysis. The statistical analyses were carried out with the exact tests from the Bioconductor package “edgeR” (version 3.30.0; run on RStudio, version 4.0.3, Boston, MA, USA) [39]. Genes were considered differentially expressed with the thresholds of a false discovery rate (FDR) ≤ 0.05 and an absolute fold change ≥ 2.0. Pathway analysis was carried out with DAVID (https://david.ncifcrf.gov/) (accessed on 10 January 2022) to explore KEGG pathways.

## 4. mRNAseq Confirmation by Real-Time RT-PCR

Real-time RT-PCR was used to confirm expression changes of some genes regulated by E2, feeding of soy vs. milk formula or regulated by genistein. For each sample, total RNA was reverse-transcribed into cDNA and subsequent real-time RT-PCR analysis was carried out using SYBR green and ABI 7500 sequence detection system (Applied Biosystems, Waltham, MA) as described previously [35]. Gene expression was analyzed for each piglet using the 2^−∆∆ct^ method relative to a housekeeping gene 18S gene amplification and expressed as fold change compared with the Sow group. Primer sequences for: Star F 5′-TGCTCAGCATTGACCTCAAGGGAT-3′, R 5′-TTTCGAAGGTGATTGGCAAACTCC-3′; Cyp17a1 F 5′-GGTGCCCAGACCACAATTTA-3′, R 5′-CTTTACCACAGAGGCAGAAGTC3′, and Cyp19 F 5′GAATTCATGAGGGTCTGGATAGG-3′, R 5′-CCAAATCGGCACGTGTAATG-3′.

## 5. Statistical Analysis

All experimental outcomes other than the data from mRNAseq analysis are summarized as mean ± SE. Differences in isoflavone concentrations between Soy and M + G groups were determined by 2-tailed Student’s *t*-test, *p* < 0.05. With respect to body weight, tissue weight, testis morphology, hormone concentrations and real-time RT-PCR gene expression, comparisons between sow and the formula diets and between MILK and M + E2 or M + G supplemented groups were accomplished by one-way ANOVA followed by a Student-Newman-Keuls post hoc analysis *p* < 0.05 except in cases of non-normal distribution where statistical differences between groups was verified by one-way ANOVA of Ranks followed by Dunn’s post hoc analysis.

## 6. Results

### 6.1. Serum Soy Isoflavone Concentrations Detected in the Soy- and M + G-Fed Piglets

Serum isoflavone concentrations in male piglets from this study were determined by LC-MS as previously described [34]. In the Soy-fed group we detected, genistein, daidzein and small amounts of glycitein, and o-desmethylangolansin (DMA) in the serum comparable to circulating soy isoflavones, reported in infants aged 4 months consuming soy formula [15]. Genistein values were 1712 ± 212 pg/mL and daidzein values were 1673 ± 374 pg/mL (Table 1). As observed in human neonates fed soy formula, levels of serum equol (conjugated or unconjugated) were undetectable in all diet groups nor was there any evidence for the presence of the equol precursor dihydrodaidzein. We did detect genistein in the M + G group (1898 ± 515 pg/mL). Genistein concentrations between Soy and M + G groups did not differ significantly.

### 6.2. Effects of Formula Feeding, Genistein and E2 on Body and Organ Weights

Differences in body weight and testis and prostate weights in the different groups of male piglets on PND 21 at the end of feeding are shown in Table 2. Direct comparisons were made of the effects of diet between sow-fed and formula-fed piglets and between milk formula and the effects of supplementation with pure genistein or E2. No significant differences were observed in body weight. Consumption of soy formula resulted in a small increase in absolute testis weight relative to those in the Sow and Milk groups (*p* < 0.05). However, significance was lost when corrected for body weight. E2 addition to cow’s milk formula significantly reduced testis size in both absolute terms and when corrected for body weight compared to all other groups (*p* < 0.05). In contrast, neither diet nor addition of genistein or E2 affected piglet prostate weight.

### 6.3. Effects of Formula, Genistein, and E2 on Endocrine Parameters in Neonatal Male Piglets

Differences between treatment groups in serum gonadotropins and androgen concentrations are shown in Table 3. Effects on additional gonadal and adrenal steroids are shown in Table 4. Both feeding soy formula and addition of genistein to milk formula resulted in significant increases in serum FSH concentrations relative to soy milk- or cow’s milk-fed groups (*p* < 0.05). In contrast, addition of E2 to milk formula had the opposite effect and the Milk + E2 group had lower serum FSH than the Milk + Genistein group (*p* < 0.05). (Table 4).

No significant effects of formula feeding, genistein, or E2 were observed on serum LH concentrations, which were low and quite variable. The testosterone precursor androstenedione was slightly lower in cow’s milk formula-fed piglets compared to that in sow milk- or soy milk-fed animals (*p* < 0.05), and this was reduced further by addition of E2. Likewise, serum testosterone concentrations were significantly suppressed by E2 treatment relative to all other treatment groups (*p* < 0.05). No significant effects of formula feeding, genistein, or E2 treatment were observed on serum concentrations of DHT (Table 3). As far as effects of diet on other steroids are concerned, feeding either formula reduced serum progesterone concentrations relative to that when feeding with sow milk (*p* < 0.05) (Table 4). No significant effects of diet were observed on serum concentrations of E2 or estrone or the adrenal steroid DHEA (Table 4). However, soy formula did slightly elevate circulating concentrations of the major prepubertal steroid hormone DHEA sulfate (DHEA-S) (*p* < 0.05) (Table 4). Addition of genistein to cow’s milk formula had no significant effects on any of these steroids. However, as expected, addition of E2 to milk formula resulted in substantial increases in circulating E2 and its metabolite estrone (Table 4).

### 6.4. Effects of Formula Feeding, Genistein, and E2 on Testicular Morphology and Cellularity in Neonatal Male Piglets

**Figure 1:**
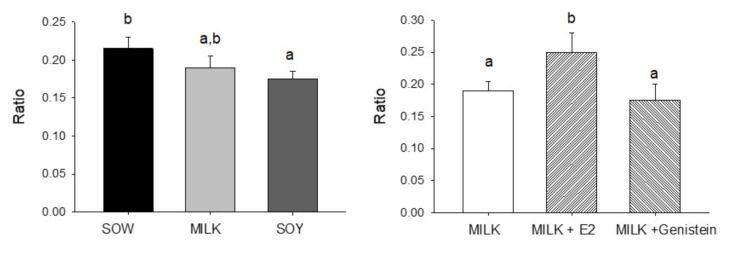
Effects of infant formula or estradiol (E2) and genistein on piglet testicular tubule area/total area. Data are mean ± SEM based on assessment of 4 fields from 3 sections of each animal (*n* = 6/group). a < b statistically different.

Effects of neonatal diet on testis morphology are shown in Figure 1. Although tubule area/total area was reduced (*p* < 0.05) in soy formula-fed piglets relative to those of the sow milk-fed group, this parameter did not differ significantly between the two formula-fed groups. In contrast, addition of E2 to milk formula resulted in a significant increase in tubule area/total area relative to those of the cow’s milk formula group (*p* < 0.05). Addition of genistein resulted in no significant effect relative to feeding cow’s milk by itself (Figure 1). No significant effects of either formula or addition of E2 or genistein to cow’s milk formula were observed.

**Figure 2:**
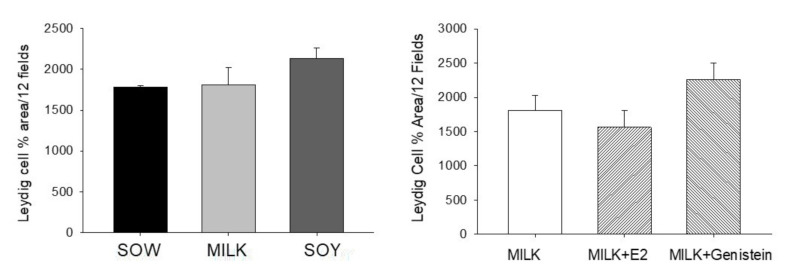
Effects of infant formula or estradiol (E2) and genistein on piglet testicular Leydig cell numbers. Data are mean ± SEM based on assessment of 4 fields from 3 sections of each animal (*n* = 6/group). There were no significant differences between groups.

**Figure 3:**
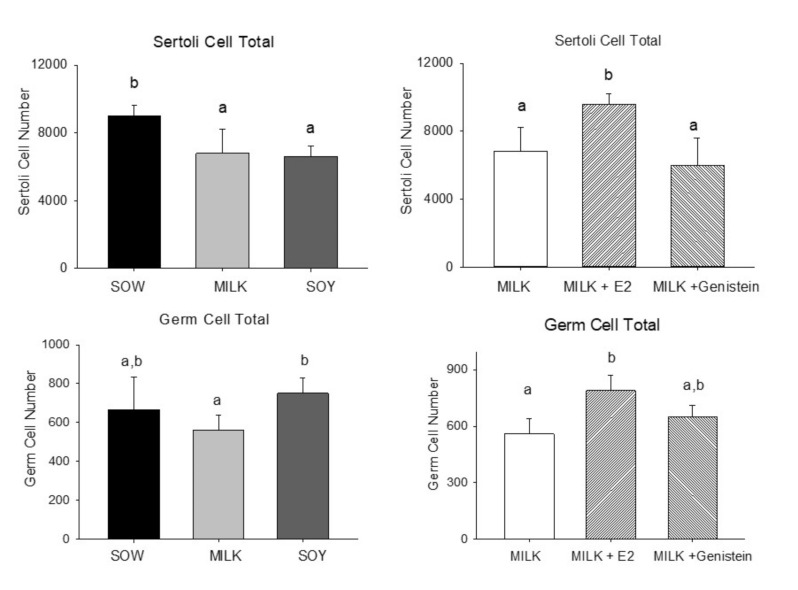
Effects of infant formula or estradiol (E2) and genistein on piglet testicular Sertoli cell and germ cell numbers. Data are mean ± SEM based on assessment of 4 fields from 3 sections of each animal (*n* = 6/group). a < b statistically different.

Effects on testicular Leydig cell number are shown in Figure 2. Formula feeding from either cow’s milk or soy both resulted in reduced Sertoli cell numbers relative to feeding sow milk (*p* < 0.05) but soy formula and cow’s milk formula groups did not differ significantly (Figure 3).

In contrast, E2 treatment increased Sertoli cell number in the M + E2 group vs. the Milk group (*p* < 0.05), while addition of genistein had no effect (Figure 3). Consumption of cow’s milk formula reduced germ cell numbers relative to those with soy formula feeding (*p* < 0.05) and feeding of sow milk, while sow and soy formula groups did not differ significantly. In contrast, germ cell numbers were increased by addition of E2 (*p* < 0.05) to cow’s milk formula relative to those of the Milk group (Figure 3).

### 6.5. Effects of Formula Feeding, Genistein, and E2 on Expression of mRNAs Encoding Steroid Biosynthetic Enzymes in Neonatal Male Piglet Testis

**Figure 4:**
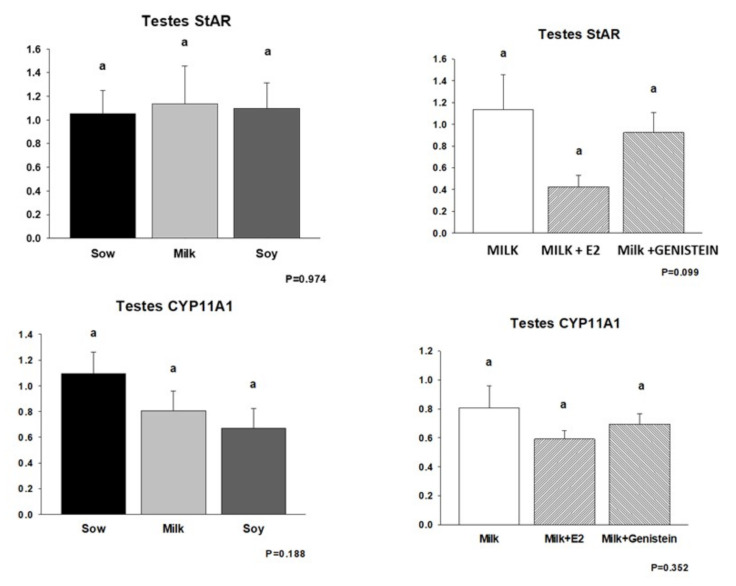
Effects of infant formula or estradiol (E2) and genistein on piglet testicular steroidogenic enzyme mRNA expression I: steroid-activated receptor (StAR) and cytochrome P450 CYP11A1. Data are mRNA/18S relative to sow milk (Sow) (formulas) or relative to cow’s milk formula (MILK) (E2/genistein) (*n* = 6/group). One-way ANOVA followed by Student–Newman–Keuls post hoc analysis only showed a trend (*p* = 0.99) for reduction of StAR mRNA expression by MILK + E2 relative to those of MILK or MILK + genistein. Means with the same superscript letter a do not differ statistically.

**Figure 5:**
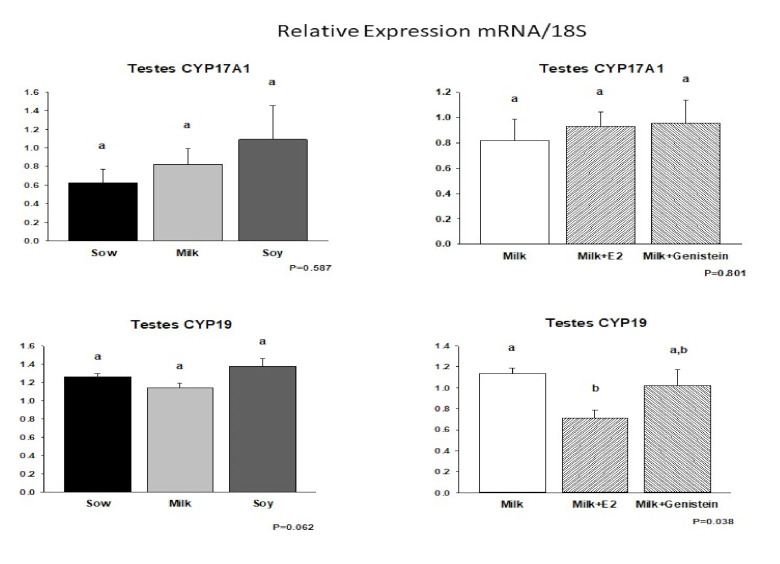
Effects of infant formula or estradiol (E2) and genistein on piglet testicular steroidogenic enzyme mRNA expression II: cytochrome P450 17A1 (CYP17A1) and cytochrome P450 19 (aromatase, CYP19). Data are mRNA/18S relative to sow milk (Sow) (formulas) or relative to cow’s milk formula (MILK) (E2/genistein) (*n* = 6/group). One-way ANOVA followed by Student–Newman–Keuls post hoc analysis only showed a trend (*p* = 0.062) for increased CYP19 mRNA expression by soy formula relative to that of sow milk or cow’s milk but b < a, *p* < 0.05 for E2 in cow’s milk relative to that in cow’s milk.

Given the suppression of piglet testicular growth and circulating androgenic steroids following E2 supplementation of cow’s milk formula, we examined the expression of testicular steroidogenic enzymes by real-time RT-PCR. These data are shown in Figure 4 and Figure 5. mRNA encoding the steroidogenic acute regulatory protein (StAR), which regulates cholesterol transport within the mitochondria and which is considered a rate-limiting step in steroid synthesis, was suppressed in the M + E2 group relative to that in the Milk and M + G groups (*p* = 0.099), but its expression was completely unaffected by either formula diet relative to that of piglets fed sow milk (Figure 4).

In contrast, no significant effects of neonatal diet, E2, or genistein supplementation were observed on expression of pregnenolone synthase (CYP11A1) (Figure 4) or steroid 17α-hydroxylase/17–20 lyase (CYP17A1) (Figure 5). In contrast, E2 supplementation of cow’s milk formula resulted in suppression of expression of aromatase (CYP19) mRNA (*p* < 0.05), which encodes the enzyme converting androgens to estrogens. No effects of neonatal diet or genistein supplementation were observed on CYP19 mRNA expression (Figure 5).

### 6.6. RNAseq Analysis of Male Piglet Testis

**Figure 6:**
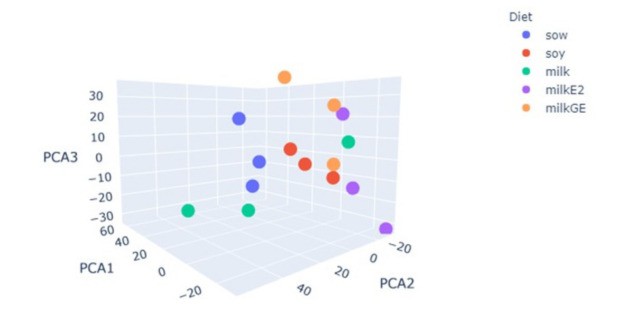
Principal component analysis(PCA) of pig testicular mRNAseq data demonstrating lack of clustering between soy formula-, genistein-, or E2-treated groups.

**Figure 7:**
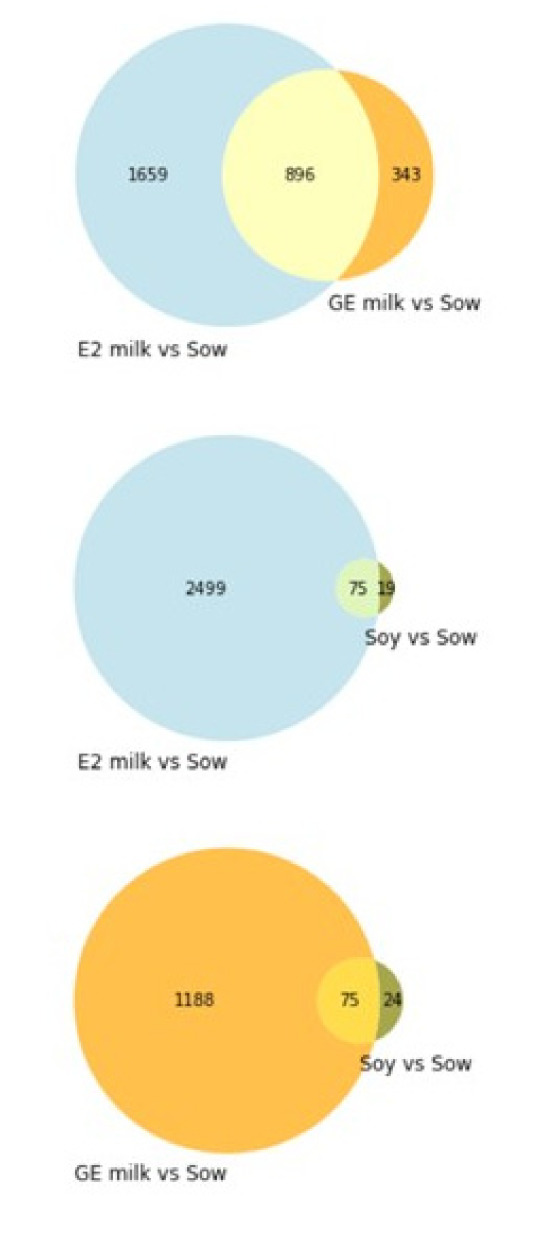
Pie charts illustrating overlap in gene expression profiles between E2 and genistein groups; E2 and soy formula groups and genistein and soy formula groups.

RNAseq analysis was conducted on RNA extracted from the testes of all experimental groups. Principal component analysis on the log scale for RPKM values revealed that all the experimental groups were different. Cow’s milk and soy formula groups did not segregate together but were both very different from the sow milk group. Addition of either E2 or genistein to cow’s milk resulted in different RNA expression relative to that of the Sow, but neither pattern was similar to that of the soy formula group (Figure 6). Primary direct comparisons were made between the Sow group and other treatments. A comparison between groups of genes regulated by more than two-fold showed some overlap between E2 and genistein groups relative to the Sow group, but very little overlap between the E2 and soy formula group (Figure 7).

Only 75 of 2555 regulated genes relative to Sow (2.9%) were in common between the E2-treated and soy-fed groups. There were 896 of 2555 regulated genes (35%) in common between E2- and genistein-supplemented groups. Seventy-five of 94 soy formula-regulated genes relative to Sow (80%) were also regulated by genistein, but these only represented 6% of genistein-regulated genes. The top KEGG pathways for the E2-supplemented group relative to the sow group were oxidative phosphorylation and ribosome, but other pathways such as metabolism of xenobiotics by cytochrome P450 and steroid biosynthesis were also affected (*p*-value < 0.1) (Table 5). Excel files reporting up- and downregulated testicular genes by E2, GE, and soy vs. sow formula-fed are found in Tables S1–S3, and all the RNAseq data are publicly available via the NCBI SRA database (GSE 1688627). The most upregulated gene in E2 was mysterin (RNF213), which contains an AAA domain associated with ATPase activity and has been recently reported as a regulator of cytoplasmic lipid droplets [40]. Another highly induced mRNA by E2 was the transcription factor FOXL2, which has been shown to be important in female sex determination [41].

E2 downregulated genes included the immune regulators GZMA and LECT2 [42,43,44,45]. The most upregulated gene by soy was SMAD6, which is an inhibitor of BMPs and TGF-b signaling, and in vitro experiments showed that SMAD6 overexpression blocks BMPs-induced osteoblast and chondrocyte differentiation [46]. Of note, men with deletions in the Y-chromosome DAZ cluster experience impaired spermatogenesis [47], and only in the M + G group did we find downregulation of the DAZ homolog (DAZL) [48].

**Figure 8:**
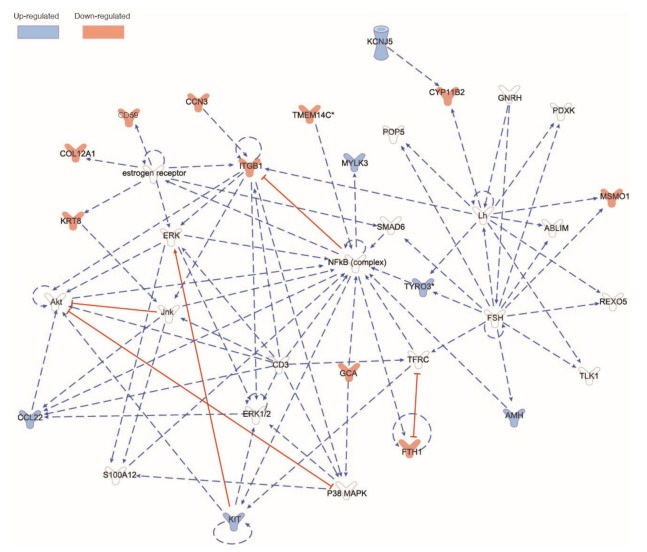
Ingenuity Pathways Assist analysis of M + E2 effects on gene expression and biochemical pathways relative to those of the Milk group. Estrogen induced and inhibited testicular gene expression. Gene names abbreviated according to the NCBI database (GeneCards.org).

Secondary direct comparisons were made between gene expression profiles in the M + E2, M + G, and soy formula groups vs. the Milk formula-fed group. We identified 103 genes differentially expressed in the M + E2 group vs. Milk. Three of these genes were pig-specific, namely DMBT1L, RPL21Ps, and UNK37, and other novel genes classified as long noncoding RNA ENSSSCG00000003503, ENSSSCG00000046393, and ENSSSCG00000049464. We conducted a pathway analysis with IPA (Figure 8). The networks related to endocrine system disorders and metabolic disorders had the larger number of members related directly or indirectly to our set. Figure 8 depicts the network with colored genes indicating the directionality of the comparison M + E2 vs. Milk. In contrast, there were only three genes highly upregulated in M + G vs. Milk, namely Defensin Beta 115 (DEFB115), Epithelial cell adhesion molecule (EPCAM), and Keratin 19 (KRT19). In rats, defensins are highly expressed in testis [49], suggesting that genistein may potentiate their expression. On the other hand, according to the Human Phenotype Oncology, EPCAM gene has been related to abnormalities of the genital system (HP: 000078). Finally, Keratin 19 gene (also known as CK19) has been shown as a biomarker for a certain type of testis tumors [50]. The comparison of Milk + E2 vs. soy formula groups revealed only four differentially regulated genes C-X-C motif chemokine ligand 13 (CXCL13), the long coding RNA ENSSSCG00000046140, Galactosidase beta 1 like 2 (GLIB1L2), and the mitochondrial gene ENSSSCG00000018062. None of these overlapped with the effects of E2 or genistein supplementation. The former two genes were downregulated by soy formula, whereas the last two were upregulated with respect to milk formula. Interestingly, it has been reported that blocking CXCL13 expression prevents the emergence of aggressive and metastatic prostate cancers treated with hormone ablation therapies [51].

## 7. Discussion

There is considerable discordance in the toxicological literature with regards to the potential estrogenicity and reproductive toxicity of soy infant formula as the result of the presence of the isoflavone phytoestrogens genistein and daidzein. This is especially true of effects on male reproductive development. Neonatal estrogens are well-known to reduce testis size, suppress steroidogenesis, and decrease testosterone concentrations [21,22,23,24]. Similar effects have been observed in rodents following perinatal treatment with genistein- or soy-containing diets [21,25,26]. Moreover, Sharpe et al. have reported decreased serum testosterone and increased Leydig cell numbers in marmoset monkeys after postnatal feeding of soy formula, suggesting development of compensated Leydig cell failure [11,12]. In contrast, studies in our laboratory have observed no male reproductive toxicity following lifetime feeding of soy protein isolate (SPI), the sole protein source in soy formula, and no pattern of estrogen-regulated gene in prenatal rats fed SPI in direct comparison to the effects of E2 [27,30,34]. Some of these differences may be related to the different effects of soy foods, which are complex mixtures of proteins and over 100 phytochemicals in addition to isoflavones, to differences in developmental window and route of administration, and to species’ differences in isoflavone metabolism [52,53]. Rodents and marmoset monkeys metabolize daidzein to the more potent estrogenic metabolite equol, whereas human infants do not make equol until after weaning [53,54]. The current study utilized the piglet, a more appropriate animal model for male neonatal development, which does not make equol [53], and directly compared the effects of soy and cow’s milk formula feeding with that of sow milk and the effects of cow’s milk formula supplementation with pure genistein levels comparable with those in soy formula or with E2 as a positive control.

E2 supplementation of cow’s milk formula resulted in the expected decrease in testis weight, steroidogenesis, and serum testosterone. In addition, E2 resulted in morphological changes in the neonatal pig testis, including increased tubular area, Sertoli and germ cell numbers, and significantly reduced serum FSH concentrations, and produced expected changes in testis gene expression profiles linked to endocrine/metabolic disorders. In contrast, neither soy formula nor pure genistein supplementation at levels comparable to those seen in soy formula had similar effects. In fact, soy formula significantly decreased tubular area and Sertoli cell numbers relative to those in the Sow group, and soy formula and genistein significantly increased FSH.

It is unclear if the effect on neonatal FSH has physiological significance, but this effect is clearly not associated with the activation of estrogenic signaling. In fact, these effects on testicular cellularity and FSH suggest possible anti-estrogenic actions as a result of competition between low-affinity isoflavones and high-affinity endogenous estrogens at the level of the estrogen receptors or estrogen-independent actions. In this regard, anti-estrogenic effects of SPI have previously been reported in the rat mammary gland [32]. The only similarity between SPI and E2 effects resided in a small increase in germ cell numbers in soy formula-fed vs. cow’s milk formula-fed groups which did not differ significantly from the germ cell numbers observed in sow milk-fed piglets. These results also differ significantly from those described by Sharpe et al. in marmoset monkeys fed soy formula [11,12] with no effects on testis size, serum testosterone, or Leydig cell numbers and no effect on serum LH concentrations. These effects may reflect species’ differences in isoflavone metabolism given the extensive production of equol in monkeys and lack of equol in piglets and human infants prior to weaning [53]. A more detailed characterization of testicular effects of diet, genistein, and E2 using RNAseq revealed clear E2 effects on multiple testicular pathways in the M + E2 group vs. the Milk group, but little overlap between E2 and soy formula-fed groups or between the genistein-supplemented and soy formula-fed groups. These data are consistent with previous studies on genome-wide comparisons of the effects of estradiol and soy protein isolate/soy formula in estrogen-responsive tissues of rat and piglets of different sexes and ages indicating that soy acts as a selective estrogen receptor modulator (SERM) rather than a complete estrogen, only overlapping with a small subset of estrogen-responsive pathways [28,29,30,31,32,33,34]. The strengths of this study are that it compared the effects of physiologically relevant levels of estrogens with that of soy formula in an animal model where commercial infant formula could be fed orally during the developmental window, corresponding to formula feeding in infants and where the pattern of isoflavone metabolites was the same as that found in infants. The limitations are that the number of animals per group was small, that extrapolation between animal models and humans must always be treated with caution, and that it is very difficult to definitively prove a negative.

In this regard, it is important to note that our data are also consistent with those of two recent human longitudinal clinical studies of breast-fed and formula-fed male infants. Clinical studies of soy formula-fed infants utilizing ultrasound analysis of breast bud, testis, and prostate development at ages of one and five years in the “Beginnings Study” at the Arkansas Children’s Nutrition Center found no significant effects of soy formula vs. cow’s milk formula in male infants [6,7]. Likewise, a more recent study at the NIEHS also found no significant effects of soy formula vs. cow’s milk formula on breast bud development or urethral epithelial maturation index or estrogen or FSH hormone trajectories in soy formula-fed boys during the first seven months of life [10]. These clinical studies differed in results relating to formula-fed girls. In the “Beginnings Study”, no significant effects of soy formula feeding were observed on breast bud development and uterine or ovarian development using ultrasound analysis of girls fed soy vs. cow’s milk formula at ages of one and five years [6,7]. In contrast, the NIEHS study observed a significantly higher vaginal cell mitotic index and slower decreases in uterine volume in soy formula-fed vs. those in cow’s formula-fed girls [10]. However, these investigators also observed no effects of soy on breast bud dimeter or hormone concentrations in infant girls. It is unclear why the results of these clinical studies differ. In particular, it is unclear why, if soy indeed acts as an estrogen, the latter study did not also observe significant effects on female breast bud development or on male parameters, both of which have been shown to be highly sensitive to estrogens. An additional epigenetic analysis of the vaginal cells from the formula-fed girls in the NIEHS study has been conducted [9]. This study observed changes in methylation of CpG islands near the transcription start site of one estrogen-regulated gene proline rich 5-like (PRR5L) in cells from soy formula-fed girls vs. that of cow’s milk formula group. Such a result is not inconsistent with the hypothesis that soy acts as a SERM interacting with a small subset of estrogen-regulated genes.

## 8. Conclusions

These data strongly suggest that neither soy formula nor genistein were estrogenic in the neonatal porcine testis and that soy formula feeding during neonatal development did not significantly affect male reproductive development in this model. Moreover, these data are consistent with the conclusions of three recent comprehensive reviews of the clinical literature on the effects of dietary isoflavones and soy products [17,55,56] that there is little to no evidence that genistein or other phytoestrogens in soy formula consumed during the postnatal period significantly alter male reproductive development. Collectively, these studies are in agreement with the support of experts in the U.S. for the use of soy formula as a safe and cost-effective alternative to cow’s milk formula [55].

## Figures and Tables

**Table 1:** Serum isoflavone concentrations in neonatal piglets fed sow milk or formula.

Diet	Genistein (pg/mL)	Daidzein (pg/mL)	Equal (pg/mL)	0-DMA (pg/mL)	Glycitein (pg/mL)
Sow Milk	56 ± 8 ^a^	256 ± 7 ^a^	5 ± 1	0 ^a^	139 ± 87 ^a^
Milk Formula	23 ± 9 ^a^	2 ± 1 ^a^	4 ± 1	0 ^a^	0 ^a^
Soy Formula	1712 ± 212 ^b^	1673 ± 374 ^b^	3 ± 1	2995 ± 714 ^b^	4567 ± 867 ^b^
Milk + Genistein	1898 ± 515 ^b^	7 ± 1 ^a^	9 ± 3	0 ^a^	379 ± 241 ^a^
Milk + E2	142 ± 1 ^a^	3 ± 2 ^a^	5 ± 1	2 ± 1 ^a^	9 ± 1 ^a^

Data are mean ± SEM (*n* = 6). Means with different letters are significantly different *p* < 0.05, ^a^ < ^b^ by one-way ANOVA followed by Student–Newman–Keuls post hoc analysis.

**Table 2:** Body and sex organ weights of neonatal pigs fed sow milk or formula.

	Body Weight	Testis Weight	%BW	Prostate Weight	%BW
	(kg)	(g)		(g)	
Sow Milk	7.30 ± 0.18	6.64 ± 0.76 ^a^	0.0090 ± 0.0009	0.38 ± 0.02	0.0005 ± 0.0001
Milk Formula	8.33 ± 0.27	7.93 ± 0.57 ^a,^*	0.0096 ± 0.0010 *	0.37 ± 0.03	0.0005 ± 0.0001
Soy Formula	8.52 ± 0.38	10.54 ± 1.37 ^b^	0.0122 ± 0.0013	0.31 ± 0.04	0.0004 ± 0.0001
Milk + Genistein	7.88 ± 0.42	9.46 ± 1.50 ^a,^*	0.0122 ± 0.0014 *	0.36 ± 0.05	0.0005 ± 0.0001
Milk + E2	8.27 ± 0.47	4.31 ± 0.5 ^a,#^	0.0052 ± 0.0006 ^#^	0.44 ± 0.05	0.0005 ± 0.0001

Data are mean ± SEM (*n* = 6). Means with different letters are significantly different from that of the sow milk group *p* < 0.05, ^a^ < ^b^; means with different symbols are significantly different from that of the milk formula group *p* < 0.05, ^#^ < * by one-way ANOVA followed by Student–Newman–Keuls post hoc analysis.

**Table 3:** Gonadotropin–testis axis hormones in serum of male piglets fed sow milk or formula.

Diet	FSH (mIU/mL)	LH (mIU/mL)	Androsterone (ng/mL)	Testosterone (ng/mL)	DHT (ng/mL)
Sow Milk	0.10 ± 0.05 ^a^	0.007 ± 0.007	76 ± 8 ^b^	0.52 ± 0.10 ^b^	1.65 ± 0.17
Milk Formula	0.13 ± 0.04 ^a,#^	0.047 ± 0.02	42 ± 6 ^a,#^	0.35 ± 0.02 ^b,*^	1.33 ± 0.17
Soy Formula	0.52 ± 0.04 ^b^	0.001 ± 0.001	86 ± 5 ^b^	0.50 ± 0.10 ^b^	1.46 ± 0.14
Milk + Genistein	0.34 ± 0.04 ^b,^*	0.052 ± 0.003	64 ± 8 ^b,^*	0.42 ± 0.08 ^b,^*	1.24 ± 0.20
Milk + E2	0.07 ± 0.04 ^a,#^	0.040 ± 0.03	28 ± 6 ^a,#^	0.09 ± 0.02 ^a,#^	0.88 ± 0.26

ANOVA results for group comparison and post hoc analysis. Data are mean ± SEM (*n* = 6), and means with differing letters differ significantly from that of the sow milk group *p* < 0.05, ^a^ < ^b^; means with different symbols within the milk groups differ from those of the milk formula group *p* < 0.05, ^#^ < *.

**Table 4:** Serum levels of non-androgenic steroid hormones in male piglets fed sow milk or formula.

Diet	DHEA (ng/mL)	DHEA-S (ng/mL)	Progesterone (ng/mL)	Estradiol (pg/mL)	Estrone (pg/mL)
Sow Milk	3.9 ± 0.2	29.7 ± 1.1 ^a^	4.20 ± 0.25 ^b^	115 ± 22 ^a^	354 ± 42 ^a^
Milk Formula	4.5 ± 0.2	30.5 ± 1.6 ^a^	0.59 ± 0.05 ^a^	78 ± 18 ^a,#^	240 ± 23 ^a,#^
Soy Formula	4.2 ± 0.2	36.7 ± 1.2 ^b^	1.12 ± 0.23 ^a^	113 ± 12 ^a^	335 ± 31 ^a^
Milk + Genistein	4.2 ± 0.4	32.1 ± 1.0 ^a,b^	0.54 ± 0.26 ^a^	108 ± 12 ^a^	284 ± 21 ^a,#^
Milk + E2	4.5 ± 0.4	32.7 ± 2.2 ^a,b^	0.85 ± 0.21 ^a^	1376 ± 418 ^b,^*	1615 ± 321 ^b,^*

Data are mean ± SEM (*n* = 6). Means with differing letters are significantly different from the sow milk group *p* < 0.05, ^a^ < ^b^. Means with different symbols are significantly different from the milk formula group within milk-fed groups *p* < 0.05, ^#^ < * by one-way ANOVA followed by Student–Newman–Keuls post hoc analysis.

**Table 5:** Top KEGG pathways for Milk + E2 vs. Sow groups.

PATHWAY	*p*-Value	FDR
ssc05204: Chemical carcinogenesis	0.027065737	0.481770112
ssc04146: Peroxisome	0.030864823	0.515056728
ssc00980: Metabolism of xenobiotics by cytochrome P450	0.036679394	0.576082249
ssc01212: Fatty acid metabolism	0.051689275	0.766724247
ssc00100: Steroid biosynthesis	0.071473127	0.970909091
ssc05205: Proteoglycans in cancer	0.092373727	0.970909091
ssc03018: RNA degradation	0.097239374	0.970909091
ssc04260: Cardiac muscle contraction	0.097239374	0.970909091
ssc00650: Butanoate metabolism	0.0987639250	0.970909091

FDR: false discovery rate.

## Data Availability

All the RNAseq data are publicly available via the NCBI SRA database (GSE 1688627).

## Supplementary Materials

The following supporting information can be downloaded at: www.mdpi.com/article/10.3390/nu14051126/s1, Tables S1–S3: Up- and downregulated testicular genes by M + E2, M + G and soy vs. sow formula-fed.
