# Supplementary material for: Soy Formula Is Not Estrogenic and Does Not Result in Reproductive Toxicity in Male Piglets: Results from a Controlled Feeding Study
Source: Nutrients. 2022 Mar 7;14(5):1126. doi: 10.3390/nu14051126 (PMC8912539; doi:10.3390/nu14051126)
Supplement: Supplementary file 1 [file nutrients-14-01126-s001.zip › Supplemental table 1.pdf]

Reannotated Gene	Gene	Fold Change	FDR
RNF213	RNF213_tvX1	83.9	0
FOXL2	FOXL2	60.9	0
MUC6	ENSSSCG00000012835	47.4	0.01
ENSSSCG00000042543	ENSSSCG00000042543	46.3	0
LOC102166616	ENSSSCG00000049504	44.1	0.01
ADTRP	ADTRP	41	0.01
KIF25	KIF25_tv1	37.6	0.01
PDE4A	PDE4A_tv2	34.9	0.02
PTPRH	PTPRH	33.5	0.03
CBLN4	CBLN4	30.5	0.05
GPR143	ENSSSCG00000012103	19.8	0
RRP8	RRP8	13.7	0
CYP1A1	CYP1A1	13.1	0
NOTCH3	NOTCH3_tv1	13	0.01
SYNDIG1L	SYNDIG1L	11.7	0.02
ZNF521	ZNF521_tv1	11.6	0.01
OBSCN	OBSCN_tv1	11.4	0.01
DMBT1L	ENSSSCG00000046807	11.3	0
PGLYRP1	PGLYRP1	11	0
HYOU1	HYOU1_tv2	10	0.02
MRGPRX2L1*	MRGPRX2L1*	9.9	0.01
LINGO1	LINGO1	9.8	0
ENSSSCG00000048148	ENSSSCG00000048148	9.5	0
CIC	CIC_tvX1	9.4	0
UNK129*	UNK129	9.4	0
PRR7	PRR7	9.1	0.01
ENSSSCG00000050327	ENSSSCG00000050327	9	0.01
ARHGEF1	ARHGEF1_tv2	8.9	0.02
NUP214	NUP214_tv2	8.9	0.03
MUC5B	ENSSSCG00000033452	8.5	0.02
PRSS3L1	PRSS3L1	8.1	0.03
SIX2	SIX2	8.1	0
IL17REL	ENSSSCG00000000983	8	0.04
HGSNATPs*	ENSSSCG00000041416	7.7	0.04
ENSSSCG00000041828	ENSSSCG00000041828	7.6	0.04
ENSSSCG00000045623	ENSSSCG00000045623	7.6	0.04
CX3CL1	CX3CL1_tv1	7.4	0.05
SIAH3	SIAH3	7.1	0
LMX1B	LMX1B	7.1	0
TRPC7	TRPC7	6.7	0
BRI3BPPs*	ENSSSCG00000041154	6.7	0
SOX8	SOX8	6.5	0.02
C1orf167	ENSSSCG00000046656	6.4	0
ARHGAP32	ARHGAP32_tv2	6.4	0.01
NUMA1	NUMA1_tvX1	6.3	0
NDUFA4L2	NDUFA4L2	6.3	0.01

GATA1	GATA1	6.3	0.02
ENSSSCG00000042711	ENSSSCG00000042711	6.3	0
MUC6	MUC6	6.3	0.01
LncRNA	LncRNA	6	0
MAP4K4	MAP4K4_tv1	5.8	0.02
ARHGAP45	ARHGAP45_tv1	5.8	0.02
TNXB	TNXB_tv3	5.8	0.01
GPAT2L*	ENSSSCG00000037588	5.8	0.02
NR2F1	NR2F1_tv1	5.7	0
KCNC1	KCNC1	5.6	0
ENSSSCG00000048770	ENSSSCG00000048770	5.6	0.02
JADE2	JADE2_tv2	5.5	0.01
COL9A1	COL9A1_tv1	5.4	0
R3HDM2	R3HDM2	5.4	0
AANAT	AANAT	5.3	0.04
FZD9	FZD9	5.3	0
PTGIR	PTGIR	5.3	0
RERE	RERE_tv2	5.2	0.04
FABP3L2	FABP3L2	5.2	0.01
PTPRS	PTPRS_tvX1	5.1	0.01
CACNA1B	CACNA1B_tv1	5	0.01
FCGBP	FCGBP	5	0.01
SELENOV	SELENOV_tv1	4.9	0.05
LncRNA	LncRNA	4.9	0
BCL11B	BCL11B	4.9	0.01
PCDHB10	PCDHB10	4.8	0.04
ENSSSCG00000045565	ENSSSCG00000045565	4.7	0.01
LIN28B	LIN28B	4.7	0.04
LAG3	LAG3	4.6	0.03
MKL2	MKL2_tv1	4.6	0.01
PCDH1	PCDH1_tv2	4.6	0
SLC8A2	SLC8A2	4.6	0.01
SNORA54	SNORA54	4.6	0.02
ENSSSCG00000051007	ENSSSCG00000051007	4.5	0
LOC105372912	ENSSSCG00000041374	4.5	0.02
EPPK1	EPPK1	4.5	0
LOC110260214	ENSSSCG00000045100	4.5	0.03
SLC35F6	SLC35F6	4.5	0.01
LOC100514340	ENSSSCG00000032383	4.4	0
ENSSSCG00000047888	ENSSSCG00000047888	4.4	0
HPCAL1	HPCAL1	4.4	0
CXCL12	CXCL12_tv3	4.4	0
ZNF446	ZNF446_tv1	4.3	0.05
ITPKB	ENSSSCG00000047967	4.3	0.04
CORO2A	CORO2A	4.3	0.01
LOC110255902/LOC110256040	ENSSSCG00000047490	4.3	0
SAXO1	SAXO1	4.3	0.04

ENSSSCG00000043864	ENSSSCG00000043864	4.3	0.02
KCNG1	KCNG1	4.2	0.02
ROM1	ROM1	4.2	0.02
TVP23A	TVP23A	4.2	0
SPINK5	SPINK5	4.2	0
ENSSSCG00000045307	ENSSSCG00000045307	4.1	0.03
PRODH	PRODH	4.1	0.01
TNRC6B	TNRC6B_tv3	4.1	0
FURIN	FURIN_tv2	4.1	0.05
COL18A1	COL18A1_tv2	4.1	0
ENSSSCG00000044418	ENSSSCG00000044418	4.1	0
NRIP2	NRIP2	4	0.01
SLC2A9	SLC2A9	4	0.02
AHSP	AHSP_tv1	4	0
LOC110260394	ENSSSCG00000042007	4	0
ENSSSCG00000044546	ENSSSCG00000044546	4	0.04
ENSSSCG00000042223	ENSSSCG00000042223	4	0.01
EDN3	EDN3	4	0.04
COLQ	COLQ	4	0.04
IGFN1	ENSSSCG00000036948	4	0
RGS12	RGS12_tv2	4	0
ERICH3	ENSSSCG00000003781	4	0
FOXH1	FOXH1	3.9	0.03
ISM1	ISM1	3.9	0.02
KIAA1109	KIAA1109_tv2	3.9	0
DCHS1	DCHS1_tv1	3.9	0
CD300CL*	CD300CL*	3.9	0
ENSSSCG00000041762	ENSSSCG00000041762	3.9	0.02
FER1L4	FER1L4	3.9	0.04
CR1	CR1	3.9	0
THSD4	THSD4_tvX1	3.8	0
MOG	MOG_tva	3.8	0
SNORA48	SNORA48	3.8	0.01
SMIM31	SMIM31	3.8	0.05
ETS1	ETS1_tv1	3.8	0
PPP1R3G	ENSSSCG00000041566	3.8	0.02
ENSSSCG00000035996	ENSSSCG00000035996	3.8	0.05
EVPL	EVPL	3.8	0
IGHG3	IGHG3	3.8	0.04
TMEM37	TMEM37	3.8	0
PAX5	PAX5_tv1	3.8	0.02
NPY6R	NPY6R	3.8	0.01
PRSS45P	PRSS45P	3.8	0
KCNH1	KCNH1	3.8	0
ENSSSCG00000042133	ENSSSCG00000042133	3.7	0.01
SLFN11	ENSSSCG00000032652	3.7	0.03
FBXL13	FBXL13	3.7	0

ALOX12	ALOX12	3.7	0
SDC3	SDC3	3.7	0
SPACA6	SPACA6	3.7	0
C17orf82	C17orf82	3.7	0.05
QRFPR	QRFPR	3.6	0
KCNMB1	KCNMB1_tv1	3.6	0
LOC110255544	ENSSSCG00000041966	3.6	0.02
SLC51B	SLC51B_tv	3.6	0.01
COL4A3	COL4A3	3.6	0.01
APLN	APLN	3.6	0
ENSSSCG00000000207	ENSSSCG00000000207	3.6	0.03
NEFM	NEFM	3.6	0.01
CEACAM16	CEACAM16	3.6	0.02
KCNQ1OT1	KCNQ1OT1	3.6	0.01
ENSSSCG00000048251	ENSSSCG00000048251	3.6	0.03
PLEC	PLEC	3.6	0
LncRNA	LncRNA	3.6	0
SOX10	SOX10	3.5	0.02
PLXNB3	PLXNB3	3.5	0
P4HA1	P4HA1_tv1	3.5	0
MYH9	MYH9	3.5	0
SLC13A5	SLC13A5_tv1	3.5	0
BMP6	BMP6	3.5	0
HAS1	HAS1	3.4	0
DNHD1	DNHD1_tv1	3.4	0.01
PCDHAC2	PCDHAC2_tv1	3.4	0.01
SLIT1	SLIT1_tv1	3.4	0
TAFA5	TAFA5	3.4	0
BSN	BSN_tv1	3.4	0
SHISA7	SHISA7	3.4	0.01
LOC110260790	ENSSSCG00000049984	3.4	0
MXRA5	ENSSSCG00000039926	3.4	0
NUDCD3	NUDCD3	3.4	0
LGR5	LGR5	3.3	0
LOC102162336	ENSSSCG00000032301	3.3	0.03
ENSSSCG00000041780	ENSSSCG00000041780	3.3	0
CTSZ	CTSZ	3.3	0.03
TMEM151A	TMEM151A	3.3	0
GLIS2	GLIS2	3.3	0.02
DLX6	DLX6	3.3	0
BDH1	BDH1	3.3	0.04
MFGE8	MFGE8_tv3	3.3	0
HUWE1	HUWE1_tvX1	3.3	0
KCNQ2	KCNQ2	3.3	0.02
SMAD7	SMAD7	3.3	0
NOTCH1	NOTCH1	3.3	0
NES	NES	3.3	0

SPHK1	SPHK1_tv2	3.2	0
GSN	GSN_tv2	3.2	0.02
ENSSSCG00000048105	ENSSSCG00000048105	3.2	0
CROCC	CROCC_tv	3.2	0
LncRNA	LncRNA	3.2	0
ENSSSCG00000044645	ENSSSCG00000044645	3.2	0
FLRT2	FLRT2_tvX1	3.2	0.02
ENSSSCG00000018657	ENSSSCG00000018657	3.2	0
EHF	EHF	3.2	0.02
NAV1	NAV1_tvX8	3.2	0.01
PTCH1	PTCH1_tv8	3.2	0
SRL	SRL	3.2	0.04
THBS4	THBS4	3.2	0
RRBP1	RRBP1	3.1	0
NTF3	NTF3	3.1	0
DVL1	DVL1_tvX1	3.1	0.01
FCRL5	FCRL5	3.1	0.04
FGFR1	FGFR1	3.1	0
RNF183	RNF183	3.1	0.02
LRP1	LRP1	3.1	0
LOC110255791	ENSSSCG00000044808	3.1	0
PKNOX2	PKNOX2	3.1	0
SLC1A7	SLC1A7	3.1	0.01
ENSSSCG00000051523	ENSSSCG00000051523	3.1	0.02
ENSSSCG00000026908	ENSSSCG00000026908	3.1	0
LAMA5	ENSSSCG00000032761	3.1	0
LRRC8D	LRRC8D	3.1	0
CSMD1	CSMD1_tvX1	3.1	0
ADAMTS16	ADAMTS16_tv1	3.1	0
SLC6A9	SLC6A9	3.1	0
MAPKAPK2	MAPKAPK2	3	0
ENSSSCG00000043549	ENSSSCG00000043549	3	0.01
INAVA	INAVA	3	0.01
SALL3	SALL3_tv1	3	0
KIT	KIT_tv3	3	0
NTRK3	NTRK3_tv1	3	0.02
LOC110260611	ENSSSCG00000035352	3	0
ICAM5	ICAM5_tvX1	3	0.01
TARDBPPs*	TARDBPPs*	3	0.04
TNRC18	TNRC18	3	0
DSCAM	DSCAM	3	0
KMT2D	KMT2D	3	0
SYNGAP1	SYNGAP1	3	0
LOC100513133	ENSSSCG00000044913	3	0
NEFH	NEFH	3	0.02
LOC107984638	LOC107984638	3	0.05
LOC110256821	ENSSSCG00000048625	3	0.04

PLAT	PLAT_tv1	3	0
LLGL2	LLGL2	3	0
MYH6	MYH6	3	0
SF1	SF1_tv6	3	0.03
ZBTB4	ZBTB4_tv2	3	0.02
ABCA13	ABCA13_tv1	3	0
ENSSSCG00000046488	ENSSSCG00000046488	3	0.05
COL22A1	COL22A1_tv1	3	0
GRIN3B	GRIN3B_tv1	2.9	0
AGRN	AGRN	2.9	0
lncRNA	lncRNA	2.9	0
PRKAR1B	PRKAR1B	2.9	0
HRH2	HRH2_tv1	2.9	0
CSPG4	CSPG4	2.9	0
GNB3	GNB3	2.9	0.01
TRPM4	TRPM4	2.9	0
UPF1	UPF1_tv2	2.9	0.03
GRIN2A	GRIN2A_tv1	2.9	0
ABLIM2	ABLIM2_tv1	2.9	0.01
JAK1	JAK1_tv3	2.9	0
SPTBN4	ENSSSCG00000037494	2.9	0.02
KLHL35	KLHL35	2.9	0.01
BCL9L	BCL9L	2.9	0
LOC110257830	LOC110257830	2.9	0
B3GAT1	B3GAT1	2.9	0
VWF	VWF	2.9	0
KCNK12	KCNK12	2.9	0
DYNC1H1	DYNC1H1	2.9	0
KCNIP2	KCNIP2	2.8	0
MIR2887-2	MIR2887-2	2.8	0.04
GRIK5	GRIK5	2.8	0
NSD2	NSD2	2.8	0
STK24	STK24	2.8	0
SHANK2	SHANK2_tv1	2.8	0
PKD1	PKD1	2.8	0
MAP1A	MAP1A	2.8	0
WSCD2	WSCD2	2.8	0
ABCC6	ABCC6_tv1	2.8	0.01
ITGA7	ITGA7	2.8	0
ZC3H18	ZC3H18	2.8	0
OXTR	OXTR_tv1	2.8	0.01
COL4A2	COL4A2	2.8	0
NOVA2	NOVA2	2.8	0.02
NAV2	NAV2	2.8	0
POLR2A	POLR2A	2.8	0
LAMC1	LAMC1	2.8	0
ARHGAP39	ARHGAP39	2.8	0

FGFR2	FGFR2	2.8	0
CEP250	CEP250	2.8	0
APLNR	APLNR	2.8	0
EXOC3L2	ENSSSCG00000040410	2.8	0
SRCAP	SRCAP_tv1	2.8	0
ADAMTS7	ADAMTS7	2.8	0
GPR162	GPR162_tvA	2.8	0.01
CSF1	CSF1_tv1	2.8	0.01
MAP2K7	MAP2K7	2.8	0
TTBK1	TTBK1	2.8	0
TNFRSF6B	ENSSSCG00000036595	2.8	0
RASSF5	RASSF5	2.8	0
COL9A2	COL9A2	2.8	0
LRRC3	LRRC3	2.8	0
DENND2B	DENND2B	2.8	0
PPFIBP2	PPFIBP2	2.7	0
DYSF	DYSF	2.7	0
ERBB3	ERBB3_tv1	2.7	0
ESPN	ESPN	2.7	0
LOC102165318	LOC102165318	2.7	0
TNR	TNR	2.7	0
NMNAT3	NMNAT3	2.7	0
PCNT	PCNT_tv1	2.7	0
GLIS1	ENSSSCG00000003846	2.7	0
CDH24	CDH24_tv2	2.7	0
HIPK4	HIPK4	2.7	0.02
PTAFR	PTAFR	2.7	0.02
SORL1	SORL1	2.7	0
SH3BP1	SH3BP1	2.7	0.01
LOC106509601	ENSSSCG00000042596	2.7	0.01
TNN	TNN	2.7	0
MOB2	MOB2	2.7	0
ENSSSCG00000044428	ENSSSCG00000044428	2.7	0
CACNA1E	CACNA1E_tv1	2.7	0
SCARNA12	SCARNA12	2.7	0.02
SYT3	SYT3	2.7	0
MXRA5	ENSSSCG00000012832	2.7	0
AP2A1	AP2A1	2.7	0
GSG1L	GSG1L_tv1	2.7	0.01
PPP1R12B	PPP1R12B	2.7	0
PENK	PENK	2.7	0
SOGA1	SOGA1	2.7	0
ELAC2	ELAC2	2.7	0
HCFC1	ENSSSCG00000012793	2.7	0
NEURL1B	NEURL1B	2.7	0
AGPAT3	AGPAT3	2.7	0
CLCF1	CLCF1	2.7	0

ZFHX2	ZFHX2_tv1	2.7	0.01
SHB	SHB	2.7	0
CRTC1	CRTC1	2.7	0
NBEAL2	NBEAL2_tv1	2.7	0
SLC9A2	SLC9A2	2.7	0.05
DIDO1	DIDO1_tv3	2.7	0
FPGS	FPGS	2.7	0.02
IGSF3	IGSF3_tv1	2.6	0
LTBP4	LTBP4	2.6	0
USP12	USP12	2.6	0
VWA2	VWA2	2.6	0.03
HTR4	HTR4	2.6	0
ZNF316	ZNF316	2.6	0
TCF7	TCF7	2.6	0
CELSR2	CELSR2	2.6	0
ANKRD11	ANKRD11	2.6	0
SEC14L1	SEC14L1	2.6	0
TLN1	TLN1	2.6	0
PIEZO1	PIEZO1	2.6	0
BCORL	BCORL	2.6	0.04
SPEN	SPEN	2.6	0
JAG2	JAG2_tv1	2.6	0
VDR	VDR_tvt	2.6	0.03
ADARB2	ADARB2	2.6	0.01
LTBP3	LTBP3	2.6	0
SYT15	ENSSSCG00000028341	2.6	0.02
MYH15	MYH15	2.6	0.02
KBTBD4	KBTBD4_tv2	2.6	0.05
ABCC10	ABCC10	2.6	0
ENSSSCG00000030555	ENSSSCG00000030555	2.6	0.02
CASZ1	CASZ1	2.6	0.04
SYNE1	SYNE1_tvX11	2.6	0.04
DOCK6	DOCK6_tv2	2.6	0.02
LAMA1	LAMA1_tv1	2.6	0
PIK3R2	PIK3R2	2.6	0
ARID1A	ARID1A_tv1	2.6	0
LMOD1	LMOD1	2.6	0
ENSSSCG00000042625	ENSSSCG00000042625	2.6	0.04
HLX	HLX_tv1	2.6	0
BCORL1	BCORL1	2.6	0
NLRC5	NLRC5	2.6	0.02
CNTNAP5	CNTNAP5_tvX1	2.6	0.02
TRAF4	TRAF4	2.6	0.02
KMT2B	KMT2B_tv1	2.6	0
RNF216	RNF216	2.6	0
CNTRL	CNTRL_tv1	2.6	0.03
SORCS2	SORCS2	2.6	0

CASKIN1	CASKIN1	2.6	0
SLC22A17	SLC22A17	2.6	0
TAS1R3	TAS1R3_tv	2.6	0.05
ZNF385C	ZNF385C	2.6	0.04
WNT11	WNT11	2.6	0.01
LAMC3	LAMC3_tv1	2.6	0
BCAM	BCAM_tv1	2.6	0
GRB10	GRB10	2.6	0
CREBBP	CREBBP_tv1	2.6	0
NR5A2	NR5A2	2.5	0
NCDN	NCDN_tv3	2.5	0.01
HPDL	HPDL	2.5	0.01
NCOR2	NCOR2_tv3	2.5	0
EPN2	EPN2_tv2	2.5	0
SLX4	SLX4_tv1	2.5	0
LRP4	LRP4_tv1	2.5	0
SCD5	SCD5_tv1	2.5	0
NDST2	ENSSSCG00000010310	2.5	0.01
SBK2	SBK2	2.5	0.02
ADGRL1	ADGRL1_tv1	2.5	0
NOTCH2	NOTCH2	2.5	0
LAMB3	LAMB3_tv1	2.5	0
HIVEP3	HIVEP3	2.5	0.02
KLHL29	KLHL29	2.5	0
ARHGEF17	ARHGEF17_tvX1	2.5	0
PRR12	PRR12_tv1	2.5	0
PRDM16	PRDM16	2.5	0.03
ZBTB46	ZBTB46_tvX3	2.5	0
SH2B2	SH2B2	2.5	0.03
NWD1	NWD1	2.5	0.02
SEPTIN8	SEPTIN8	2.5	0
CHD5	CHD5	2.5	0.02
MEGF11	MEGF11	2.5	0
TCOF1	TCOF1_tvX3	2.5	0
WSB2	WSB2	2.5	0
PAXIP1	PAXIP1	2.5	0
SKI	SKI	2.5	0
SAMD11	SAMD11	2.5	0
CTBP2	CTBP2	2.5	0
LGALS3BP	LGALS3BP	2.5	0.02
SLC52A3	SLC52A3	2.5	0
TSPAN15	TSPAN15_tv1	2.5	0
SLC26A10	SLC26A10	2.5	0
TFEB	TFEB	2.5	0
MKI67	ENSSSCG00000026302	2.5	0
ADRA1D	ADRA1D	2.5	0.01
SRRM2	SRRM2	2.5	0

KIAA1549	ENSSSCG00000016513	2.5	0
ACOT11	ACOT11_tv1	2.5	0
SLC22A23	SLC22A23	2.5	0
CACNA2D4	CACNA2D4	2.5	0.03
PPM1H	PPM1H	2.5	0
RPS6KA2	RPS6KA2	2.5	0
CYP2S1	CYP2S1	2.5	0
FBLN2	FBLN2	2.5	0
MAP4K2	MAP4K2	2.5	0.03
PDGFRB	PDGFRB	2.5	0
LncRNA	LncRNA	2.5	0.01
CHD7	CHD7_tv1	2.5	0
ARHGEF40	ARHGEF40	2.5	0
LOC102166195	ENSSSCG00000038005	2.5	0.02
NCOR2	NCOR2	2.5	0
TET3	TET3	2.5	0
CACNA1C	ENSSSCG00000027725	2.5	0
COL4A1	COL4A1	2.5	0
AP2A2	AP2A2	2.5	0
CACNG4	CACNG4	2.5	0
PLCB3	PLCB3	2.5	0
MYH7B	MYH7B_tv1	2.5	0
MED24	MED24	2.5	0
SIPA1	SIPA1	2.5	0
ZBTB42	ZBTB42	2.5	0.03
IL17D	IL17D	2.5	0
COL27A1	COL27A1_tv1	2.5	0
TBX1	TBX1	2.4	0
BEGAIN	BEGAIN	2.4	0.05
MYH14	MYH14_tv1	2.4	0.02
NRCAM	NRCAM	2.4	0.02
SEC16A	SEC16A	2.4	0
SPTB	SPTB	2.4	0.01
COL6A1	COL6A1	2.4	0
WDR1	WDR1	2.4	0
PLXNA1	PLXNA1	2.4	0
ELFN1	ENSSSCG00000048398	2.4	0.03
FLNA	FLNA_tv1	2.4	0
CCL22	CCL22_tv1	2.4	0.01
EBP	ENSSSCG00000032768	2.4	0.02
SPATA21	SPATA21	2.4	0.03
BPTF	BPTF_tv1	2.4	0.01
MRC2	MRC2	2.4	0
SCARNA2	SCARNA2	2.4	0.02
PLXND1	PLXND1	2.4	0
KIF7	KIF7	2.4	0
SLC6A17L*	ENSSSCG00000038264	2.4	0.01

WDR86	WDR86_tv2	2.4	0
CD248	CD248	2.4	0
ENSSSCG00000051462	ENSSSCG00000051462	2.4	0.01
H19	H19_tv1	2.4	0
HSD17B6	HSD17B6	2.4	0.03
KIFC3	KIFC3	2.4	0
MPP2	MPP2	2.4	0.04
EDAR	EDAR	2.4	0.01
TRAF3IP1	TRAF3IP1	2.4	0
FAIM2	FAIM2	2.4	0
NIPAL4	NIPAL4	2.4	0.01
HNRNPUL2	HNRNPUL2	2.4	0
RAI1	RAI1	2.4	0
LOC102159171	ENSSSCG00000035711	2.4	0.02
COL13A1	COL13A1_tv1	2.4	0
SLPI	SLPI	2.4	0.03
CHD3	CHD3_tv1	2.4	0
ETV4	ETV4_tv2	2.4	0
SHROOM2	SHROOM2	2.4	0
CHID1	CHID1	2.4	0
FASN	FASN	2.4	0
ITGA3	ITGA3_tva	2.4	0
RBM19	RBM19	2.4	0
PPARD	PPARD	2.4	0
ASTN1	ASTN1_tvX1	2.4	0
RCVRN	RCVRN	2.4	0.02
DLGAP3	DLGAP3	2.4	0
MYH3	MYH3	2.4	0.01
UNC5B	UNC5B	2.4	0
TRAF5	TRAF5	2.4	0.05
DFNB31	DFNB31	2.4	0.02
LOC110255292	ENSSSCG00000043013	2.4	0
PCDH12	PCDH12	2.4	0
CLPB	CLPB	2.4	0
LOC110261123	ENSSSCG00000047147	2.4	0
PLXNA2	PLXNA2	2.4	0
TENM4	TENM4	2.4	0
BICRA	BICRA	2.4	0
SOGA2	SOGA2	2.4	0.01
PIAS4	PIAS4	2.4	0
CCDC102A	CCDC102A	2.4	0
PTPN6	PTPN6	2.4	0
LRP8	LRP8	2.3	0
SETD1B	SETD1B_tvX1	2.3	0
PEPD	PEPD	2.3	0
MEGF8	MEGF8	2.3	0
DPP6	DPP6	2.3	0

VANGL2	VANGL2	2.3	0
NPTX1	NPTX1	2.3	0.01
EP400	EP400	2.3	0
BRD4	BRD4_tv1	2.3	0
RTN4RL1	RTN4RL1	2.3	0
PRRC2A	PRRC2A_tv	2.3	0
MAPK12	MAPK12	2.3	0.01
IGFBP5	IGFBP5	2.3	0
SHANK1	SHANK1_tv1	2.3	0.03
PPP1R37	PPP1R37	2.3	0
KCNB1	KCNB1	2.3	0.01
LOC102161632	ENSSSCG00000045748	2.3	0.04
SELENOO	SELENOO	2.3	0
GPR35	GPR35	2.3	0.01
CLCN6	CLCN6_tv1	2.3	0
LPP	LPP_tv	2.3	0
NEDD9	NEDD9	2.3	0
MEIS3	MEIS3	2.3	0.02
MAP2K3	MAP2K3	2.3	0
SUN1	SUN1	2.3	0
MIR4332	MIR4332	2.3	0.05
ADARB1	ADARB1_tv1	2.3	0
PKN1	PKN1_tv2	2.3	0.01
ENSSSCG00000047144	ENSSSCG00000047144	2.3	0
RGS7BP	RGS7BP	2.3	0.03
ENSSSCG00000051360	ENSSSCG00000051360	2.3	0.01
ZC3H13	ZC3H13_tvX8	2.3	0
STARD9	ENSSSCG00000022039	2.3	0
ROBO4	ROBO4	2.3	0
MYH10	ENSSSCG00000038144	2.3	0
ENG	ENG_tv1	2.3	0
L1TD1	L1TD1	2.3	0
SH3PXD2A	SH3PXD2A	2.3	0
H6PD	H6PD	2.3	0
CA11	CA11_tv1	2.3	0
PPRC1	PPRC1	2.3	0
THRA	THRA_tv1	2.3	0
IGF1R	IGF1R_tv	2.3	0
RTN4RL2	RTN4RL2	2.3	0.01
NR1H2	NR1H2	2.3	0.01
UNKL	ENSSSCG00000022963	2.3	0
ETV6	ETV6	2.3	0
ANKRD24	ANKRD24	2.3	0
HMGN5	HMGN5	2.3	0
ZNF469	ZNF469_tvX1	2.3	0.03
JUP	JUP	2.3	0
SCMH1	SCMH1	2.3	0

JPH4	JPH4_tv1	2.3	0
CHST1	CHST1	2.3	0
WNT3	WNT3	2.3	0
CCL24	CCL24_tv1	2.3	0.01
lncRNA	lncRNA	2.3	0.02
HECTD4	HECTD4_tv1	2.3	0
TAF3	TAF3	2.3	0
ANXA10	ANXA10	2.3	0.03
HPX	HPX	2.3	0.01
SLC6A17	SLC6A17	2.3	0
ZNF853	ZNF853	2.3	0
UVSSA	UVSSA	2.3	0.02
DIS3L2	DIS3L2	2.3	0
HIRIP3	HIRIP3_tv1	2.3	0
NID2	NID2	2.3	0
CCDC22	CCDC22	2.3	0
MDN1	MDN1	2.3	0
SEMA4D	SEMA4D	2.3	0.01
FAT3	FAT3_tv1	2.3	0.02
BCAR3	BCAR3	2.3	0
LOC110256059	ENSSSCG00000046684	2.3	0.01
CD68	CD68	2.3	0.04
HEY2	HEY2	2.3	0
OTUB1	OTUB1	2.3	0.03
POM121C	POM121C	2.3	0
GTF3C1	GTF3C1	2.3	0
FAM83H	FAM83H	2.2	0
FAM53A	FAM53A	2.2	0.01
FYN	FYN	2.2	0
ADGRA2	ADGRA2_tv1	2.2	0
TUB	TUB	2.2	0.05
CRAMP1	CRAMP1	2.2	0
GAREM2	GAREM2	2.2	0.01
ZNF541	ZNF541	2.2	0
ACE	ACE_tv1	2.2	0
NCK2	NCK2	2.2	0
ARFGEF3	ARFGEF3	2.2	0.01
LCMT1L3*	LCMT1L3*	2.2	0
KCNQ1	KCNQ1	2.2	0
CAPZB	CAPZB	2.2	0
MED12	MED12	2.2	0
ANKH	ANKH	2.2	0
TRIM26	TRIM26	2.2	0
ZNF890	ENSSSCG00000033648	2.2	0.02
DOT1L	DOT1L_tvX1	2.2	0
GNAO1	GNAO1	2.2	0.01
ENSSSCG00000051028	ENSSSCG00000051028	2.2	0.03

FOXO6	FOXO6	2.2	0
NFRKB	NFRKB	2.2	0
RNF165	RNF165	2.2	0.01
GMEB2	GMEB2	2.2	0
AMZ1	AMZ1	2.2	0.01
LY9L6	LY9L6_tv1	2.2	0
PRRC2B	PRRC2B	2.2	0
ELAVL4	ELAVL4	2.2	0.01
ADGRG5	ADGRG5	2.2	0
TRIM71	TRIM71	2.2	0
LSG1	LSG1	2.2	0.03
FADD	FADD	2.2	0
LRP2	LRP2	2.2	0.02
WNT5B	WNT5B_tv1	2.2	0.02
TMEM214	TMEM214_tv2	2.2	0
DCHS2	DCHS2_tv3	2.2	0.02
ZNF609	ZNF609_tv1	2.2	0
ABCA14	ABCA14	2.2	0.03
SMAD3	SMAD3	2.2	0
DAAM2	DAAM2_tv1	2.2	0
SFRP1	SFRP1	2.2	0.01
AMPD2	AMPD2	2.2	0.02
FGF1	FGF1_tv1	2.2	0.03
GATD3A	GATD3A	2.2	0
CDH4	CDH4_tv1	2.2	0
SYNPO2L	SYNPO2L	2.2	0
CHD6	CHD6	2.2	0
TNK2	TNK2_tv1	2.2	0
PWWP2B	PWWP2B	2.2	0.03
ZFHX3	ZFHX3_tv1	2.2	0.01
SAFB	SAFB_tv2	2.2	0
MAP1B	MAP1B_tv1	2.2	0
NCOR1	NCOR1_tvX37	2.2	0
SAMD4B	SAMD4B_tv1	2.2	0.01
SDK2	SDK2_tvX1	2.2	0
PDXK	PDXK	2.2	0
FANCA	FANCA	2.2	0
GRK2	GRK2	2.2	0.01
SCARF2	SCARF2	2.2	0
AMH	AMH	2.2	0
FAT1	FAT1_tv1	2.2	0
BEND4	BEND4	2.2	0.01
ENSSSCG00000048975	ENSSSCG00000048975	2.2	0.03
HR	HR	2.2	0
HTRA1	HTRA1	2.2	0
ZBTB20	ZBTB20_tv1	2.2	0
HIVEP1	HIVEP1	2.2	0

CCDC88C	CCDC88C	2.2	0
NFIC	NFIC	2.2	0
SLC35C2	SLC35C2	2.2	0.02
PRRC2C	PRRC2C	2.2	0
RAP1GAP2	ENSSSCG00000034858	2.2	0
MACF1	MACF1_tv1	2.2	0
SYNPO	SYNPO	2.2	0
NTM	NTM	2.2	0
EPHB3	EPHB3	2.2	0
TOB2	TOB2_tvX1	2.2	0.01
DST	DST	2.2	0
lncRNA	lncRNA	2.2	0
CYTH1	CYTH1	2.2	0
DHRSX	ENSSSCG00000039144	2.2	0
PTCH2	PTCH2_tv1	2.2	0
SPATA13	SPATA13_tv3	2.2	0
LOC106510465	ENSSSCG00000042592	2.2	0.02
ZNF646	ZNF646	2.2	0
ST3GAL3	ST3GAL3	2.2	0
SRGAP1	SRGAP1	2.2	0
ZNF503	ZNF503	2.2	0
STK11IP	STK11IP	2.2	0.02
UBR4	UBR4	2.2	0
PHRF1	PHRF1	2.2	0
NAV3	NAV3	2.2	0.01
HBZ	HBZ	2.2	0
CARD11	CARD11_tv2	2.2	0.05
CD44	CD44	2.2	0.04
ADAMTSL2	ADAMTSL2	2.2	0.02
OSBPL5	OSBPL5_tv1	2.1	0
FOXP4	FOXP4	2.1	0
ACTR1B	ACTR1B	2.1	0.02
UBA1	UBA1	2.1	0
STARD13Ps*	ENSSSCG00000046579	2.1	0.01
BCL3	BCL3_tvX1	2.1	0
FCHO1	FCHO1	2.1	0.01
PRDM2	PRDM2_tv	2.1	0
SSC5D	SSC5D_tv1	2.1	0
ADCY9	ADCY9	2.1	0
MICAL3	MICAL3	2.1	0
KCNQ3	KCNQ3	2.1	0
CCNJL	CCNJL	2.1	0
UMODL1	UMODL1	2.1	0.03
BAHCC1	ENSSSCG00000023045	2.1	0
PEG3	PEG3_tv1	2.1	0
ELF4	ELF4_tv1	2.1	0
TNS3	TNS3_tv1	2.1	0

MPRIP	MPRIP	2.1	0
COL5A1	COL5A1	2.1	0
ZNF618	ZNF618	2.1	0
PTPRF	PTPRF	2.1	0
ADGRL3	ADGRL3_tv1	2.1	0
CDH5	CDH5	2.1	0
TSPAN4	TSPAN4	2.1	0
TRAM2	TRAM2	2.1	0
EML3	EML3	2.1	0
ST3GAL4	ST3GAL4	2.1	0
IKZF4	IKZF4	2.1	0
HSPG2	HSPG2	2.1	0
KCNJ4	KCNJ4_tv1	2.1	0.03
PTMS	PTMS	2.1	0
FOXF1	FOXF1	2.1	0.04
PFKM	PFKM	2.1	0.01
DGKZ	DGKZ	2.1	0
CTBP1	CTBP1	2.1	0
ERCC4	ERCC4	2.1	0.03
ZBED6CL	ZBED6CL	2.1	0
TBL1X	ENSSSCG00000012102	2.1	0
GLG1	GLG1_tv	2.1	0
LASP1	LASP1	2.1	0
C11orf95	C11orf95	2.1	0
USP36	USP36	2.1	0
PLXNB2	PLXNB2	2.1	0
MSC	MSC	2.1	0
SPRY4	SPRY4	2.1	0
ZNF3L*	ENSSSCG00000031485	2.1	0.03
HCK	HCK	2.1	0.03
LIPE	LIPE	2.1	0.01
COL15A1	COL15A1_tvX1	2.1	0
CBFA2T3	CBFA2T3_tv1	2.1	0.01
GRAMD4	GRAMD4	2.1	0
NIBAN2	NIBAN2	2.1	0.01
LDLRAD2	LDLRAD2	2.1	0
VANGL1	VANGL1	2.1	0
CLCN2	CLCN2	2.1	0
AFDN	AFDN_tvX10	2.1	0
VAC14	VAC14	2.1	0
CDR2	CDR2	2.1	0
MEGF6	MEGF6	2.1	0.03
PPP2R2B	PPP2R2B	2.1	0.03
SMURF1	SMURF1_tv2	2.1	0
STK39	STK39	2.1	0
MYO10	MYO10_tv1	2.1	0
CCDC158	CCDC158	2.1	0.05

NACC2	NACC2_tv1	2.1	0
HTR1D	HTR1D_tv1	2.1	0
WWC1	WWC1	2.1	0.03
TNFRSF11A	TNFRSF11A	2.1	0
TNS1	TNS1	2.1	0
ARID3A	ARID3A_tvX1	2.1	0
PROX1	PROX1	2.1	0.05
SMAD6	SMAD6	2.1	0.01
TRIM66	TRIM66_tv1	2.1	0
FZD8	FZD8	2.1	0
RCC2	RCC2	2.1	0
AKNA	AKNA_tv1	2.1	0
DCTN1	DCTN1_tv1	2.1	0.01
SH3TC1	SH3TC1	2.1	0
CLCN7	CLCN7	2.1	0
DPYSL5	DPYSL5	2.1	0
GPR4	GPR4	2.1	0
EMILIN1	EMILIN1	2.1	0
ENSSSCG00000050854	ENSSSCG00000050854	2.1	0
PLEKHG2	PLEKHG2_tvX3	2.1	0
PSD3	PSD3_tv1	2.1	0
BRD3	BRD3	2.1	0
COL6A2	COL6A2_tv2C2	2.1	0
PER2	PER2	2.1	0
EXOC3L2	EXOC3L2	2.1	0
TNFAIP2	TNFAIP2	2.1	0
AGO2	AGO2_tv1	2.1	0
MYLK3	MYLK3	2.1	0
AACS	AACS	2.1	0.01
GANAB	GANAB	2.1	0
RAPH1	RAPH1_tv1	2.1	0
PDZD4	PDZD4_tv1	2.1	0.02
PAPLN	PAPLN	2.1	0
ZSCAN20	ENSSSCG00000042252	2.1	0
GAS7	GAS7_tvc	2.1	0
EHBP1L1	EHBP1L1_tvX1	2.1	0
SLC45A4	SLC45A4	2.1	0.02
KIAA0556	KIAA0556_tv1	2.1	0
TRIM56	TRIM56_tv1	2.1	0
RTN4R	RTN4R	2.1	0.02
ATG2A	ATG2A_tv	2.1	0
ZNF516	ZNF516	2.1	0
ARHGAP23	ARHGAP23	2.1	0
IGF2R	IGF2R	2.1	0
ZNF704	ZNF704	2.1	0
MEX3D	MEX3D	2.1	0
FRMD4A	FRMD4A	2.1	0

PGLYRP3	PGLYRP3_tvX1	2.1	0.02
MZF1	MZF1_tv2	2.1	0.04
KCNK6	KCNK6	2.1	0
GATS	GATS	2.1	0
ST5	ST5_tv3	2.1	0
CD93	CD93	2.1	0
AGAP2	AGAP2_tv2	2.1	0
WNT9A	WNT9A	2.1	0.01
HTR7	HTR7	2.1	0.02
EVC2	EVC2	2.1	0
SLC1A3	SLC1A3	2.1	0.02
L1CAM	L1CAM_tv1	2.1	0.03
KMT2C	KMT2C_tvX1	2.1	0
PLXNB1	PLXNB1_tv2	2.1	0
PLCD1	PLCD1	2.1	0.01
TMEM120B	TMEM120B	2.1	0.04
ABCA4	ABCA4	2.1	0.03
PIGZ	PIGZ	2.1	0.03
WFDC2	WFDC2	2.1	0.04
GPR173	GPR173	2.1	0
TONSL	TONSL_tv1	2.1	0
DSP	DSP	2.1	0
FYCO1	FYCO1	2.1	0
SPTBN1	SPTBN1	2.1	0
NECTIN1	NECTIN1	2.1	0
TANC2	TANC2	2.1	0
PLEKHA7	PLEKHA7	2.1	0
ITGA2B	ITGA2B	2.1	0.02
SERGEF	SERGEF	2.1	0
FBN3	FBN3	2.1	0
PPP2R5D	PPP2R5D	2.1	0.01
UBE2O	UBE2O	2.1	0
LOC102164640	ENSSSCG00000048792	2.1	0.01
PRKCD	PRKCD	2.1	0.01
GPR153	GPR153	2.1	0
AQP5	AQP5	2.1	0.04
MAML3	MAML3	2.1	0
DOC2B	DOC2B	2.1	0
LMTK2	LMTK2	2	0
TIMP2	ENSSSCG00000017164	2	0
SYNE3	SYNE3	2	0
SGSM1	SGSM1	2	0
CSNK1D	CSNK1D	2	0
VAPB	VAPB	2	0
RIPOR1	RIPOR1	2	0
CELF4	CELF4	2	0
CELF2	CELF2	2	0

HOXC8	HOXC8	2	0.02
TNKS1BP1	ENSSSCG00000035585	2	0
SLC5A6	SLC5A6	2	0.01
OC90	ENSSSCG00000005954	2	0.02
KIAA0930	KIAA0930	2	0.03
SLC38A10	SLC38A10_tv1	2	0
PRODH2	ENSSSCG00000002913	2	0.04
SHANK3	SHANK3	2	0
FRMPD3	FRMPD3	2	0.05
TNRC6C	TNRC6C	2	0
KLHDC4	KLHDC4_tv1	2	0
ABCC10	ABCC10_tvMRP7	2	0
RIMS4	RIMS4	2	0
MKL1	MKL1_tv4	2	0
ARHGEF10	ENSSSCG00000023419	2	0
ELFN1	ELFN1	2	0.03
LOC110257830	ENSSSCG00000041898	2	0.01
CARD14	CARD14_tv1	2	0.02
KIF3C	KIF3C	2	0
ITIH5	ITIH5	2	0
EIF5B	EIF5B	2	0
WFS1	WFS1	2	0
ZNF526	ZNF526	2	0
UVRAG	UVRAG	2	0
PRKCA	PRKCA	2	0
FGFR4	FGFR4	2	0
DNASE1	DNASE1	2	0.01
PCDH8	PCDH8	2	0.02
PFKL	PFKL	2	0.04
PHACTR1	PHACTR1	2	0
GDI1	GDI1	2	0.01
CD99L2	CD99L2	2	0
TOP3A	TOP3A	2	0.01
ANKRD52	ANKRD52	2	0
SLC2A3	SLC2A3	2	0
SDK1	SDK1	2	0
UHRF1	UHRF1	2	0.01
ENSSSCG00000047008	ENSSSCG00000047008	2	0.05
HDGFRP2	HDGFRP2_tv1	2	0
GPM6B	GPM6B	2	0
CASKIN2	CASKIN2_tv1	2	0
SETD3	SETD3	2	0
INTS1	INTS1	2	0
LDLRAP1	LDLRAP1	2	0.01
EIF3A	EIF3A	2	0
CDK18	CDK18_tv3	2	0
TRAF7	TRAF7	2	0.01

TSPAN9	TSPAN9_tv1	2	0
BNIP3	BNIP3	2	0
HIPK2	HIPK2	2	0
SLC22A3	SLC22A3	2	0.01
EMILIN2	EMILIN2	2	0.05
SPTAN1	SPTAN1_tv	2	0
RN7SK	RN7SK	2	0.03
FAM160A1	FAM160A1	2	0.01
KNDC1	KNDC1	2	0.03
ZXDC	ZXDC	2	0
PTK2	PTK2	2	0
WDR81	WDR81	2	0
NLGN3	NLGN3	2	0.02
CENPE	CENPE_tv1	2	0.02
WNK2	WNK2	2	0
TMEM94	TMEM94	2	0
CAMSAP2	CAMSAP2	2	0
FAM43A	FAM43A	2	0
TLCD3B	TLCD3B	2	0.03
TBKBP1	TBKBP1_tv1	2	0
PEAK1	PEAK1	2	0
PRDM15	PRDM15	2	0
PTK2B	PTK2B_tv1	2	0
ITGA5	ITGA5	2	0.02
NCL	NCL	2	0
N4BP3	N4BP3	2	0
DAGLA	DAGLA_tv1	2	0.01
KIRREL1	KIRREL1	2	0
ZNF467	ZNF467_tv1	2	0.01
TRMT44	TRMT44	2	0
TTC7B	TTC7B	2	0
LOC106507059	LOC106507059	2	0
RAB40C	RAB40C	2	0.01
PPP1R9B	PPP1R9B	2	0
ELMO2	ELMO2	2	0
ARHGAP6	ARHGAP6	2	0.02
HTT	HTT	2	0
LOC106507406	LOC106507406	2	0.01
ATP1B2	ATP1B2_tv1	2	0
TRIM8	TRIM8	2	0
SMOX	SMOX	2	0.01
BRD9	BRD9	2	0
MTUS2	MTUS2	2	0
C2CD3	C2CD3_tv1	2	0.02
ABCC1	ABCC1	2	0
ZCCHC14	ZCCHC14_tv1	2	0
ASIC2	ASIC2	2	0

WSCD1	WSCD1	2	0
NFIX	NFIX	2	0
LRRC4B	LRRC4B	2	0
KBTBD11	KBTBD11	2	0.03
TSPOAP1	TSPOAP1	2	0
MLLT6	MLLT6	2	0
MTOR	MTOR_tv1	2	0
SCRIB	SCRIB_tv1	2	0
MUC3A	MUC3A	2	0.01
OSBPL2	OSBPL2	2	0
GRM7	GRM7_tv1	2	0.01
ABL1	ABL1_tvb	2	0
SMARCC2	SMARCC2	2	0
PHC2	PHC2	2	0
PTPN14	PTPN14	2	0
FAM124B	FAM124B	2	0.01
ITPR3	ITPR3	2	0
MAP7D1	MAP7D1_tv1	2	0
DEF6	DEF6	2	0.01
ZFAT	ZFAT	2	0
SMOC2	SMOC2_tv1	2	0.03
ENSSSCG00000049992	ENSSSCG00000049992	2	0.01
PRAG1	PRAG1_tvX1	2	0
LMF1	LMF1	2	0
CNNM1	CNNM1	2	0
LUZP1	LUZP1_tv2	2	0
HPSE	HPSE_tv2	2	0.01
FGD2	FGD2	2	0.01
ARAP3	ARAP3	2	0
MICAL2	MICAL2	2	0
PCNXL3	PCNXL3_tvX4	2	0
SLC12A9	SLC12A9_tv1	2	0
ARAP1	ARAP1	2	0
DAB2IP	DAB2IP	2	0
PTPN23	PTPN23_tvX1	2	0
TRRAP	TRRAP	2	0
ADCY7	ADCY7	2	0.05
MMP2	MMP2_tv1	2	0
SLC16A2	SLC16A2	2	0.03
ABCA7	ABCA7	2	0
ZC3H4	ZC3H4	2	0
LAMB2	LAMB2	2	0
CHD4	CHD4	2	0
BAZ2A	BAZ2A	2	0
TCF20	TCF20_tv2	2	0
TM4SF19	TM4SF19	2	0
DOCK3	DOCK3	2	0

SLC9A5	SLC9A5	2	0.02
AKAP11	AKAP11	2	0.02
PLXNA3	PLXNA3	2	0
STRBP	STRBP	2	0
SLC9A1	SLC9A1_tv1	2	0
PDE2A	PDE2A	2	0
TRIOBP	TRIOBP_tv1	2	0
CFDP1	CFDP1	2	0
SCAF1	SCAF1_tv1	2	0
ARNTL	ARNTL	2	0
SPSB1	SPSB1	2	0.02
BCOR	BCOR	2	0
GGT1	ENSSSCG00000010056	2	0
ZNF276	ZNF276_tva	2	0
FLT1	FLT1_tv1	2	0
MAML2	MAML2	2	0
SIAH2	SIAH2	2	0.04
GALNT17	GALNT17	2	0
PIEZO2	PIEZO2	2	0
EEFSEC	EEFSEC	2	0
NT5DC1	NT5DC1	-2	0
TOMM70A	TOMM70A	-2	0
IDH3B	IDH3B_tv1	-2	0
ZNF248	ZNF248	-2	0
NFS1	ENSSSCG00000007307	-2	0
C8orf34	C8orf34	-2	0
SNX6	SNX6	-2	0
CALML4	CALML4_tv1	-2	0.02
HSPB8	HSPB8	-2	0
RGCC	RGCC	-2	0
XBP1	XBP1	-2	0
SNRPD3	ENSSSCG00000010058	-2	0
EXOSC9	EXOSC9	-2	0
ZNF569	ZNF569_tv1	-2	0
NAP1L1	NAP1L1	-2	0
GNAI3	GNAI3_tv1	-2	0
NOL3	NOL3_tv1	-2	0
OXNAD1	OXNAD1	-2	0
MTX2	MTX2	-2	0
ALDH7A1	ALDH7A1	-2	0
ACADSB	ACADSB	-2	0
GJA1	GJA1	-2	0
ITGAE	ITGAE	-2	0.02
MSR1	MSR1_tvSR-II	-2	0.01
TP53INP1	TP53INP1	-2	0.05
SYTL3	SYTL3	-2	0.02
OCIAD2	OCIAD2	-2	0

NUP35	NUP35	-2	0
FIBP	FIBP	-2	0
ANO5	ANO5	-2	0
ATP5MC2	ATP5MC2	-2	0
MALSU1	MALSU1	-2	0
RPS26	ENSSSCG00000033697	-2	0
RRAGB	RRAGB	-2	0
EXTL2	EXTL2	-2	0
ODR4	ODR4	-2	0
PPHLN1	PPHLN1	-2	0
FGF7	FGF7	-2	0.02
ARF4	ARF4	-2	0
SACM1L	SACM1L	-2	0
SKIV2L2	SKIV2L2	-2	0
LRPPRC	LRPPRC	-2	0
SUMO4P	SUMO4P	-2	0
DDX25	DDX25	-2	0.01
CAPZA1	CAPZA1	-2	0
GTF3C6	GTF3C6_tv1	-2	0
SKAP2	SKAP2_tv1	-2	0
ATL2	ATL2	-2	0
ANXA7	ANXA7	-2	0
GSTZ1	GSTZ1_tv1	-2	0
SURF1	SURF1_tv1	-2	0
EIF4A1	EIF4A1	-2	0
NCOA4	NCOA4_tv4	-2	0
MRPL11	MRPL11	-2	0
LRR1	LRR1	-2	0
LOC110256034	ENSSSCG00000051634	-2	0
MCFD2	MCFD2	-2	0
TM2D2	TM2D2	-2	0
TXNDC12	TXNDC12_tv1	-2	0
PSMG2	PSMG2	-2	0
ECD	ECD_tv1	-2	0
PLOD2	PLOD2	-2	0
C4orf46	C4orf46	-2	0
NUTF2	NUTF2	-2	0
PTGR1	PTGR1	-2	0
CDC42	CDC42_tv1	-2	0
RPS9	RPS9	-2	0
HIBCH	HIBCH	-2	0
ANKRA2	ANKRA2	-2	0
OGN	OGN_tv1	-2	0
BEX4	BEX4	-2	0
BTF3	BTF3	-2	0
PDCD6	PDCD6	-2	0
RPL30	RPL30	-2	0

PSMB6	PSMB6	-2	0
BLVRB	BLVRB	-2	0
TNNT1	TNNT1	-2	0
HSD11B1	HSD11B1_tv1	-2	0
PRADC1	PRADC1	-2	0
PRKRA	PRKRA	-2	0
PRCP	PRCP	-2	0
ADSL	ADSL	-2	0
LAMTOR5	LAMTOR5_tv1	-2	0
SLC25A40	SLC25A40	-2	0
TMEM33	TMEM33	-2	0
CD24	CD24	-2	0
SEC22C	SEC22C	-2	0
KITLG	KITLG_tvb	-2	0
RPL13A	RPL13A_tv	-2	0
ABHD11	ABHD11_tv1	-2	0.01
DERL3	DERL3_tv1	-2	0
COX17	COX17	-2	0
IGBP1	ENSSSCG00000012382	-2	0
EIF3M	EIF3M	-2	0
FXYD1	FXYD1_tva	-2	0
FAM210A	ENSSSCG00000030438	-2	0
TTK	TTK	-2	0
KBTBD8	KBTBD8	-2	0
MMRN1	MMRN1	-2	0
ZNF558	ZNF558	-2	0
AMD1	AMD1_tv1	-2	0
OXR1	OXR1_tv1	-2	0
MYRFL	MYRFL	-2	0
TSFM	TSFM_tv1	-2	0
CWF19L1	CWF19L1	-2	0
GLRB	GLRB	-2	0.05
RBM48	RBM48	-2	0
MAP3K8	MAP3K8	-2	0.04
CCDC59	CCDC59	-2	0
RMDN2	RMDN2	-2	0
RTRAF	RTRAF	-2	0
DNM1L	DNM1L	-2	0
CDKL3	CDKL3	-2	0.01
EMC7	EMC7	-2	0
LOC102161685	LOC102161685	-2	0
DNAJC15	DNAJC15	-2	0
ENSSSCG00000045295	ENSSSCG00000045295	-2	0
CDC26	CDC26	-2	0
MARCHF5	MARCHF5	-2	0
SLF1	SLF1	-2	0
RNF13	RNF13	-2	0

INSIG2	INSIG2	-2	0
SAR1B	SAR1B_tv2	-2	0
RPL28	RPL28_tv2	-2	0
YEATS4	YEATS4	-2	0
LIPT2	LIPT2	-2	0.03
POP7	POP7	-2	0
ATP6V1C1	ATP6V1C1	-2	0
MED7	MED7	-2	0
ENSSSCG00000049808	ENSSSCG00000049808	-2	0.03
MBD4	MBD4	-2	0.01
SCRN3	SCRN3	-2	0.01
ICT1	ICT1	-2	0
MAP2K6	MAP2K6_tv1	-2	0.02
POLR2E	POLR2E	-2	0
TXNIP	TXNIP_tv1	-2	0
HSD17B11	HSD17B11	-2	0
ABCB1	ENSSSCG00000015390	-2	0.03
GOLT1B	GOLT1B	-2	0
TRIM59	TRIM59_tv1	-2	0
GPR155	GPR155	-2	0
TUBA1A	TUBA1A_tv1	-2	0
LOC110256227	ENSSSCG00000051293	-2	0.01
NDUFS3	NDUFS3	-2	0
SGK1	SGK1_tv1	-2	0
C21orf91	C21orf91	-2	0
PGAM1	PGAM1_tv1	-2	0
OAZ1	OAZ1	-2	0
TCEB2	TCEB2	-2	0
METTL27	METTL27	-2	0.01
LRRC6	LRRC6	-2	0.01
PIGW	PIGW	-2	0
MCRIP2	MCRIP2	-2	0
SUV39H2	SUV39H2_tv1	-2	0
HAGH	HAGH	-2	0
TRAPPC6B	TRAPPC6B	-2	0
CCT3	CCT3	-2	0
MORF4L2	MORF4L2	-2	0
HBP1	HBP1_tv1	-2	0
GLO1	GLO1	-2	0
SSU72L*	ENSSSCG00000012720	-2	0.03
DPT	DPT	-2	0
RABEPK	RABEPK	-2	0
RPRD1A	RPRD1A	-2	0
ETFBKMT	ENSSSCG00000033766	-2	0
FGFR1OP2	FGFR1OP2	-2	0
DDX47	DDX47_tv1	-2	0
PPIL1	PPIL1	-2	0

TSPAN6	TSPAN6_tv1	-2	0
CDC20B	CDC20B	-2	0
PSMB3	PSMB3_tv1	-2	0
XAF1	XAF1	-2	0.01
ARSB	ARSB	-2	0.01
ARFIP1	ARFIP1	-2	0
ARHGEF33	ARHGEF33	-2	0
TYW5	TYW5	-2	0
SPATA5	SPATA5	-2	0
SOD2	SOD2	-2	0
TPI1	TPI1	-2	0.01
HNRNPK	ENSSSCG00000022760	-2	0
NCBP2	NCBP2	-2	0
RWDD3	RWDD3	-2	0
GSR	GSR_tv	-2	0
LOC102160841	ENSSSCG00000043029	-2	0.05
ACP1	ACP1_tv3	-2	0
HACL1	HACL1	-2	0
ZNF300	ZNF300	-2	0
NHEJ1	NHEJ1_tv1	-2	0
PGK1	PGK1	-2	0
SLC35A3	SLC35A3_tv1	-2	0
CNIH4	CNIH4	-2	0
APEX1	APEX1	-2	0.04
SCAMP1	SCAMP1	-2	0
LOC100522319	ENSSSCG00000002850	-2	0
MRPS22	MRPS22	-2	0
AXDND1	AXDND1	-2	0.02
SLC25A21	SLC25A21	-2	0.04
TEX30	TEX30	-2	0
ISCA1	ENSSSCG00000010954	-2	0
HAUS2	HAUS2	-2	0
RPL7A	RPL7A	-2	0
MRPL16	MRPL16	-2	0
PAGE2B	PAGE2B	-2	0.02
SGK3	SGK3_tv1	-2	0
GCDH	GCDH	-2	0
SEMA3C	SEMA3C	-2	0.04
CHODL	CHODL	-2	0
MCCC1	MCCC1_tv1	-2	0
POLI	POLI	-2	0
FAM151B	FAM151B	-2	0
DTWD1	DTWD1	-2	0
SDHAF3	ENSSSCG00000023850	-2	0
DNAJA1	ENSSSCG00000011000	-2	0
UHRF1BP1L	UHRF1BP1L_tv1	-2	0
RUBCN	RUBCN	-2	0

TRDMT1	TRDMT1	-2	0
ZFP82L*	ZFP82L*	-2	0.01
ALG5	ALG5	-2	0
PSMC1	PSMC1	-2	0
SNRPC	SNRPC	-2	0
SLC25A17	SLC25A17	-2	0
HIST1H2AC	ENSSSCG00000045769	-2	0.01
ADK	ADK	-2	0
C1QTNF12	C1QTNF12	-2	0.01
MTX3	MTX3	-2	0
TUBE1	TUBE1	-2	0
FTL	FTL	-2	0
PTGR2	ENSSSCG00000002351	-2	0
C20orf27	C20orf27	-2	0
SRSF7	SRSF7	-2	0
OLA1	OLA1_tv1	-2	0
MRPL2	ENSSSCG00000001658	-2	0
MCTS1	MCTS1	-2	0
ADAM10	ADAM10	-2	0
EXOC5	EXOC5_tv1	-2	0
RPL5	RPL5	-2	0
HEBP2	ENSSSCG00000004150	-2	0
ENSSSCG00000044555	ENSSSCG00000044555	-2	0
SH3BGRL	ENSSSCG00000012452	-2	0
NME2	NME2	-2	0
WDR35	WDR35	-2	0
UBLCP1	UBLCP1	-2	0
CCT6A	CCT6A_tv1	-2	0
FOPNL	FOPNL	-2	0
TMSB4X	ENSSSCG00000033734	-2.1	0
CSDE1	CSDE1	-2.1	0
GNB4	GNB4	-2.1	0
CLIC4	CLIC4	-2.1	0
CTH	CTH_tv1	-2.1	0
DPH5	DPH5	-2.1	0
DPPA3L	DPPA3L	-2.1	0.01
STIM1	STIM1_tv1	-2.1	0.04
CUTC	CUTC_tv1	-2.1	0
PITHD1	PITHD1	-2.1	0
CBR1L2	CBR1L2	-2.1	0
SGTB	SGTB	-2.1	0.02
PSMA4	PSMA4_tv1	-2.1	0
EIF1AY	ENSSSCG00000037509	-2.1	0
TTC1	TTC1	-2.1	0
PDHB	PDHB_tv1	-2.1	0
PIFOL*	ENSSSCG00000035204	-2.1	0.02
LOC106506502	ENSSSCG00000035160	-2.1	0.01

N4BP2L1	N4BP2L1_tv1	-2.1	0
BCAP29	BCAP29	-2.1	0
ZMPSTE24	ZMPSTE24	-2.1	0
NXT2	NXT2_tv1	-2.1	0
NUDT7	NUDT7	-2.1	0
RNASE12	RNASE12	-2.1	0.02
GIMAP5	GIMAP5	-2.1	0.02
PNPT1	PNPT1	-2.1	0
BCAS4	BCAS4_tv2	-2.1	0
C16orf91	C16orf91	-2.1	0
SASS6	SASS6	-2.1	0
CYP11A1	CYP11A1_tv1	-2.1	0
RFC4	RFC4_tv1	-2.1	0
PANK3	PANK3	-2.1	0
SMIM20	ENSSSCG00000040571	-2.1	0
ZFAND6	ZFAND6	-2.1	0
NMD3	NMD3	-2.1	0
MRPL37	MRPL37	-2.1	0
NXNL2	NXNL2	-2.1	0
ENSSSCG00000047395	ENSSSCG00000047395	-2.1	0.05
AASDHPPT	AASDHPPT	-2.1	0
PPP1R1A	PPP1R1A	-2.1	0
ODF2L	ODF2L_tv4	-2.1	0
GAS2	GAS2_tv3	-2.1	0
LOC100517973	ENSSSCG00000040422	-2.1	0
LSM4	LSM4_tv1	-2.1	0
TAF7L	TAF7L	-2.1	0
OSTF1	OSTF1	-2.1	0
LOC102166913	ENSSSCG00000043103	-2.1	0
CEP55	CEP55	-2.1	0
KCTD9	KCTD9	-2.1	0
ASF1A	ASF1A	-2.1	0
NTPCR	NTPCR	-2.1	0
L3HYPDH	L3HYPDH	-2.1	0
RPL15	RPL15	-2.1	0
DRAM2	DRAM2	-2.1	0
RER1	RER1	-2.1	0
WDR76	WDR76	-2.1	0
EIF2S3	EIF2S3	-2.1	0
LYSMD4	ENSSSCG00000030487	-2.1	0
COMMD3	COMMD3	-2.1	0
UBA3	UBA3	-2.1	0
LOC106506526	ENSSSCG00000046259	-2.1	0.05
TMEM186	TMEM186	-2.1	0
ZNF639	ZNF639	-2.1	0
BTK	BTK_tv1	-2.1	0.01
ZNF615	ZNF615_tvX5	-2.1	0.02

EIF1AX	EIF1AX	-2.1	0
PKD2L2	PKD2L2	-2.1	0.01
SLC25A3	SLC25A3	-2.1	0
PSMA8	PSMA8	-2.1	0.03
UBA6	UBA6	-2.1	0
LOC106507930	ENSSSCG00000044691	-2.1	0.03
GET1	GET1	-2.1	0
TAF10	TAF10	-2.1	0
PRPF39	PRPF39	-2.1	0
C5orf15	C5orf15	-2.1	0
SLC4A4	SLC4A4	-2.1	0
LVRN	LVRN	-2.1	0.03
ALKBH7	ALKBH7	-2.1	0
OSBP2	OSBP2	-2.1	0
UBE2A	UBE2A	-2.1	0
POLR2J	ENSSSCG00000007688	-2.1	0
EIF6	EIF6	-2.1	0
MYL2	MYL2	-2.1	0.01
TFPI	TFPI	-2.1	0.02
YOD1	YOD1	-2.1	0
BLOC1S6	BLOC1S6	-2.1	0
CCDC191	CCDC191	-2.1	0
GTDC1	GTDC1	-2.1	0
SCYL2	SCYL2_tvX2	-2.1	0
ZMYM6NB	ZMYM6NB	-2.1	0
ZFAND1	ZFAND1	-2.1	0
RAD17	RAD17	-2.1	0
CFAP97D1	CFAP97D1	-2.1	0.05
CRISPLD1	CRISPLD1	-2.1	0
PMVK	PMVK_tv1	-2.1	0.04
PRDX5	PRDX5	-2.1	0
NUP210L	NUP210L	-2.1	0
TBC1D23	TBC1D23	-2.1	0
KCNK2	KCNK2	-2.1	0
C19orf81	C19orf81	-2.1	0.02
UBE2B	UBE2B	-2.1	0
NDUFAF4	NDUFAF4	-2.1	0
ECHDC1	ECHDC1	-2.1	0
ZNF596	ZNF596	-2.1	0
PCMT1	PCMT1	-2.1	0
TOMM6	TOMM6	-2.1	0
TM2D1	TM2D1	-2.1	0
KATNBL1	KATNBL1	-2.1	0
CNOT7	CNOT7	-2.1	0
DUOXA2	DUOXA2	-2.1	0.03
RMI1	RMI1	-2.1	0
MGAT4A	MGAT4A	-2.1	0

TIMM10	TIMM10	-2.1	0
MED4	MED4	-2.1	0
ZDBF2	ZDBF2	-2.1	0.01
ERI2	ERI2_tv1	-2.1	0.01
AP5M1	AP5M1_tv1	-2.1	0.02
TAF11	ENSSSCG00000001533	-2.1	0
MIGA1	MIGA1	-2.1	0
RPL18	ENSSSCG00000025928	-2.1	0
PCCA	PCCA_tv1	-2.1	0
EEF1B2	EEF1B2	-2.1	0
ARMT1	ARMT1_tv1	-2.1	0
GPN1	GPN1	-2.1	0
NUF2	NUF2	-2.1	0
CST9L	CST9L	-2.1	0
COPS6	COPS6	-2.1	0
CCNJ	CCNJ	-2.1	0.03
ORC4	ORC4	-2.1	0
CASC1	CASC1	-2.1	0
SLC7A11	SLC7A11	-2.1	0
EMC4	EMC4	-2.1	0
UBA5	UBA5_tv1	-2.1	0
C3orf38	C3orf38	-2.1	0
TRIM13	TRIM13	-2.1	0
GNPDA2	GNPDA2	-2.1	0
SCPEP1	SCPEP1	-2.1	0
PCNA	PCNA	-2.1	0
PSMB4	PSMB4	-2.1	0
EIF2B2	EIF2B2	-2.1	0
RPL21	RPL21	-2.1	0
MRPL24	MRPL24	-2.1	0
UNK57*	UNK57	-2.1	0.03
CENPQ	CENPQ	-2.1	0
GEN1	GEN1	-2.1	0
GLA	GLA	-2.1	0
CNIH1	CNIH1	-2.1	0
RBBP4	RBBP4	-2.1	0
ZNF471	ZNF471	-2.1	0.03
APOLD1	APOLD1_tv2	-2.1	0
LMBRD1	LMBRD1	-2.1	0
UNK156*	ENSSSCG00000048074	-2.1	0
NDUFA8	NDUFA8	-2.1	0
ENSSSCG00000048556	ENSSSCG00000048556	-2.1	0
BTF3L4	BTF3L4	-2.1	0
NDUFA4	ENSSSCG00000024313	-2.1	0
RGS5	RGS5	-2.1	0
FRG1	ENSSSCG00000006996	-2.1	0
MRPL55	MRPL55_tv1	-2.1	0

GTF2A2	GTF2A2	-2.1	0
STRAP	STRAP	-2.1	0
RPL10	RPL10_tv1	-2.1	0
CLEC2D	CLEC2D_tv1	-2.1	0
PSME2	PSME2_tv1	-2.1	0
DARS	DARS	-2.1	0
BCL2A1	BCL2A1_tv1	-2.1	0.01
CCNYL1	CCNYL1	-2.1	0
ZNF410	ZNF410_tv2	-2.1	0
UCHL5	UCHL5_tv1	-2.1	0
MYO5A	MYO5A	-2.1	0
SEC22B	SEC22B	-2.1	0
SLC25A51	SLC25A51	-2.1	0
MRPL18	MRPL18	-2.1	0
ANAPC7	ANAPC7	-2.1	0
HTD2	HTD2	-2.1	0.02
RPL35	RPL35	-2.1	0
TAX1BP3	TAX1BP3	-2.1	0
RPL21Ps2*	ENSSSCG00000011688	-2.1	0
HMGCL	HMGCL_tv1	-2.1	0
RIDA	RIDA	-2.1	0
EIF3H	EIF3H	-2.1	0
KRBOX4	ENSSSCG00000049838	-2.1	0.04
POLB	POLB	-2.1	0
MGARP	MGARP	-2.1	0
SFT2D1	SFT2D1	-2.1	0
TMEM50A	TMEM50A	-2.1	0
LOC102160564	ENSSSCG00000041434	-2.1	0
DCN	DCN	-2.1	0
POLR2I	POLR2I	-2.1	0
MUT	MUT	-2.1	0
HNMT	HNMT_tv1	-2.1	0
CCNE2	CCNE2	-2.1	0
DPYS	DPYS	-2.1	0.03
DYNLL1	DYNLL1	-2.1	0
SBDS	SBDS	-2.1	0
CHCHD1	CHCHD1	-2.1	0
MAP6D1	MAP6D1	-2.1	0
COA6	ENSSSCG00000010161	-2.1	0
TRIM23	TRIM23_tv1	-2.1	0
ZFP37	ENSSSCG00000005475	-2.1	0
SEMA3A	SEMA3A	-2.1	0
AK7	AK7	-2.1	0
ZNF709L11*	ZNF709L11*	-2.1	0.03
CASP4	CASP4_tva	-2.1	0
RPL12	RPL12	-2.1	0
NDUFB3	NDUFB3	-2.1	0

CLPX	CLPX_tv	-2.1	0
TSPAN31	TSPAN31	-2.1	0
METTL9	METTL9	-2.1	0
LIPA	LIPA_tv2	-2.1	0
CD3G	CD3G_tv1	-2.1	0
PRDX6	PRDX6	-2.1	0
NDUFA7	ENSSSCG00000041220	-2.1	0
TRAPPC4	TRAPPC4	-2.2	0
PGLYRP2	PGLYRP2	-2.2	0.05
ACTR3	ACTR3_tv1	-2.2	0
ARPC5L	ARPC5L	-2.2	0
KIAA1143	KIAA1143	-2.2	0
TMEM59	TMEM59	-2.2	0
RPLP2	RPLP2	-2.2	0
C17orf75	C17orf75	-2.2	0
ENSSSCG00000043997	ENSSSCG00000043997	-2.2	0
ENSSSCG00000041012	ENSSSCG00000041012	-2.2	0.03
C2orf76	C2orf76	-2.2	0
SULT1C3	ENSSSCG00000028691	-2.2	0
CCNDBP1	CCNDBP1	-2.2	0
IMPA1	IMPA1	-2.2	0
THOC5	THOC5	-2.2	0
IFI44	IFI44	-2.2	0
EFR3A	EFR3A_tv1	-2.2	0
SGCE	SGCE	-2.2	0
RRAS2	ENSSSCG00000033786	-2.2	0
POLM	ENSSSCG00000044483	-2.2	0
NRGN	NRGN	-2.2	0.01
NDUFS2	NDUFS2	-2.2	0
MIS18A	MIS18A	-2.2	0
NOP16	NOP16_tv3	-2.2	0
ENSSSCG00000049380	ENSSSCG00000049380	-2.2	0
SLC30A6	SLC30A6	-2.2	0
GPR34	GPR34_tv1	-2.2	0.01
C3orf14	C3orf14	-2.2	0.03
WSB1	WSB1	-2.2	0
MDH1	MDH1_tv2	-2.2	0
CCT2	CCT2_tv1	-2.2	0
MAP3K7	MAP3K7_tvb	-2.2	0
RPL34	RPL34	-2.2	0
MTERF2	MTERF2	-2.2	0
RPL7L1	RPL7L1	-2.2	0
MPC2	MPC2	-2.2	0
TMEM229A	ENSSSCG00000044009	-2.2	0
SUCLA2	SUCLA2_tv1	-2.2	0
S100A6	S100A6	-2.2	0
DEPDC1	DEPDC1_tv1	-2.2	0

PPDPF	ENSSSCG00000040118	-2.2	0.04
GHRL	GHRL	-2.2	0
ENSSSCG00000044123	ENSSSCG00000044123	-2.2	0.02
FAM216A	FAM216A	-2.2	0.01
ALB	ALB_tv	-2.2	0
TMBIM4	TMBIM4_tv2	-2.2	0
IFRD2	IFRD2	-2.2	0
SRXN1	SRXN1	-2.2	0
GPBP1	GPBP1_tv1	-2.2	0
PSTK	PSTK	-2.2	0
PLBD1	PLBD1	-2.2	0
RPL23	RPL23	-2.2	0
PSMD10	PSMD10	-2.2	0
PSMB7	PSMB7	-2.2	0
CDK1	CDK1_tv1	-2.2	0
SLC25A13	SLC25A13_tv2	-2.2	0
DNAJC12	DNAJC12	-2.2	0
SNX5	ENSSSCG00000007088	-2.2	0
ZBTB6	ZBTB6	-2.2	0
SENP8	SENP8	-2.2	0
C4BPAL*	C4BPAL*	-2.2	0
UOX	UOX	-2.2	0
FBXO22	FBXO22	-2.2	0
CHUK	CHUK	-2.2	0
SULT2A1	SULT2A1	-2.2	0
ARPC2	ARPC2	-2.2	0
TRIM33	TRIM33_tv	-2.2	0
PDCD4	PDCD4	-2.2	0
PSMB2	PSMB2_tv1	-2.2	0
SEC22A	SEC22A	-2.2	0
UBXN8	UBXN8	-2.2	0
LIN9	LIN9_tv1	-2.2	0
COMMD10	COMMD10	-2.2	0
CASP3	CASP3_tvb	-2.2	0
TMEM106C	TMEM106C	-2.2	0
GABARAPL2	GABARAPL2	-2.2	0
lncRNA	lncRNA	-2.2	0.02
TM4SF18	TM4SF18	-2.2	0
EIF5A2	EIF5A2	-2.2	0
SH3BGRL2	SH3BGRL2	-2.2	0
SRP14	SRP14	-2.2	0
CHURC1	CHURC1	-2.2	0
PSPN	PSPN	-2.2	0.04
C18orf32	ENSSSCG00000034415	-2.2	0
SMNDC1	SMNDC1	-2.2	0
SNRPD2	SNRPD2	-2.2	0
SIAE	SIAE_tv1	-2.2	0

H1-3	H1-3	-2.2	0
PSMA3	PSMA3_tv1	-2.2	0
RPS27A	RPS27A	-2.2	0
LOC102165926	ENSSSCG00000044342	-2.2	0.04
FKBP3	FKBP3_tv1	-2.2	0
AAGAB	AAGAB_tv1	-2.2	0
PEBP1	ENSSSCG00000009851	-2.2	0
COMMD8	COMMD8	-2.2	0
C4orf3	ENSSSCG00000031773	-2.2	0
SAT1	SAT1_tv1	-2.2	0
AP1S3_tv1	AP1S3_tv1	-2.2	0.04
CSE1L	CSE1L_tv	-2.2	0
SPRYD4	SPRYD4	-2.2	0
TRHDE	TRHDE	-2.2	0
CHCHD5	CHCHD5	-2.2	0
MFN1	MFN1	-2.2	0
BTG3	BTG3_tv2	-2.2	0
RPS4X	RPS4X	-2.2	0
CGRRF1	CGRRF1	-2.2	0
ZNF22	ZNF22	-2.2	0
MAG	MAG	-2.2	0.01
MRPS31	MRPS31	-2.2	0
MTFR1	MTFR1	-2.2	0
TRMT61B	TRMT61B	-2.2	0
RFC3	RFC3	-2.2	0
HGD	HGD	-2.2	0.05
TUBD1	TUBD1	-2.2	0
RPS3A	RPS3A	-2.2	0
CAV2	CAV2	-2.2	0
BOLA3	BOLA3	-2.2	0
TAF1D	TAF1D	-2.2	0
VBP1	VBP1	-2.2	0
LOC100155572	ENSSSCG00000002045	-2.2	0
TSTD3	ENSSSCG00000038757	-2.2	0
RPSA	RPSA	-2.2	0
COL12A1	COL12A1	-2.2	0
TYW3	TYW3	-2.2	0
LOC106507125	ENSSSCG00000045495	-2.2	0
SLC30A9	SLC30A9	-2.2	0
RPL38	RPL38	-2.2	0
DTD2	DTD2	-2.2	0
ATP6V1D	ATP6V1D	-2.2	0
CCT8	CCT8	-2.2	0
RHOH	RHOH_tv4	-2.2	0.05
COQ7	COQ7	-2.2	0
ZBTB8OS	ZBTB8OS	-2.2	0
ACAT1	ACAT1_tv	-2.2	0

TRIQK	TRIQK	-2.2	0
PLK2	PLK2	-2.2	0.01
CST15	ENSSSCG00000007123	-2.2	0
NDUFB6	NDUFB6	-2.2	0
HSD17B4	HSD17B4_tv2	-2.2	0
MTHFD2	MTHFD2_tv1	-2.2	0
DSTN	DSTN_tv1	-2.2	0
BZW1	BZW1	-2.2	0
MRPL27	MRPL27	-2.2	0
BST2	BST2_tv1	-2.2	0
DPH3P1	DPH3P1	-2.2	0
UBE2T	UBE2T	-2.2	0.05
PGM3	PGM3	-2.2	0
GSTA1	GSTA1_tv1	-2.2	0
H3F3B	H3F3B	-2.2	0
EIF3E	EIF3E	-2.2	0
RAB2A	RAB2A_tv1	-2.2	0
ENSSSCG00000000359	ENSSSCG00000000359	-2.2	0
MRPL47	MRPL47	-2.2	0
BDH2	BDH2	-2.2	0
DPY30	DPY30	-2.2	0
FUNDC1	FUNDC1	-2.2	0
NAE1	NAE1	-2.2	0
PAGE2BL*	ENSSSCG00000049886	-2.2	0.03
ENSSSCG00000045922	ENSSSCG00000045922	-2.2	0
CEP85L	CEP85L	-2.2	0
COX15	COX15	-2.2	0
SOCS5	SOCS5	-2.3	0
ZBTB26	ZBTB26	-2.3	0
HSPA14	HSPA14_tv1	-2.3	0
NUDT12	NUDT12	-2.3	0
TATDN3	TATDN3	-2.3	0
CYP39A1	CYP39A1_tv1	-2.3	0
COX11	COX11_tv1	-2.3	0
PARK7	PARK7	-2.3	0
POLR2K	POLR2K	-2.3	0
RPL22	RPL22	-2.3	0
NIPA2	NIPA2_tv1	-2.3	0.01
CSNK1A1L	CSNK1A1L_tv1	-2.3	0.05
PTTG1	ENSSSCG00000017032	-2.3	0
AIF1	AIF1_tv3	-2.3	0
CLDND1	CLDND1	-2.3	0
SLC35F4	SLC35F4	-2.3	0
CYP51A1	CYP51A1_tv1	-2.3	0
C18orf21	C18orf21	-2.3	0
ZNF706	ZNF706	-2.3	0
ATP6V1F	ATP6V1F	-2.3	0

RPS17	RPS17	-2.3	0
RTCA	RTCA	-2.3	0
CFAP20	CFAP20	-2.3	0
GYG1	GYG1_tv2	-2.3	0.02
DUT	DUT	-2.3	0
HSD17B8	HSD17B8	-2.3	0
MZT1	MZT1	-2.3	0
NMRK1	NMRK1	-2.3	0
TDG	TDG	-2.3	0
MRPL17	MRPL17	-2.3	0
MYNN	MYNN	-2.3	0
CMPK1	CMPK1	-2.3	0
ENSSSCG00000045093	ENSSSCG00000045093	-2.3	0.01
PSMD14	PSMD14	-2.3	0
TRAV18	ENSSSCG00000040548	-2.3	0.03
RAP1GAP	RAP1GAP	-2.3	0
PSPH	PSPH	-2.3	0.04
RCHY1	RCHY1	-2.3	0
CYP4B1	CYP4B1	-2.3	0
ENY2	ENY2_tv1	-2.3	0
RPL36AL	RPL36AL_tv	-2.3	0
CLN5	ENSSSCG00000031288	-2.3	0
ROGDI	ROGDI	-2.3	0
TBC1D15	TBC1D15	-2.3	0
BLVRA	BLVRA_tv	-2.3	0
IAH1	IAH1	-2.3	0
EXD1	EXD1	-2.3	0
ANXA1	ANXA1_tv1	-2.3	0
KLHL7	KLHL7_tv1	-2.3	0
ENSSSCG00000042997	ENSSSCG00000042997	-2.3	0
TLR1	TLR1	-2.3	0
SAXO2	SAXO2	-2.3	0.04
THAP1	THAP1	-2.3	0
SOAT1	SOAT1_tv1	-2.3	0
DERA	DERA	-2.3	0
KYAT3	KYAT3	-2.3	0
PFDN4	PFDN4	-2.3	0
TMEM183A	TMEM183A	-2.3	0
FDX1	FDX1	-2.3	0
ENSSSCG00000041281	ENSSSCG00000041281	-2.3	0
PDHX	PDHX_tv1	-2.3	0
ACTR10	ACTR10	-2.3	0
MAGOHB	MAGOHB	-2.3	0
ESCO2	ENSSSCG00000009670	-2.3	0
LOC106510665	ENSSSCG00000051754	-2.3	0
METAP2	METAP2	-2.3	0
CKS2	CKS2	-2.3	0

RPS13	RPS13	-2.3	0
SLC35A1	SLC35A1	-2.3	0
EXOSC8	EXOSC8	-2.3	0
CTSC	CTSC_tv1	-2.3	0
STK17B	STK17B	-2.3	0
GEMIN2	GEMIN2	-2.3	0
SPCS1	SPCS1	-2.3	0
TMEM147	TMEM147	-2.3	0
POLR2G	POLR2G	-2.3	0
PCCB	PCCB	-2.3	0
AMZ2	AMZ2	-2.3	0
PPT1	PPT1	-2.3	0
ANAPC4	ANAPC4	-2.3	0.03
ITM2B	ITM2B	-2.3	0
CCT4	CCT4	-2.3	0
RBBP7	RBBP7	-2.3	0
CCDC32	CCDC32	-2.3	0
EBAG9	EBAG9_tv2	-2.3	0.01
TFB2M	TFB2M	-2.3	0
NUDT15	NUDT15	-2.3	0
MRPL1	MRPL1	-2.3	0
CASP6	CASP6_tva	-2.3	0
TMEM144	TMEM144	-2.3	0
ERO1B	ERO1B	-2.3	0
AMN1	AMN1	-2.3	0
FAHD2A	FAHD2A	-2.3	0
ENSSSCG00000051580	ENSSSCG00000051580	-2.3	0
CNPY2	ENSSSCG00000000394	-2.3	0
GLOD4	GLOD4_tv1	-2.3	0
NDUFA5	NDUFA5	-2.3	0
SMIM15	SMIM15	-2.3	0
PSMA1	PSMA1_tv2	-2.3	0
GRIK1	GRIK1	-2.3	0
GIN1	GIN1	-2.3	0
PECR	PECR	-2.3	0
MAP1LC3B	MAP1LC3B	-2.3	0
ATP6V1E1	ATP6V1E1	-2.3	0
TMEM14A	TMEM14A	-2.3	0
ERH	ERH	-2.3	0
LAGE3	ENSSSCG00000032959	-2.3	0
NCF4	NCF4_tv1	-2.3	0
SMU1	SMU1	-2.3	0
TANK	TANK_tv1	-2.3	0
LOC106505126	ENSSSCG00000051707	-2.3	0
TPRKB	TPRKB	-2.3	0
C12orf29	C12orf29	-2.3	0
CP	CP	-2.3	0

TMEM100	TMEM100	-2.3	0
ELMOD2	ELMOD2_tv1	-2.3	0
FCF1	FCF1	-2.3	0
ATP6V1G1	ATP6V1G1	-2.3	0
SMIM7	SMIM7	-2.3	0
LSM8	LSM8	-2.3	0
RAMAC	ENSSSCG00000038630	-2.3	0
FAM133A	FAM133A	-2.3	0
ESD	ESD	-2.3	0
SLITRK6	SLITRK6	-2.3	0
DBI	DBI	-2.3	0
CYP19A3*	CYP19A3*	-2.3	0
UAP1	UAP1	-2.3	0
LYRM1	LYRM1	-2.3	0
PPP2R3C	PPP2R3C	-2.3	0
TST	TST_tv1	-2.4	0
FECH	FECH	-2.4	0
TMEM128	TMEM128	-2.4	0
IPMK	IPMK	-2.4	0
HSPA13	HSPA13	-2.4	0
NPTN	NPTN_tva	-2.4	0.03
ATP1B3	ATP1B3	-2.4	0
SLC30A8	SLC30A8	-2.4	0
ZNF345	ZNF345_tv1	-2.4	0.01
SFXN3	SFXN3	-2.4	0
C6orf120	C6orf120	-2.4	0
RPL32	RPL32_tv1	-2.4	0
ADAMTS1	ADAMTS1	-2.4	0.02
PCBD1	PCBD1	-2.4	0
MSRB1	MSRB1	-2.4	0
CCNC	CCNC	-2.4	0
TSPAN13	TSPAN13	-2.4	0
SLC6A2	SLC6A2	-2.4	0.04
JTB	ENSSSCG00000006559	-2.4	0
TLR6	TLR6	-2.4	0.01
PSMD5	PSMD5_tv	-2.4	0
MT-CO1	MT-CO1	-2.4	0
ADH5	ADH5	-2.4	0
FAM200B	ENSSSCG00000041490	-2.4	0
CBR3	CBR3	-2.4	0
INO80C	INO80C	-2.4	0
H2AZ2	H2AZ2	-2.4	0
TMSB10	TMSB10	-2.4	0
POPDC3	POPDC3	-2.4	0.01
C11orf1	C11orf1	-2.4	0
VPS26A	VPS26A_tv1	-2.4	0
ANAPC16	ANAPC16_tv2	-2.4	0.01

C5orf30	C5orf30	-2.4	0
SLC39A8	SLC39A8_tv2	-2.4	0
SPATA24	SPATA24	-2.4	0
IFIH1	IFIH1	-2.4	0
OGFRL1	OGFRL1	-2.4	0.01
SPCS3	SPCS3	-2.4	0
GGTA1	GGTA1_tv1	-2.4	0
TOMM5L*	ENSSSCG00000043481	-2.4	0.02
PPFIA2	PPFIA2	-2.4	0
NPL	NPL	-2.4	0
LOC100627892	LOC100627892_tv1	-2.4	0
HSCB	HSCB	-2.4	0
ASNA1	ASNA1	-2.4	0
HGF	HGF_tv1	-2.4	0
ATP5PB	ATP5PB	-2.4	0
RPS25	ENSSSCG00000036296	-2.4	0
MYL7	MYL7	-2.4	0
WDR61	WDR61_tv2	-2.4	0
PDCD1LG2	PDCD1LG2	-2.4	0
TIMM8B	TIMM8B	-2.4	0
GADL1	GADL1	-2.4	0.01
CDKN2AIPNL	CDKN2AIPNL	-2.4	0
"MARCH7"	MARCH7_tvX1	-2.4	0
C11orf58	C11orf58	-2.4	0
LYVE1	LYVE1	-2.4	0
ENSSSCG00000002459	ENSSSCG00000002459	-2.4	0
SNX3	SNX3	-2.4	0
TIMM9	TIMM9_tv1	-2.4	0
PDIK1L	PDIK1L	-2.4	0
PCDH15	PCDH15_tvP	-2.4	0
QRSL1	QRSL1	-2.4	0
LncRNA	LncRNA	-2.4	0
SNX4	SNX4	-2.4	0
TXNL1	TXNL1	-2.4	0
APIP	APIP_tv1	-2.4	0
TMEM242	TMEM242	-2.4	0
COPS9	ENSSSCG00000020790	-2.4	0
MIS12	MIS12_tv3	-2.4	0
TRMT5	TRMT5	-2.4	0
BOLA2	BOLA2	-2.4	0
LUZP2	LUZP2	-2.4	0
FNIP2	FNIP2	-2.4	0
C1QBP	C1QBP	-2.4	0
UNK18*	UNK18	-2.4	0.01
CCDC110	ENSSSCG00000015795	-2.4	0.01
C4BPA	C4BPA_tv1	-2.4	0.03
MRPL15	MRPL15	-2.4	0

RPL14	ENSSSCG00000011272	-2.4	0
TMEM69	TMEM69	-2.4	0
RBX1	ENSSSCG00000034230	-2.4	0
PSMB1	PSMB1	-2.4	0
ACTR6	ACTR6	-2.4	0
MPC1	MPC1	-2.4	0
PBK	PBK	-2.4	0
ZNF32	ZNF32	-2.4	0
NDUFB8	NDUFB8	-2.4	0
IFIT1	IFIT1_tv1	-2.4	0
RABL3	RABL3	-2.4	0
COMMD2	COMMD2	-2.4	0
SNRPG	SNRPG	-2.4	0
NDUFS1	NDUFS1	-2.4	0
NDUFA13	NDUFA13	-2.4	0
SCCPDH	SCCPDH	-2.4	0
H4C9	H4C9	-2.4	0
STAR	STAR	-2.4	0
RPS2Ps*	ENSSSCG00000017377	-2.4	0
LOC106504207	ENSSSCG00000041986	-2.4	0.04
DNAJC28	DNAJC28	-2.4	0
VPS29	VPS29	-2.4	0
UBL5	UBL5	-2.4	0
PAIP1	PAIP1	-2.4	0
KIAA2012	KIAA2012	-2.4	0
LCA5	LCA5_tv1	-2.4	0
PTP4A1	PTP4A1	-2.4	0
RPS15	RPS15	-2.4	0
FABP3	FABP3	-2.4	0
TMEM61	TMEM61	-2.5	0
FOXRED1	FOXRED1_tv1	-2.5	0.02
UBE2N	UBE2N	-2.5	0
MRPS18C	MRPS18C	-2.5	0
RPS4XPs*	ENSSSCG00000022191	-2.5	0
DMAC2L	DMAC2L	-2.5	0
CTSS	CTSS_tv1	-2.5	0
NIT2	NIT2	-2.5	0
SAMD9_tv2	SAMD9_tv2	-2.5	0
CST6	CST6	-2.5	0
FAM96A	FAM96A_tv1	-2.5	0
RPS28	RPS28	-2.5	0
OSGEPL1	OSGEPL1	-2.5	0
NAMPT	NAMPT	-2.5	0
NSUN3	NSUN3	-2.5	0
MAL2	MAL2	-2.5	0
PPP1CC	PPP1CC_tv1	-2.5	0
FAM162A	FAM162A	-2.5	0

NDUFB4	NDUFB4_tv1	-2.5	0
ZNF529	ZNF529_tv1	-2.5	0
EIF4A2	EIF4A2	-2.5	0
RMDN1	RMDN1	-2.5	0
COX7C	COX7C	-2.5	0
NCAM2	NCAM2_tv1	-2.5	0
MT-ND5	MT-ND5	-2.5	0
RNF181	RNF181	-2.5	0
LSM5	ENSSSCG00000026064	-2.5	0
UQCRFS1	UQCRFS1	-2.5	0
LAPTM4A	LAPTM4A	-2.5	0
ASTE1	ASTE1	-2.5	0
CHEK2	CHEK2	-2.5	0
FYTTD1	FYTTD1	-2.5	0
TIMM23	TIMM23_tv1	-2.5	0
TPMT	TPMT	-2.5	0.01
THAP6	THAP6	-2.5	0
TOB1	TOB1_tv1	-2.5	0
KRT10	KRT10	-2.5	0
LOC100626258	ENSSSCG00000044256	-2.5	0
ATOX1	ATOX1	-2.5	0
NADK2	NADK2	-2.5	0
RPL23A	RPL23A	-2.5	0
ATMIN	ATMIN_tv1	-2.5	0.02
ITPA	ITPA	-2.5	0
ABITRAM	ABITRAM	-2.5	0
MRPS25	MRPS25	-2.5	0
ME1	ME1	-2.5	0
GPN3	GPN3	-2.5	0
HINT3	HINT3	-2.5	0
ZNF711	ZNF711	-2.5	0
VDAC2	VDAC2	-2.5	0
GPR171	GPR171	-2.5	0.03
METTL18	METTL18	-2.5	0
DRG1	DRG1	-2.5	0
CRYZ	CRYZ	-2.5	0
JADE3	JADE3	-2.5	0
TMEM243	TMEM243	-2.5	0
B2M	B2M	-2.5	0
CD36	CD36_tvX1	-2.5	0.01
GINM1	GINM1	-2.5	0
HSDL2	HSDL2	-2.5	0
ZNF658	ZNF658	-2.5	0
CCK	CCK_tv1	-2.5	0
UFM1	UFM1	-2.5	0
CTNNB1	CTNNB1_tv1	-2.5	0
ENSSSCG00000037067	ENSSSCG00000037067	-2.5	0.01

TMEM126B	TMEM126B	-2.5	0
HSD17B12	HSD17B12	-2.5	0
SUMO2	SUMO2	-2.5	0
KLHL41	KLHL41	-2.5	0
ZNF684L	ZNF684L	-2.5	0
VTA1	VTA1	-2.5	0
ZNF501	ZNF501_tv1	-2.5	0
EIF2S1	EIF2S1	-2.5	0
EIF3I	EIF3I	-2.5	0
MBIP	MBIP	-2.5	0
C9	C9	-2.5	0.01
COQ3	COQ3_tv1	-2.5	0
GSTO1	GSTO1	-2.5	0
TMEM230Ps*	ENSSSCG00000035546	-2.5	0
PBLD	PBLD	-2.5	0
CFAP43	CFAP43	-2.5	0
RBM7	ENSSSCG00000027905	-2.5	0
TMEM256	ENSSSCG00000037071	-2.5	0
SNX16	SNX16	-2.5	0
PSMC6	PSMC6	-2.5	0
TMEM263	TMEM263	-2.5	0
PRDX2	PRDX2	-2.5	0
SFR1	SFR1	-2.5	0
CAMTA1	CAMTA1_tv2	-2.5	0
DNAJA1	DNAJA1	-2.5	0
UBQLN1	UBQLN1_tv1	-2.5	0
CNEP1R1	CNEP1R1_tv2	-2.5	0
MRPL34	MRPL34	-2.5	0
BDKRB1	BDKRB1_tv	-2.5	0.04
MRPL46	MRPL46	-2.5	0
PDK4	PDK4	-2.6	0
LncRNA	LncRNA	-2.6	0
OSTC	OSTC	-2.6	0
ATP5F1BPs*	ATP5F1BPs*	-2.6	0
ENSSSCG00000049265	ENSSSCG00000049265	-2.6	0
GNG10	GNG10_tv1	-2.6	0
HSD11B1L	HSD11B1L	-2.6	0
MRPL45	MRPL45	-2.6	0
LOC110256941	ENSSSCG00000050742	-2.6	0.01
PCBD2	ENSSSCG00000026229	-2.6	0
MICOS13	MICOS13	-2.6	0
MGAT4C	MGAT4C	-2.6	0
TOMM22	ENSSSCG00000000097	-2.6	0
MRO	MRO	-2.6	0
FBXO8	FBXO8	-2.6	0
CDKL2	CDKL2	-2.6	0
STMN1	STMN1	-2.6	0

NUBPL	NUBPL	-2.6	0
VDAC3	VDAC3	-2.6	0
ARHGAP15	ARHGAP15_tv1	-2.6	0
NOP10	NOP10	-2.6	0
XKR9	XKR9	-2.6	0.02
RNFT1	RNFT1	-2.6	0
VAMP7	VAMP7	-2.6	0
CCNG2	CCNG2	-2.6	0
PRORSD1	ENSSSCG00000037399	-2.6	0
CLECL1	CLECL1_tvX1	-2.6	0
CYCS	CYCS	-2.6	0
NUDT2	NUDT2	-2.6	0
PNO1	PNO1	-2.6	0
FAM227B	FAM227B	-2.6	0
ZNF277	ZNF277	-2.6	0
THOC7	THOC7	-2.6	0
SLC16A6	SLC16A6	-2.6	0.02
RPS18Ps*	ENSSSCG00000008767	-2.6	0
TXNDC9	TXNDC9	-2.6	0
DPM1	DPM1	-2.6	0
CMSS1	CMSS1_tv1	-2.6	0
NDUFA6	NDUFA6	-2.6	0
MPZL3	MPZL3	-2.6	0.01
IMMP1L	IMMP1L	-2.6	0
SDHC	SDHC_tv1	-2.6	0
PCLAF	PCLAF_tv1	-2.6	0
RRM2B	RRM2B_tv1	-2.6	0
ENSSSCG00000044841	ENSSSCG00000044841	-2.6	0
NDUFAF8	NDUFAF8	-2.6	0
GPR156	GPR156	-2.6	0
CETN2	ENSSSCG00000012757	-2.6	0
SUCLG2	SUCLG2_tv2	-2.6	0
PCGF6	PCGF6_tv1	-2.6	0
UBE2L3	UBE2L3_tv1	-2.6	0
SEC61G	SEC61G_tv1	-2.6	0
MIR9810	MIR9810	-2.6	0
SLC44A5	SLC44A5_tv2	-2.6	0
THAP5	THAP5	-2.6	0
EIF2B1Ps*	ENSSSCG00000016241	-2.6	0
CISD2	CISD2	-2.6	0
IL20RB	IL20RB	-2.6	0.01
ND6	ND6	-2.6	0
PCNAPs*	ENSSSCG00000031854	-2.6	0
SELENOP	SELENOP	-2.6	0
LDHA	LDHA	-2.6	0
BORCS7	BORCS7	-2.6	0
ENSSSCG00000043846	ENSSSCG00000043846	-2.6	0

SNRPE	SNRPE	-2.6	0
CSRNP3	CSRNP3_tv1	-2.6	0
COQ5	COQ5	-2.6	0
MAGOH	MAGOH	-2.6	0
MAT2B	MAT2B	-2.6	0
RPL18Ps*	ENSSSCG00000031482	-2.6	0
ERGIC2	ERGIC2	-2.6	0
SMPDL3A	SMPDL3A	-2.6	0.01
YPEL5	YPEL5	-2.6	0
SNRPB2	ENSSSCG00000037602	-2.6	0
AIG1	AIG1_tv1	-2.6	0
ISCA1Ps*	ENSSSCG00000017077	-2.6	0
GTPBP10	GTPBP10	-2.6	0
PYURF	PYURF	-2.6	0
ATP5F1E	ENSSSCG00000007524	-2.6	0
CMC2	CMC2	-2.6	0
RPL27	RPL27	-2.6	0
ACADM	ACADM_tv1	-2.6	0
MGST1	MGST1_tv4	-2.6	0
SUMO1	SUMO1	-2.6	0
GLRX2	GLRX2_tv2	-2.6	0
S100A2	S100A2_tv	-2.6	0
MRPL33	MRPL33	-2.6	0
GGNBP1	ENSSSCG00000045946	-2.6	0
NDUFA12	NDUFA12	-2.6	0
HYAL4	HYAL4	-2.6	0.03
RBBP8	RBBP8_tv1	-2.6	0
NDUFB2	NDUFB2	-2.6	0
UBD	UBD	-2.6	0.02
SRP9	SRP9	-2.6	0
GSKIP	GSKIP	-2.7	0
CHAC1	CHAC1	-2.7	0
PSMD12	PSMD12_tv1	-2.7	0.01
PPA1	PPA1_tv1	-2.7	0
LOC100514211	LOC100514211	-2.7	0
ELOF1	ENSSSCG00000013613	-2.7	0
MSMB	MSMB	-2.7	0
PTGER2	PTGER2	-2.7	0.01
RPL9L*	ENSSSCG00000012427	-2.7	0
RPL36A-HNRNPH2	RPL36A-HNRNPH2	-2.7	0
PNRC2	PNRC2	-2.7	0
HINT1	HINT1	-2.7	0
KRT8	KRT8_tv2	-2.7	0
ENSSSCG00000042457	ENSSSCG00000042457	-2.7	0
ANAPC13	ANAPC13	-2.7	0
CYTIP	CYTIP_tv1	-2.7	0.01
POP5	POP5	-2.7	0

TDH	ENSSSCG00000021767	-2.7	0
ARPC5	ARPC5	-2.7	0
LINS1	LINS1	-2.7	0
UBXN1	UBXN1_tvX2	-2.7	0
RPS20	RPS20	-2.7	0
PPP1R3C	PPP1R3C	-2.7	0
OXSM	OXSM_tv1	-2.7	0
IFI44L	IFI44L_tvX1	-2.7	0
LSM3	LSM3	-2.7	0
BOLA1	BOLA1	-2.7	0
RAD51C	RAD51C_tv1	-2.7	0
GHITM	GHITM_tv1	-2.7	0
SAR1A	SAR1A	-2.7	0
LGALSL	LGALSL	-2.7	0
GPD1L	GPD1L	-2.7	0
MRPS36	MRPS36	-2.7	0
ZNF91	ZNF91_tvX3	-2.7	0.02
ZNF709L2*	ZNF709L2*	-2.7	0
MICOS10	ENSSSCG00000028414	-2.7	0
ANKRD37	ANKRD37	-2.7	0
AIMP1	AIMP1_tv3	-2.7	0
LOC102159626	ENSSSCG00000042452	-2.7	0
RPL7	RPL7	-2.7	0
CHCHD2	CHCHD2	-2.7	0
RWDD4	RWDD4	-2.7	0
MFSD8	MFSD8	-2.7	0
ARMC1	ARMC1	-2.7	0
ACTR2	ACTR2_tv2	-2.7	0
EIF2S3Y	EIF2S3Y	-2.7	0
METTL23	METTL23	-2.7	0
SUCLG1	SUCLG1	-2.7	0
NIPSNAP3A	NIPSNAP3A	-2.7	0
SVBP	SVBP_tvX1	-2.7	0.01
MGST2	MGST2	-2.7	0
HIKESHI	HIKESHI	-2.7	0
SORD	SORD	-2.7	0
ABHD10	ABHD10	-2.7	0
VCPKMT	VCPKMT	-2.7	0
MKKS	MKKS	-2.7	0
CCNG1	CCNG1_tv	-2.7	0
COX7A2	COX7A2	-2.7	0
MMADHC	MMADHC	-2.8	0
TRAPPC13	TRAPPC13	-2.8	0
FXN	FXN_tv1	-2.8	0.05
PSMA6	PSMA6_tv2	-2.8	0
HEPACAM2	HEPACAM2	-2.8	0
PDCD10	PDCD10	-2.8	0

FAM114A2	FAM114A2	-2.8	0
SCOC	SCOC	-2.8	0
SERPINB6	SERPINB6_tv3	-2.8	0
TMEM126A	TMEM126A	-2.8	0
SMIM19	SMIM19	-2.8	0
H2AFVPs*	ENSSSCG00000041469	-2.8	0
SUMO4	SUMO4	-2.8	0
GRB14	GRB14	-2.8	0
C1D	ENSSSCG00000039331	-2.8	0
RPS7	RPS7	-2.8	0
CMBL	CMBL	-2.8	0
UGP2	UGP2	-2.8	0
ISCA2	ISCA2	-2.8	0
ATP5J2	ATP5J2_tv1	-2.8	0
CSKMT	CSKMT	-2.8	0
CYB561	CYB561	-2.8	0
OSTCL*	OSTCL*	-2.8	0
NIPSNAP2Ps*	ENSSSCG00000038176	-2.8	0.02
RNF141	RNF141_tv1	-2.8	0
CHCHD2L2*	CHCHD2L2*	-2.8	0
CYP27B1	CYP27B1	-2.8	0
SNAP23	SNAP23_tv2	-2.8	0.03
NDUFB5	NDUFB5	-2.8	0
CAPZA2	CAPZA2	-2.8	0
MRPS28	MRPS28	-2.8	0
LIAS	LIAS	-2.8	0
COMMD6	COMMD6_tv2	-2.8	0
LGALS1	LGALS1	-2.8	0
BEX2	ENSSSCG00000039273	-2.8	0
RBBP9	RBBP9	-2.8	0
C14orf119	C14orf119	-2.8	0
NDUFB9	NDUFB9	-2.8	0
SLC35F5	SLC35F5	-2.8	0
CKM	CKM	-2.8	0
RSL24D1	RSL24D1	-2.8	0
RPS18	RPS18	-2.8	0
TMEM70	TMEM70	-2.8	0
TMSB15B	TMSB15B_tv2	-2.8	0
UXT	UXT	-2.8	0
RPS18Ps2*	ENSSSCG00000046110	-2.8	0
RPL17-C18orf32	RPL17-C18orf32	-2.8	0
HADHB	HADHB_tv1	-2.8	0
RPS29	ENSSSCG00000038507	-2.8	0
TMEM258	ENSSSCG00000027538	-2.8	0
SLC25A14	SLC25A14_tvX3	-2.8	0
HSD17B7	HSD17B7	-2.8	0
PEX3	PEX3	-2.8	0

RPL35A	RPL35A	-2.8	0
CCDC58	CCDC58	-2.8	0
TMEM60	TMEM60	-2.8	0
PTPN22	PTPN22	-2.8	0
AP3S1	AP3S1_tv	-2.8	0
TMEM167B	TMEM167B	-2.8	0
RNF170	RNF170_tv2	-2.8	0
NEGR1	NEGR1	-2.8	0
ATP6	ATP6	-2.8	0.01
NDUFA1Ps*	ENSSSCG00000040694	-2.8	0
ATG4C	ATG4C_tv1	-2.8	0
NAALAD2	NAALAD2	-2.8	0
RPL14L*	ENSSSCG00000029003	-2.8	0
C8orf37	C8orf37	-2.8	0
GNPNAT1	GNPNAT1	-2.8	0
LAMTOR2	LAMTOR2_tv1	-2.9	0
SNAPC5	SNAPC5	-2.9	0
LOC110257263	ENSSSCG00000041925	-2.9	0
VMP1	VMP1	-2.9	0
NDUFV2	NDUFV2	-2.9	0
BRK1	BRK1	-2.9	0
RBIS	RBIS	-2.9	0
ENSSSCG00000047561	ENSSSCG00000047561	-2.9	0.03
ABHD18	ABHD18_tv4	-2.9	0.02
ACAD11	ACAD11_tv1	-2.9	0
PZP	PZP	-2.9	0.02
ENSSSCG00000043633	ENSSSCG00000043633	-2.9	0
MSMO1	MSMO1_tv1	-2.9	0
COX6A1	COX6A1	-2.9	0
NDUFS4	NDUFS4	-2.9	0
PRXL2A	PRXL2A_tv4	-2.9	0
CYB5A	CYB5A	-2.9	0
MRPL22	MRPL22	-2.9	0
CFL2	CFL2_tv2	-2.9	0
SMDT1	SMDT1	-2.9	0
RPS11	RPS11	-2.9	0
PHYH	PHYH_tv1	-2.9	0
SMIM26	SMIM26	-2.9	0
SCP2	SCP2_tv1	-2.9	0
MCEE	MCEE	-2.9	0
FASTKD2	FASTKD2_tv3	-2.9	0
RPL10A	ENSSSCG00000011206	-2.9	0
NME1	NME1	-2.9	0
COX5B	COX5B	-2.9	0
ENSSSCG00000045049	ENSSSCG00000045049	-2.9	0.03
NCK1	NCK1_tv	-2.9	0
PRSS35	PRSS35	-2.9	0

PPA2	PPA2	-2.9	0
CCDC68	CCDC68	-2.9	0.02
UCHL3	UCHL3	-2.9	0
ENSSSCG00000049194	ENSSSCG00000049194	-2.9	0.03
COPS2	COPS2	-2.9	0
RPS27L	RPS27L	-2.9	0
CCNB1IP1	CCNB1IP1	-2.9	0
GTPBP8	GTPBP8_tv1	-2.9	0
KRR1	KRR1	-2.9	0
ENSSSCG00000043294	ENSSSCG00000043294	-2.9	0.05
DGKB	DGKB	-2.9	0
ATP5C1	ATP5C1_tv1	-3	0
TNFAIP6	TNFAIP6	-3	0
ICAM3	ICAM3_tv1	-3	0.02
ACSM4	ACSM4_tv1	-3	0.03
NDUFC2	NDUFC2	-3	0
CIB4	CIB4	-3	0
ARPP19Ps*	ENSSSCG00000037176	-3	0
MED21	MED21	-3	0
SMIM8	SMIM8	-3	0
SAMD13	SAMD13	-3	0
PRKACB	PRKACB_tv1	-3	0
MTERF1	MTERF1	-3	0
PIH1D2	PIH1D2	-3	0
CYB5R4	CYB5R4	-3	0
MYOM2	MYOM2	-3	0
SNX31	SNX31	-3	0
DYNLL1-AS1*	ENSSSCG00000031756	-3	0.01
UQCRC2	UQCRC2	-3	0
SPP1	SPP1_tv1	-3	0
PI16	PI16_tv1	-3	0
FOLH1	FOLH1_tv1	-3	0
PRDX1	PRDX1_tv3	-3	0
COX1	COX1	-3	0
UQCRQ	UQCRQ	-3	0
NDUFA2	NDUFA2	-3	0
SKP1	SKP1	-3	0
FGL1	FGL1	-3	0
ENSSSCG00000045252	ENSSSCG00000045252	-3	0.01
APOO	APOO_tv1	-3	0
MRPS23	MRPS23	-3	0
LOC100621455	ENSSSCG00000041401	-3	0
LOC106510640	ENSSSCG00000047080	-3.1	0.04
ALPI	ALPI	-3.1	0
THYN1	THYN1_tv1	-3.1	0
CENPN	CENPN_tv2	-3.1	0
SELENOT	SELENOT	-3.1	0

COX7A2L	COX7A2L	-3.1	0
COQ10B	COQ10B	-3.1	0
CIDEA	CIDEA_tv1	-3.1	0
MROH5L*	ENSSSCG00000033737	-3.1	0
C11orf71	C11orf71	-3.1	0
TOMM5	TOMM5	-3.1	0
NDUFA11	NDUFA11	-3.1	0.01
AZIN1	AZIN1	-3.1	0
GMFB	GMFB_tv	-3.1	0
DECR1	DECR1	-3.1	0
CAPS2	CAPS2	-3.1	0.03
RPL22L1	ENSSSCG00000036114	-3.1	0
CDH3	CDH3	-3.1	0
RGN	RGN_tv2	-3.1	0
PSMA5	PSMA5_tv1	-3.1	0
C7H6orf125	C7H6orf125	-3.1	0
ARG1	ARG1_tv2	-3.1	0
ENSSSCG00000035654	ENSSSCG00000035654	-3.1	0
GSTA2	GSTA2	-3.1	0
PON2	PON2_tv1	-3.1	0
HPD_tv1	HPD_tv1	-3.1	0
BCKDHB	BCKDHB	-3.1	0
LOC106505711	ENSSSCG00000050456	-3.1	0
ETFA	ETFA	-3.1	0
ATP6AP2	ATP6AP2	-3.1	0
GNAT3	GNAT3	-3.1	0.03
SMIM14	SMIM14	-3.1	0
CDK7	CDK7	-3.2	0
RPS16	RPS16	-3.2	0
CLCA2	CLCA2	-3.2	0.04
TIAL1	TIAL1_tv2	-3.2	0
PMPCB	PMPCB	-3.2	0
LOC100521937	ENSSSCG00000046349	-3.2	0.01
ARRDC3	ARRDC3_tv1	-3.2	0
TIFAB	TIFAB	-3.2	0.05
TRAPPC5	TRAPPC5	-3.2	0
DMBT1	DMBT1_tv	-3.2	0.01
LncRNA	LncRNA	-3.2	0
WDR89	WDR89	-3.2	0
ATP5IF1	ATP5IF1	-3.2	0
RPS15A	RPS15A	-3.2	0
EMC3	EMC3	-3.2	0
RAB27B	RAB27B	-3.2	0
EFCAB10	EFCAB10_tv2	-3.2	0
SHAS2	SHAS2	-3.2	0.03
PDZK1IP1L	PDZK1IP1L	-3.2	0.03
DPPA2	DPPA2	-3.2	0.05

ISOC1	ISOC1	-3.2	0
PHKB	PHKB_tv2	-3.2	0.04
TSPAN12	TSPAN12_tvX1	-3.2	0
POMP	POMP	-3.2	0
NDUFB1	NDUFB1	-3.2	0
TMEM14C	TMEM14C	-3.2	0
RGS18	RGS18	-3.2	0
LY96	LY96	-3.2	0
MRPS10	MRPS10	-3.2	0
PIH1D3	PIH1D3	-3.3	0.01
RPS21	RPS21	-3.3	0
CTRB2L	CTRB2L	-3.3	0.02
NDUFS5	NDUFS5	-3.3	0
UBB	UBB	-3.3	0
NAT1	NAT1	-3.3	0
MRPL39	MRPL39	-3.3	0
CASP1	CASP1	-3.3	0
Pseudogene	Pseudogene	-3.3	0
ASB5	ASB5	-3.3	0
ENSSSCG00000042551	ENSSSCG00000042551	-3.3	0.01
TSG101	ENSSSCG00000041063	-3.3	0
KCNC2	KCNC2	-3.3	0
DNAJC19	DNAJC19	-3.3	0
AK6	AK6	-3.3	0
HAX1	HAX1_tv1	-3.3	0.02
ENSSSCG00000051063	ENSSSCG00000051063	-3.4	0.04
ATP5PO	ATP5PO	-3.4	0
RPS27	ENSSSCG00000031838	-3.4	0
SPA17	SPA17	-3.4	0
ATP5PD	ATP5PD	-3.4	0
TPD52L1	TPD52L1	-3.4	0.02
RSAD2	RSAD2	-3.4	0.02
TEFM	TEFM	-3.4	0
FANCF	FANCF	-3.4	0
PKIA	PKIA	-3.4	0
ECHS1	ECHS1	-3.4	0
CUL2	CUL2	-3.4	0
LYRM2	ENSSSCG00000004324	-3.4	0
ATP5MG	ATP5MG	-3.4	0
LOC110256489	ENSSSCG00000042665	-3.4	0
RPL39	ENSSSCG00000030849	-3.4	0
STMN2	STMN2	-3.4	0
RPS23L*	ENSSSCG00000012200	-3.4	0
MOB4	MOB4_tv1	-3.4	0
ABRACL	ABRACL	-3.4	0
ND3	ND3	-3.4	0
ENSSSCG00000043724	ENSSSCG00000043724	-3.4	0.01

COL6A6	COL6A6	-3.5	0.04
CBR4	CBR4_tv1	-3.5	0
SLIRP	SLIRP	-3.5	0
LACTB2	LACTB2	-3.5	0
FCGR3A	FCGR3A_tv3	-3.5	0
ROMO1	ROMO1	-3.5	0
SLX4IP	SLX4IP	-3.5	0
TMPRSS15	TMPRSS15	-3.5	0.01
COCH	COCH	-3.5	0
CROT	CROT_tv2	-3.5	0
PJVK	PJVK	-3.5	0
SRGN	SRGN_tv1	-3.5	0
IER3IP1	IER3IP1	-3.5	0
LOC110257240	LOC110257240_tv1	-3.5	0.01
GTF2H5	GTF2H5	-3.5	0
CIDEB	CIDEB_tv1	-3.5	0.02
TIMM8A	TIMM8A	-3.5	0
ALK	ALK	-3.5	0
EZR	EZR_tv1	-3.5	0.01
UQCR10	UQCR10	-3.5	0
ABI1	ABI1_tv14	-3.5	0.01
ENSSSCG00000041274	ENSSSCG00000041274	-3.5	0
PSMA2	PSMA2	-3.5	0
TMSB15A	ENSSSCG00000028695	-3.5	0
MORN2	MORN2	-3.6	0
HSPE1	HSPE1_tv1	-3.6	0
MRPL13	MRPL13	-3.6	0
PET100	PET100	-3.6	0
GLRX	GLRX_tv1	-3.6	0
MED31	MED31	-3.6	0.05
PLAC8	PLAC8_tv2	-3.6	0
LOC110257355	LOC110257355	-3.6	0.04
NDUFA1	NDUFA1	-3.6	0
DEFB123	DEFB123_tv1	-3.6	0
LOC110262153	ENSSSCG00000047803	-3.6	0
RXFP2	RXFP2	-3.6	0.01
CTSL	CTSL_tv1	-3.6	0
SF3B6	SF3B6	-3.6	0
LOC102160652	ENSSSCG00000046559	-3.6	0.04
MRPL50	MRPL50	-3.6	0
ENSSSCG00000034829	ENSSSCG00000034829	-3.6	0
RPS23Ps3*	ENSSSCG00000039020	-3.6	0
LOC106507633	ENSSSCG00000043385	-3.6	0.05
LOC102167757	ENSSSCG00000044335	-3.7	0.03
LRRC39	LRRC39	-3.7	0
TUBA1D	TUBA1D	-3.7	0.03
NIPAL1	NIPAL1	-3.7	0

CST3	CST3	-3.7	0
RPL11L	RPL11L	-3.7	0
TSTD1	TSTD1	-3.7	0
KLK1	KLK1	-3.7	0
MTRNR2L8	MTRNR2L8	-3.7	0
SERPINA3-1	SERPINA3-1	-3.7	0
FAAP24	FAAP24_tv1	-3.7	0.05
SCGB2A2	SCGB2A2	-3.7	0.03
RPH3A	RPH3A	-3.7	0
COX2	COX2	-3.7	0
FMC1	FMC1	-3.8	0
CD36L1	CD36L1	-3.8	0
ADAM3A	ADAM3A	-3.8	0.01
IL18	IL18_tv1	-3.8	0
RPL27Ps*	ENSSSCG00000007500	-3.8	0
SPACA1	SPACA1	-3.8	0.02
COX7B	COX7B	-3.8	0
SPTY2D1OS	ENSSSCG00000038037	-3.8	0.05
NDUFB11	NDUFB11	-3.8	0
CREG1	CREG1	-3.8	0.01
MT-ND4L	MT-ND4L	-3.9	0
SFRP2	SFRP2	-3.9	0.04
ATP5MC3	ATP5MC3	-3.9	0
AKR1C1	AKR1C1	-3.9	0
CCDC28A	CCDC28A	-3.9	0
GC	GC	-3.9	0
RPL22L1Ps*	ENSSSCG00000015460	-3.9	0
MT-CO2	MT-CO2	-3.9	0
PPM1K	PPM1K	-3.9	0
RPA3	RPA3	-4	0
TSACC	TSACC	-4	0.01
SLC25A16	SLC25A16	-4	0
ATP5PDL*	ENSSSCG00000016881	-4	0
GCA	GCA	-4	0
ENSSSCG00000049234	ENSSSCG00000049234	-4.1	0
CYP3A46	CYP3A46	-4.1	0
ENSSSCG00000042146	ENSSSCG00000042146	-4.1	0
RDH16	RDH16	-4.1	0
DEFB122	DEFB122	-4.1	0
HERPUD1	HERPUD1_tv1	-4.2	0.04
IFIT1L1*	IFIT1L1*	-4.2	0
CEACAM6L	CEACAM6L_tv1	-4.2	0.02
VSIG1	VSIG1_tv2	-4.2	0
Endonuclease/reverse transcriptase	ENSSSCG00000047266	-4.2	0.03
PRDX3	PRDX3	-4.2	0
SSMEM1	SSMEM1	-4.2	0
CISD1	ENSSSCG00000035762	-4.2	0

APOH	APOH	-4.2	0
DYNLT2B	DYNLT2B_tv2	-4.2	0
LCMT1	LCMT1_tv1	-4.2	0
RPS24Ps*	ENSSSCG00000016840	-4.3	0
RPS16Ps*	ENSSSCG00000005432	-4.3	0
SNX14	SNX14_tv1	-4.3	0.01
ENSSSCG00000047459	ENSSSCG00000047459	-4.3	0.02
IL13RA2	IL13RA2	-4.3	0
NT5C3	NT5C3	-4.3	0.01
TGM5	TGM5	-4.3	0
lncRNA	lncRNA	-4.3	0
ADH1C	ADH1C	-4.3	0.01
KLRK1	KLRK1_tv	-4.4	0
C5orf58	C5orf58	-4.4	0
TIGD4	TIGD4	-4.4	0
LOC110259852	ENSSSCG00000036448	-4.4	0.02
GPD1L2*	ENSSSCG00000004243	-4.4	0
ADAM5	ADAM5	-4.5	0
TMEM27	TMEM27	-4.5	0
MT-TP	ENSSSCG00000018096	-4.5	0
SLA-DRB4	SLA-DRB4	-4.5	0.04
ARPC3	ARPC3	-4.6	0
ASPA	ASPA_tv1	-4.6	0.01
RPL23AL*	RPL23AL*	-4.6	0
ARL15	ENSSSCG00000048060	-4.6	0
MIR22	MIR22	-4.6	0
CHRNA6	CHRNA6_tv1	-4.6	0
S100A12	S100A12	-4.6	0
ADAM5	ADAM5	-4.7	0
LYZ	LYZ-1	-4.7	0
DEFB119	DEFB119	-4.7	0.03
FBP2	FBP2	-4.7	0
CYP3A29	CYP3A29	-4.8	0
PNLIPRP2	PNLIPRP2	-4.8	0
LncRNA	LncRNA	-4.9	0
STAP2	STAP2_tv2	-4.9	0
CDX4	CDX4	-4.9	0
ANKRD66	ANKRD66	-4.9	0.01
MAGEB18	MAGEB18	-5	0
DTX1-AS*	ENSSSCG00000047984	-5	0.01
TRAV8-3	ENSSSCG00000034110	-5	0
SNORA63	SNORA63	-5.1	0
CEACAM1L*	ENSSSCG00000038419	-5.2	0
MRAP2	MRAP2_tvX1	-5.2	0.01
ZNF226	ZNF226_tv7	-5.2	0.04
CDH19	CDH19_tv1	-5.2	0.04
LOC110261477	LOC110261477	-5.2	0

PON1	PON1	-5.2	0.04
FABP1	FABP1	-5.2	0.04
TMPRSS11C	ENSSSCG00000029658	-5.3	0.02
IFI6	IFI6	-5.3	0
HCST	ENSSSCG00000002918	-5.3	0.05
NDST4	NDST4_tv1	-5.4	0
IGJ	IGJ_tv1	-5.4	0
IFI27	ISG12(A)	-5.6	0
GDAP1L1	GDAP1L1	-5.6	0
PANK1	PANK1_tvb	-5.6	0
API5	API5_tv2	-5.6	0
MIR1244-1	MIR1244-1	-5.7	0
LHFPL5	LHFPL5	-5.7	0.01
CYP11B2	CYP11B2	-5.7	0
IFRD1	IFRD1_tv1	-5.7	0.02
HSD17B2	HSD17B2	-5.7	0
RPS27APs*	ENSSSCG00000035890	-5.8	0
ENSSSCG00000037148	ENSSSCG00000037148	-5.8	0
LncRNA	LncRNA	-5.8	0.03
GZMH	GZMH	-5.9	0
lncRNA	lncRNA	-5.9	0
ETFRF1	ETFRF1	-6	0
CD59	CD59	-6	0
ANKRD22	ANKRD22	-6.1	0
CA5B	CA5B	-6.2	0
LOC102165115	ENSSSCG00000041917	-6.3	0
TFRC	TFRC_tv1	-6.3	0
lncRNA	lncRNA	-6.4	0
UNK37*	UNK37*	-6.5	0
LOC102159522	ENSSSCG00000041230	-6.7	0
ENSSSCG00000049111	ENSSSCG00000049111	-6.7	0.03
UGT2A1	UGT2A1_tv1	-6.7	0
SAA3	SAA3_tv1	-6.8	0
Y_RNA_4	Y_RNA_4	-6.8	0
LOC102165231	LOC102165231	-7	0.01
SNORA8	SNORA8	-7.1	0.01
ALG13	ENSSSCG00000012586	-7.2	0
CLEC7A	CLEC7A	-7.3	0.01
CXCL8	CXCL8_tv1	-7.4	0.02
CLEC12B	CLEC12B_tv1	-7.4	0.02
ENSSSCG00000047986	ENSSSCG00000047986	-7.5	0.02
NUGGC	NUGGC	-7.8	0
CLPS	CLPS_tv1	-7.8	0
CXCL13	CXCL13	-8	0
ATP5MJL	ENSSSCG00000016791	-8	0
MDFIC2	MDFIC2	-8.2	0.01
NCBP2L	NCBP2L	-8.3	0.01

LBP	LBP	-8.5	0
lncRNA	lncRNA	-8.8	0
SDSL	SDSL_tv1	-9.7	0
DPPA3	DPPA3	-10	0
ENSSSCG00000041918	ENSSSCG00000041918	-10.3	0.02
TMEM210	TMEM210	-10.6	0
FAM167B	FAM167B	-11.5	0.02
PR39	PR39	-11.9	0.01
LECT2	LECT2	-12.8	0.01
Endonuclease/reverse transcriptase	Endonuclease/reverse transcriptase	-15	0
RBM18	RBM18_tv1	-15.1	0
Endonuclease/reverse transcriptase	ENSSSCG00000040992	-18.3	0
GZMA	GZMA	-18.8	0
UNK95*	UNK95*	-22.9	0
LOC102162756	ENSSSCG00000047396	-36.3	0
TEDDM1L2*	TEDDM1L2*	-37.9	0
PHYHIPL	PHYHIPL	-39.4	0
TRIM15	TRIM15	-49.6	0
