# Supplementary material for: Soy Formula Is Not Estrogenic and Does Not Result in Reproductive Toxicity in Male Piglets: Results from a Controlled Feeding Study
Source: Nutrients. 2022 Mar 7;14(5):1126. doi: 10.3390/nu14051126 (PMC8912539; doi:10.3390/nu14051126)
Supplement: Supplementary file 1 [file nutrients-14-01126-s001.zip › Supplemental table 2.pdf]

Reannotated Gene	Gene	Fold Change	FDR
SFTA2	SFTA2	76.4	0.05
ATP6	ATP6	52.8	0.04
PDE4A	PDE4A_tv2	41.3	0.02
SLCO4A1	SLCO4A1	38.9	0.03
ENSSSCG00000049906	ENSSSCG00000049906	36.1	0.04
DEFB115	DEFB115	27.1	0.01
TSPAN1	TSPAN1_tv1	23.9	0.03
HOXB9	HOXB9	16.6	0.01
UNK129*	UNK129	15.1	0
PTPRO	PTPRO	14.9	0.04
LEFTY2	LEFTY2_tv1	13.5	0.04
TRPM5	TRPM5	12.9	0.04
CIZ1	CIZ1_tvX11	11	0.01
RRP8	RRP8	10.9	0.03
CYP1A1	CYP1A1	10.7	0.03
HOXB6	HOXB6	10.7	0.02
ENSSSCG00000045545	ENSSSCG00000045545	10.4	0.02
HYOU1	HYOU1_tv2	10.3	0.03
NOTCH3	NOTCH3_tv1	10.1	0.01
EPCAM	EPCAM	9.3	0.02
SULT1A1	SULT1A1_tv	9.2	0.02
MEIS2	MEIS2_tva	8.7	0
PRR7	PRR7	8.6	0.04
KRT19	KRT19	8.3	0
CX3CL1	CX3CL1_tv1	8.2	0.04
MATN4	MATN4	7.8	0.02
LOC110262312	ENSSSCG00000044344	6.8	0.02
TACSTD2	TACSTD2	6.8	0.03
PRSS8	PRSS8	6.7	0.02
GATA1	GATA1	6.5	0.03
ARHGEF5	ARHGEF5	6.2	0.05
AHSP	AHSP_tv1	6.2	0
PTGIR	PTGIR	6.2	0
R3HDM2	R3HDM2	6.2	0
NR2F1	NR2F1_tv1	6	0
NDUFA4L2	NDUFA4L2	6	0.03
RERE	RERE_tv2	5.8	0.03
KCNMB1	KCNMB1_tv1	5	0.05
COL26A1	COL26A1	5	0.03
NRIP2	NRIP2	5	0.01
SLPI	SLPI	4.9	0.02
CPZ	CPZ	4.9	0.04
NGF	NGF	4.6	0.03
LOC100514340	ENSSSCG00000032383	4.6	0
NUMA1	NUMA1_tvX1	4.5	0
ENSSSCG00000042133	ENSSSCG00000042133	4.4	0.02

PTGER1	PTGER1	4.4	0.04
NPTX1	NPTX1	4.4	0.01
FCGBP	FCGBP	4.4	0.04
CACNA1B	CACNA1B_tv1	4.4	0.03
ZAP70	ZAP70	4.4	0.05
MUC6	MUC6	4.3	0.03
NKD1	NKD1	4.3	0
ALOX12	ALOX12	4.3	0
UGT1A1	ENSSSCG00000036274	4.3	0
LncRNA	LncRNA	4.1	0
RGS12	RGS12_tv2	4.1	0
LMOD1	LMOD1	4.1	0.01
SYT15	ENSSSCG00000028341	4.1	0.01
LOC106509797/LOC110259996	ENSSSCG00000044592	4.1	0.02
ZNF883	ENSSSCG00000047560	4	0.03
LINGO1	LINGO1	4	0
CAPN6	CAPN6	3.8	0.03
OXTR	OXTR_tv1	3.8	0.02
FZD9	FZD9	3.8	0.02
ENSSSCG00000044418	ENSSSCG00000044418	3.8	0.04
HAL	HAL	3.8	0
HOXB4	HOXB4	3.8	0.03
LAD1	LAD1	3.8	0.03
ADAMTS16	ADAMTS16_tv1	3.8	0.01
TM9SF1	TM9SF1_tv2	3.7	0.03
ENSSSCG00000018657	ENSSSCG00000018657	3.7	0.02
PCDH1	PCDH1_tv2	3.7	0
PYGM	PYGM_tv1	3.6	0.01
BRSK2	BRSK2	3.6	0.04
LOC110260790	ENSSSCG00000049984	3.6	0.04
CXCL12	CXCL12_tv3	3.6	0.01
SIAH3	SIAH3	3.5	0.01
LAMA5	ENSSSCG00000032761	3.4	0
SEH1L	SEH1L_tv1	3.3	0.04
CD248	CD248	3.3	0
ADRA1D	ADRA1D	3.3	0.04
RRBP1	RRBP1	3.3	0
TBX1	TBX1	3.3	0
ID4	ID4	3.3	0.03
JPH4	JPH4_tv1	3.3	0.04
CD300CL*	CD300CL*	3.3	0.04
NES	NES	3.2	0
HPDL	HPDL	3.2	0
NOVA2	NOVA2	3.2	0.01
HAS1	HAS1	3.2	0.01
APLNR	APLNR	3.2	0
RTN4RL2	RTN4RL2	3.2	0.01

MYH14	MYH14_tv1	3.1	0
NCR3	NCR3	3.1	0
HOXA5	HOXA5	3.1	0.04
COL18A1	COL18A1_tv2	3.1	0
LYNX1	LYNX1	3.1	0.01
SPTBN4	ENSSSCG00000037494	3.1	0.04
MYH9	MYH9	3.1	0
SDC3	SDC3	3.1	0.02
MYH10	ENSSSCG00000038144	3.1	0
HMGN5	HMGN5	3.1	0
SSUH2	SSUH2	3	0.05
SHANK1	SHANK1_tv1	3	0.01
PLVAP	PLVAP	3	0
STAC2	STAC2	3	0.04
CD2APL1	CD2APL1	3	0.05
APLN	APLN	3	0
TMEM37	TMEM37	3	0
ANKRD35	ANKRD35	3	0.03
TAFA5	TAFA5	3	0.01
MOXD1	MOXD1	3	0.02
GPR4	GPR4	3	0
WFDC2	WFDC2	3	0.04
COL13A1	COL13A1_tv1	2.9	0
ADAMTSL2	ADAMTSL2	2.9	0.01
MEGF6	MEGF6	2.9	0
CACNA1C	ENSSSCG00000027725	2.9	0.02
MXRA5	ENSSSCG00000012832	2.9	0
ITGA3	ITGA3_tva	2.9	0.01
CLEC14A	CLEC14A	2.9	0.02
ACTB	ACTB	2.9	0
GUSB	GUSB	2.9	0
BDKRB2	BDKRB2_tvX2	2.9	0.02
SPTA1	SPTA1	2.9	0.02
PLAT	PLAT_tv1	2.9	0.02
RNF183	RNF183	2.9	0.05
CD93	CD93	2.9	0
ADGRL1	ADGRL1_tv1	2.8	0
CYP2S1	CYP2S1	2.8	0
TMEM151A	TMEM151A	2.8	0.01
SAMD11	SAMD11	2.8	0
PTMS	PTMS	2.8	0
NEURL1B	NEURL1B	2.8	0
LLGL2	LLGL2	2.8	0.01
SF1	SF1_tv6	2.8	0.05
SPSB1	SPSB1	2.8	0.01
CERCAM	CERCAM	2.8	0.01
EPPK1	EPPK1	2.8	0.01

CNN2	CNN2	2.8	0.04
GLI2	GLI2	2.8	0
KCNK6	KCNK6	2.8	0.01
LRRC8D	LRRC8D	2.8	0.03
NIM1K	NIM1K	2.7	0
HOXC8	HOXC8	2.7	0.02
PLEC	PLEC	2.7	0
IGF2-AS	IGF2-AS	2.7	0.04
MEIS3	MEIS3	2.7	0.03
ADGRL3	ADGRL3_tv1	2.7	0.01
WWC1	WWC1	2.7	0.02
ELN	ELN	2.7	0.01
MAP1A	MAP1A	2.7	0
ZNF503	ZNF503	2.7	0
ABCA9	ABCA9_tv1	2.7	0
PHF2	PHF2	2.7	0
ABLIM2	ABLIM2_tv1	2.7	0.04
SPACA6	SPACA6	2.7	0.01
MFAP2	MFAP2	2.7	0.02
FLNA	FLNA_tv1	2.7	0
ENSSSCG00000041780	ENSSSCG00000041780	2.7	0.05
COL4A2	COL4A2	2.7	0
PKD1	PKD1	2.6	0
PPP1R12B	PPP1R12B	2.6	0
TPM2	TPM2	2.6	0.03
SCARF2	SCARF2	2.6	0
COL6A1	COL6A1	2.6	0.01
COL7A1	COL7A1_tv1	2.6	0.03
CELSR1	CELSR1	2.6	0
TMEM120B	TMEM120B	2.6	0.02
ACTN1	ACTN1	2.6	0.03
JAG2	JAG2_tv1	2.6	0
TIGD7	TIGD7	2.6	0.04
ANO1	ANO1	2.6	0
NR2F2	NR2F2	2.6	0
ITGA7	ITGA7	2.6	0
PLXNB3	PLXNB3	2.6	0
ENSSSCG00000051659	ENSSSCG00000051659	2.5	0
TRPV4	TRPV4	2.5	0.02
CCDC102A	CCDC102A	2.5	0
MOB2	MOB2	2.5	0.01
SPOCK1	SPOCK1	2.5	0
ENSSSCG00000049957	ENSSSCG00000049957	2.5	0.05
HIRIP3	HIRIP3_tv1	2.5	0
HLX	HLX_tv1	2.5	0
FOXC1	FOXC1	2.5	0.03
THSD4	THSD4_tvX1	2.5	0.02

ANKRD24	ANKRD24	2.5	0
RTN4RL1	RTN4RL1	2.5	0.01
KIFC3	KIFC3	2.5	0
TNFRSF6B	ENSSSCG00000036595	2.5	0.01
ENSSSCG00000049992	ENSSSCG00000049992	2.5	0.01
NR1H2	NR1H2	2.5	0.01
EFNB3	EFNB3	2.5	0.02
DZIP1L	DZIP1L_tv1	2.5	0.05
MRC2	MRC2	2.5	0
PRKAR1B	PRKAR1B	2.5	0
NOTCH1	NOTCH1	2.5	0
PCDH12	PCDH12	2.4	0
NTN3	NTN3	2.4	0.02
TBXA2R	TBXA2R	2.4	0.02
IGF2	IGF2_tv1	2.4	0.01
MFGE8	MFGE8_tv3	2.4	0.01
MROH5	ENSSSCG00000033726	2.4	0.01
H19	H19_tv1	2.4	0
IER5L	IER5L_tv1	2.4	0
SUSD2	ENSSSCG00000025393	2.4	0.04
PENK	PENK	2.4	0
CROCC	CROCC_tv	2.4	0.01
NIPAL4	NIPAL4	2.4	0.03
AP2A1	AP2A1	2.4	0
PIEZO1	PIEZO1	2.4	0
ROBO4	ROBO4	2.4	0
PTRF	PTRF	2.4	0
HR	HR	2.4	0.01
CALD1	CALD1_tv1	2.4	0
CCDC106	CCDC106	2.4	0.03
EFCC1	EFCC1	2.4	0
TNR	TNR	2.4	0.01
MAPKAPK2	MAPKAPK2	2.4	0.01
COL4A1	COL4A1	2.4	0
SMAD7	SMAD7	2.4	0.01
EHBP1L1	EHBP1L1_tvX1	2.4	0
DCHS1	DCHS1_tv1	2.4	0
PTGDS	PTGDS	2.4	0.05
SDK1	SDK1	2.4	0.01
VWF	VWF	2.4	0
EIF5B	EIF5B	2.4	0
LTBP1	LTBP1_tv1	2.4	0.04
IQANK1	IQANK1	2.4	0.01
LDLRAD2	LDLRAD2	2.4	0.01
SYNGAP1	SYNGAP1	2.3	0.01
TMEM214	TMEM214_tv2	2.3	0
SSC5D	SSC5D_tv1	2.3	0.02

PIK3R2	PIK3R2	2.3	0.05
GPRC5C	GPRC5C	2.3	0
LRRC3	LRRC3	2.3	0.04
SHB	SHB	2.3	0
SH3TC1	SH3TC1	2.3	0
CAMK2N1	ENSSSCG00000024009	2.3	0.01
FTH1P1	FTH1P1	2.3	0.01
TBX6	TBX6	2.3	0.04
FAM171A2	FAM171A2	2.3	0
TRPM4	TRPM4	2.3	0.01
ADAMTS7	ADAMTS7	2.3	0
RBM19	RBM19	2.3	0.04
MYH7B	MYH7B_tv1	2.3	0
ASTN1	ASTN1_tvX1	2.3	0
PLEKHA6	PLEKHA6	2.3	0.02
TNFRSF11A	TNFRSF11A	2.3	0
THBS4	THBS4	2.3	0
COL5A1	COL5A1	2.3	0.05
GREB1	GREB1	2.3	0
ADGRA2	ADGRA2_tv1	2.3	0
RPS6KA2	RPS6KA2	2.3	0
SEMA6B	SEMA6B	2.3	0
TRIM16	TRIM16_tv1	2.3	0.02
EMILIN1	EMILIN1	2.3	0
TSPAN9	TSPAN9_tv1	2.3	0
BCL3	BCL3_tvX1	2.3	0
DYSF	DYSF	2.3	0
LIMS2	LIMS2	2.3	0.01
RHOG	ENSSSCG00000039656	2.2	0.03
TP53INP2	TP53INP2_tv1	2.2	0.01
PDE2A	PDE2A	2.2	0
SOX18	SOX18	2.2	0
CEP250	CEP250	2.2	0
NGFR	NGFR	2.2	0.01
ARHGAP39	ARHGAP39	2.2	0.01
BGN	BGN	2.2	0.01
APBB2	APBB2	2.2	0.02
CA11	CA11_tv1	2.2	0
EPHB3	EPHB3	2.2	0
FAM155A	FAM155A	2.2	0.05
FGF22	FGF22_tvX1	2.2	0.05
PLSCR3	PLSCR3	2.2	0
CDH5	CDH5	2.2	0
BOK	BOK_tv1	2.2	0.04
MYADM	MYADM_tv2	2.2	0
FAM43A	FAM43A	2.2	0
PLXND1	PLXND1	2.2	0

PDE10A	PDE10A	2.2	0.02
KCNN3	KCNN3	2.2	0.01
PDXK	PDXK	2.2	0.02
CREB3L1	CREB3L1	2.2	0
FGFR1	FGFR1	2.2	0.01
ARHGEF17	ARHGEF17_tvX1	2.2	0
SLC38A10	SLC38A10_tv1	2.2	0
SLC45A4	SLC45A4	2.2	0.01
AEBP1	AEBP1	2.2	0.02
ZC3H3	ZC3H3	2.2	0.04
TRIOBP	TRIOBP_tv1	2.2	0
PRAG1	PRAG1_tvX1	2.2	0
ATOH8	ATOH8	2.2	0.05
CCM2L	CCM2L	2.2	0
AFAP1L1	AFAP1L1	2.2	0
SHANK3	SHANK3	2.2	0
DES	DES	2.2	0.02
ARHGEF40	ARHGEF40	2.2	0
MYLK	MYLK	2.2	0
RELA	RELA	2.2	0.04
AS3MT	AS3MT	2.2	0.02
PTPN6	PTPN6	2.2	0
ADGRF5	ADGRF5	2.2	0.02
CARNS1	CARNS1	2.2	0.02
NFIX	NFIX	2.2	0
SOX7	ENSSSCG00000031889	2.2	0.04
SMAD6	SMAD6	2.2	0.02
WDR86	WDR86_tv2	2.2	0.03
FLT1	FLT1_tv1	2.2	0
SLX4	SLX4_tv1	2.2	0.02
ZC3H13	ZC3H13_tvX8	2.2	0
FAM124B	FAM124B	2.2	0.03
GPR173	GPR173	2.2	0
SEMA3F	SEMA3F	2.2	0.01
SEPTIN8	SEPTIN8	2.1	0.01
SCN1B	SCN1B	2.1	0
COL9A2	COL9A2	2.1	0.01
ZNF853	ZNF853	2.1	0
ZNF316	ZNF316	2.1	0.01
LOC110260611	ENSSSCG00000035352	2.1	0.05
ARAP3	ARAP3	2.1	0
TCHP	TCHP	2.1	0
SLC6A17	SLC6A17	2.1	0.04
P3H3	P3H3	2.1	0.01
LAMB3	LAMB3_tv1	2.1	0.02
SPRY4	SPRY4	2.1	0.01
UVSSA	UVSSA	2.1	0

OS9	OS9_tv1	2.1	0
PPRC1	PPRC1	2.1	0
AGRN	AGRN	2.1	0
DIPK2B	DIPK2B	2.1	0.04
TLN1	TLN1	2.1	0
WFS1	WFS1	2.1	0
CDH24	CDH24_tv2	2.1	0.01
NIBAN2	NIBAN2	2.1	0.02
BCL9L	BCL9L	2.1	0
PDGFRB	PDGFRB	2.1	0
ZBTB46	ZBTB46_tvX3	2.1	0
ZNF467	ZNF467_tv1	2.1	0.02
CPXM2	CPXM2	2.1	0.02
LIPE	LIPE	2.1	0.03
PER2	PER2	2.1	0.01
LRATD2	LRATD2	2.1	0.01
SELENOO	SELENOO	2.1	0.01
SLC2A10	SLC2A10	2.1	0.03
KCNK12	KCNK12	2.1	0.01
GJA4	GJA4	2.1	0
MMP15	MMP15	2.1	0
MAP2K7	MAP2K7	2.1	0.04
HNRNPUL2	HNRNPUL2	2.1	0
LAMC1	LAMC1	2.1	0
LAMB2	LAMB2	2.1	0
TIE1	TIE1	2.1	0
LTBP4	LTBP4	2.1	0
FHAD1	FHAD1	2.1	0.04
BCAM	BCAM_tv1	2.1	0
POLR2A	POLR2A	2.1	0.01
SRCAP	SRCAP_tv1	2.1	0
PAPLN	PAPLN	2.1	0
CSPG4	CSPG4	2.1	0
PPFIBP2	PPFIBP2	2.1	0.01
IGFBP5	IGFBP5	2.1	0
JUP	JUP	2.1	0.01
UNC5B	UNC5B	2.1	0
KIAA0930	KIAA0930	2.1	0.04
IGFBP7	IGFBP7	2.1	0.01
EGR1	EGR1	2.1	0
LOXL3	LOXL3	2.1	0.01
FADD	FADD	2.1	0.04
TCOF1	TCOF1_tvX3	2.1	0
CRTC1	CRTC1	2.1	0.04
ENG	ENG_tv1	2.1	0
DENND2B	DENND2B	2.1	0
SMTN	SMTN	2.1	0

AKAP12	AKAP12	2.1	0
CUEDC1	CUEDC1	2.1	0.04
BICRA	BICRA	2.1	0
SLC3A2	SLC3A2_tv1	2.1	0
CHD7	CHD7_tv1	2.1	0.01
MAP2K3	MAP2K3	2.1	0.04
HSPA12B	HSPA12B	2.1	0.01
MRPL58	MRPL58	2.1	0.01
LOC110257381	ENSSSCG00000044439	2.1	0.03
CCND1	CCND1	2.1	0
EMID1	EMID1	2.1	0.01
STK24	STK24	2.1	0.01
SOGA1	SOGA1	2.1	0
PLXDC2	PLXDC2_tv1	2.1	0.04
TSPAN15	TSPAN15_tv1	2.1	0.01
ERBB3	ERBB3_tv1	2	0.01
ETV6	ETV6	2	0.01
CDR2	CDR2	2	0.04
MTA1	MTA1_tv1	2	0.05
CBX6	CBX6	2	0
ABCC10	ABCC10	2	0.04
FZD8	FZD8	2	0
SLC22A17	SLC22A17	2	0.05
GALNT18	GALNT18	2	0.04
MAP7D1	MAP7D1_tv1	2	0
FBLN2	FBLN2	2	0
DAAM2	DAAM2_tv1	2	0
NCL	NCL	2	0
TNK2	TNK2_tv1	2	0
OSBPL5	OSBPL5_tv1	2	0.02
TRAF7	TRAF7	2	0.03
CHST1	CHST1	2	0
LAMC3	LAMC3_tv1	2	0
KIF7	KIF7	2	0
PCNT	PCNT_tv1	2	0
ZSCAN20	ENSSSCG00000042252	2	0.01
PMEPA1	PMEPA1	2	0
SHROOM1	SHROOM1	2	0.02
RARG	RARG_tv2	2	0.03
FLT4	FLT4	2	0
DYNC1H1	DYNC1H1	2	0.01
NSD2	NSD2	2	0.02
DAP	DAP	2	0.01
TAF3	TAF3	2	0
NRAP	NRAP	2	0.01
CARD10	CARD10	2	0
SKI	SKI	2	0

MEX3D	MEX3D	2	0.04
EHD3	EHD3	2	0
CDK18	CDK18_tv3	2	0
AGPAT3	AGPAT3	2	0.01
BCORL1	BCORL1	2	0.01
SHROOM2	SHROOM2	2	0.02
CASKIN1	CASKIN1	2	0.04
NCK2	NCK2	2	0.03
NOS1AP	NOS1AP	2	0.01
PLEKHG2	PLEKHG2_tvX3	2	0
EIF3A	EIF3A	2	0
BAHCC1	ENSSSCG00000023045	2	0
MVD	MVD	2	0
TSPAN11	TSPAN11_tv1	2	0.01
SYT9	SYT9	2	0.02
DOC2B	DOC2B	2	0
MED24	MED24	2	0.01
SCD	SCD	2	0
MPRIP	MPRIP	2	0
MYO19	MYO19	2	0.02
PRR12	PRR12_tv1	2	0
HDGFRP2	HDGFRP2_tv1	2	0
TRIM26	TRIM26	2	0.03
FILIP1	FILIP1	2	0.02
TAF15	TAF15	2	0
NFIC	NFIC	2	0
AFDN	AFDN_tvX10	2	0
PHRF1	PHRF1	2	0
IMPDH1	IMPDH1	2	0.02
ARID1A	ARID1A_tv1	2	0
SPNS2	SPNS2	2	0
EPN2	EPN2	2	0
SLC38A5	SLC38A5	2	0
TNRC18	TNRC18	2	0.01
N4BP3	N4BP3	2	0
NBEAL2	NBEAL2_tv1	2	0
BAG3	BAG3	2	0
LTBP3	LTBP3	2	0
CHD3	CHD3_tv1	2	0
FAM129B	FAM129B	2	0
COL24A1	ENSSSCG00000031738	2	0.03
CDC42EP1	CDC42EP1	2	0
ALS2CL	ALS2CL_tv1	2	0.01
PPP4C	PPP4C	2	0.02
GPR35	GPR35	2	0.01
PIK3R2	PIK3R2	2	0
EML3	EML3	2	0.02

ACVRL1	ACVRL1_tv1	2	0
PPP1R9B	PPP1R9B	2	0
TGFB1I1	TGFB1I1	2	0.05
OLFML2A	OLFML2A_tv1	2	0
ZNF276	ZNF276_tva	2	0
CARF	CARF	-2	0
PDK4	PDK4	-2	0.02
MIS12	MIS12_tv3	-2	0
QRSL1	QRSL1	-2	0
HBP1	HBP1_tv1	-2	0
BBS10	BBS10	-2	0.04
PSAT1	PSAT1_tv1	-2	0
GAS2L3	GAS2L3	-2	0
NR1D2	NR1D2_tv1	-2	0
CDKL3	CDKL3	-2	0.04
COMMD2	COMMD2	-2	0
VAMP7	VAMP7	-2	0
SESN1	SESN1_tv1	-2	0
DRG1	DRG1	-2	0
SPCS3	SPCS3	-2	0
METTL8	METTL8_tv1	-2	0.01
IMMP1L	IMMP1L	-2	0.01
TRIQK	TRIQK	-2	0.01
PRKCI	PRKCI	-2	0
SLC7A11	SLC7A11	-2	0.01
LOC106508844	ENSSSCG00000050757	-2	0.04
LSM5	ENSSSCG00000026064	-2	0.01
PRIM1	ENSSSCG00000026055	-2	0
PRDX2	PRDX2	-2	0
ATP1B3	ATP1B3	-2	0
CETN2	ENSSSCG00000012757	-2	0
MRPS36	MRPS36	-2	0
FCF1	FCF1	-2	0
C4orf46	C4orf46	-2	0.02
PRPF39	PRPF39	-2	0
PNRC2	PNRC2	-2	0
C17orf75	C17orf75	-2	0.01
KLHL28	KLHL28	-2	0
ORC6	ORC6	-2	0
C18orf21	C18orf21	-2	0
ZC2HC1A	ZC2HC1A	-2	0.01
CGRRF1	CGRRF1	-2	0
NDUFV2	NDUFV2	-2	0
MSR1	MSR1_tvSR-II	-2	0.04
CENPH	CENPH	-2	0.01
PCNA	PCNA	-2	0
ASB9	ASB9	-2	0.01

GSTA2	GSTA2	-2	0
EIF1AX	EIF1AX	-2	0
ZSCAN31	ZSCAN31	-2	0.01
FBXO8	FBXO8	-2	0.01
LOC102160564	ENSSSCG00000041434	-2	0.01
RFC4	RFC4_tv1	-2	0
PTP4A1	PTP4A1	-2	0
PPIB	PPIB	-2	0
UXT	UXT	-2	0.02
PMPCB	PMPCB	-2	0
COMMD6	COMMD6_tv2	-2	0
TBC1D15	TBC1D15	-2	0
TRIM59	TRIM59_tv1	-2	0
CCDC32	CCDC32	-2	0
PRXL2A	PRXL2A_tv4	-2	0
ATP6AP2	ATP6AP2	-2	0
ZNF350	ZNF350	-2	0.02
PRORSD1	ENSSSCG00000037399	-2	0.04
ACTR10	ACTR10	-2	0
EXOSC8	EXOSC8	-2	0.01
TMEM126A	TMEM126A	-2	0
UFM1	UFM1	-2	0
ATL2	ATL2	-2	0
LOC100627283	ENSSSCG00000030015	-2	0
SH3BGRL	ENSSSCG00000012452	-2	0
RBM46	RBM46	-2	0.04
SRSF7	SRSF7	-2	0
TMEM263	TMEM263	-2	0
SOHLH2	ENSSSCG00000026564	-2	0.02
NUP35	NUP35	-2	0
RBIS	RBIS	-2	0
C21orf91	C21orf91	-2	0
CLEC2D	CLEC2D_tv1	-2	0
SMARCE1	SMARCE1	-2	0
LOC100626258	ENSSSCG00000044256	-2	0.04
C12orf29	C12orf29	-2	0.02
TAF1D	TAF1D	-2	0
THAP6	THAP6	-2	0
GNPNAT1	GNPNAT1	-2	0
SMIM19	SMIM19	-2	0
ZNF548L	ZNF548L	-2	0.01
HSF2	HSF2_tv1	-2	0
RRAGD	RRAGD	-2	0.01
TPST2	TPST2	-2	0
CSE1L	CSE1L_tv	-2	0
SMDT1	SMDT1	-2	0
OSTC	OSTC	-2	0

UBE2B	UBE2B	-2	0
CYCS	CYCS	-2	0
C8orf76	ENSSSCG00000021744	-2	0.01
PFDN4	PFDN4	-2	0
ORC4	ORC4	-2	0
DSCC1	DSCC1	-2	0.03
BORCS7	BORCS7	-2	0
CHODL	CHODL	-2	0
TNFSF10	TNFSF10_tv1	-2	0.01
UQCR10	UQCR10	-2	0
NPL	NPL	-2	0
C1D	ENSSSCG00000039331	-2	0
CUTC	CUTC_tv1	-2	0
CYB5A	CYB5A	-2	0
IER3IP1	IER3IP1	-2	0
FUNDC1	FUNDC1	-2	0
METTL5	METTL5	-2	0
S100A2	S100A2_tv	-2	0.04
FANCD2	FANCD2_tv2	-2	0.01
PIH1D2	PIH1D2	-2	0.04
RPS4X	RPS4X	-2	0
TPRKB	TPRKB	-2	0
GIN1	GIN1	-2	0
PAIP1	PAIP1	-2	0
NUBPL	NUBPL	-2	0
RPL7A	RPL7A	-2	0
FGFR1OP2	FGFR1OP2	-2	0
NIT2	NIT2	-2	0
UQCRQ	UQCRQ	-2	0
ACP1	ACP1_tv3	-2	0
TRIM23	TRIM23_tv1	-2	0
CFAP20	CFAP20	-2	0
BOLA1	BOLA1	-2	0
RPL13A	RPL13A_tv	-2	0
COMMD3	COMMD3	-2	0
AK3	AK3	-2	0.05
TNFRSF17	TNFRSF17	-2	0.01
SUCLG2	SUCLG2_tv2	-2	0
ERGIC2	ERGIC2	-2	0
PI15	PI15	-2	0
CXADR	CXADR_tv1	-2	0
YOD1	YOD1	-2	0.01
BZW1	BZW1	-2	0
VCPKMT	VCPKMT	-2	0.01
PDIK1L	PDIK1L	-2	0
MIR9810	MIR9810	-2	0.01
ATP5C1	ATP5C1_tv1	-2.1	0

UBE2L6	UBE2L6_tv2	-2.1	0
RPS3A	RPS3A	-2.1	0
DPM1	DPM1	-2.1	0
C3orf38	C3orf38	-2.1	0
PPT1	PPT1	-2.1	0
ZNF569	ZNF569_tv1	-2.1	0
ZNF410	ZNF410_tv2	-2.1	0
ZNF33BL	ZNF33BL	-2.1	0.02
ARMT1	ARMT1_tv1	-2.1	0
PIGF	PIGF	-2.1	0.01
GPBP1	GPBP1_tv1	-2.1	0
BLOC1S6	BLOC1S6	-2.1	0
FCGR1A	FCGR1A	-2.1	0
CST15	ENSSSCG00000007123	-2.1	0.03
SLC25A40	SLC25A40	-2.1	0
NAE1	NAE1	-2.1	0
ITM2B	ITM2B	-2.1	0
RPL10	RPL10_tv1	-2.1	0
WDR61	WDR61_tv2	-2.1	0
COX7C	COX7C	-2.1	0
STK26	STK26_tv1	-2.1	0.01
RPS4XPs*	ENSSSCG00000022191	-2.1	0
EEF1B2	EEF1B2	-2.1	0
CCT4	CCT4	-2.1	0
NDUFAF4	NDUFAF4	-2.1	0.02
RPL38	RPL38	-2.1	0
NDUFA12	NDUFA12	-2.1	0
ESCO2	ENSSSCG00000009670	-2.1	0.03
PTTG1	ENSSSCG00000017032	-2.1	0
SLC30A6	SLC30A6	-2.1	0
UBA6	UBA6	-2.1	0
MTERF1	MTERF1	-2.1	0
LOC100155572	ENSSSCG00000002045	-2.1	0
GSTM3	GSTM3_tv1	-2.1	0.04
MOGAT2	MOGAT2	-2.1	0.04
TOMM22	ENSSSCG00000000097	-2.1	0
ZNF649	ZNF649	-2.1	0.04
TEFM	TEFM	-2.1	0
LGI2	LGI2	-2.1	0.01
FXYD2	ENSSSCG00000015084	-2.1	0.03
ARSB	ARSB	-2.1	0.01
TMSB10	TMSB10	-2.1	0.01
SLC35F4	SLC35F4	-2.1	0
CCL25	CCL25_tvX6	-2.1	0.04
OSGEPL1	OSGEPL1	-2.1	0
TATDN3	TATDN3	-2.1	0
HNMT	HNMT_tv1	-2.1	0

VPS26A	VPS26A_tv1	-2.1	0
LncRNA	LncRNA	-2.1	0.01
MBD4	MBD4	-2.1	0.02
PPP1CC	PPP1CC_tv1	-2.1	0
ZNF350L2*	ZNF350L2*	-2.1	0.02
DECR1	DECR1	-2.1	0
FAM227B	FAM227B	-2.1	0.02
TMEM14A	TMEM14A	-2.1	0
SNX4	SNX4	-2.1	0
ABHD11	ABHD11_tv1	-2.1	0.02
ANAPC13	ANAPC13	-2.1	0
KIAA1143	KIAA1143	-2.1	0.01
MRPS28	MRPS28	-2.1	0
SCOC	SCOC	-2.1	0
ELMOD2	ELMOD2_tv1	-2.1	0
THOC7	THOC7	-2.1	0
CETN3	CETN3	-2.1	0
HSPA13	HSPA13	-2.1	0
SLC35A1	SLC35A1	-2.1	0
KBTBD8	KBTBD8	-2.1	0.01
BCAS4	BCAS4_tv2	-2.1	0.05
CCT6A	CCT6A_tv1	-2.1	0
RNF170	RNF170_tv2	-2.1	0
RPL35	RPL35	-2.1	0
EIF2S3	EIF2S3	-2.1	0
UCHL3	UCHL3	-2.1	0
TRAPPC5	TRAPPC5	-2.1	0
ENSSSCG00000046793	ENSSSCG00000046793	-2.1	0.01
EIF3E	EIF3E	-2.1	0
IRAK1BP1	IRAK1BP1	-2.1	0
LIAS	LIAS	-2.1	0
CST9L	CST9L	-2.1	0.03
TNNT1	TNNT1	-2.1	0.01
TMEM70	TMEM70	-2.1	0
ATP6V1G1	ATP6V1G1	-2.1	0
TMEM128	TMEM128	-2.1	0
MRPL22	MRPL22	-2.1	0
ARMC1	ARMC1	-2.1	0
ZNF134	ZNF134_tv1	-2.1	0
YPEL5	YPEL5	-2.1	0
TMEM256	ENSSSCG00000037071	-2.1	0.01
MAGOHB	MAGOHB	-2.1	0
HINT1	HINT1	-2.1	0
ZNF711	ZNF711	-2.1	0
RPS27A	RPS27A	-2.1	0
CCDC191	CCDC191	-2.1	0
AIMP1	AIMP1_tv3	-2.1	0.05

FYTTD1	FYTTD1	-2.1	0
CCT2	CCT2_tv1	-2.1	0
SNRPB2	ENSSSCG00000037602	-2.1	0
NDUFB1	NDUFB1	-2.1	0
CTNNB1	CTNNB1_tv1	-2.1	0.02
PKIA	PKIA	-2.1	0
LncRNA	LncRNA	-2.1	0
UBE2L3	UBE2L3_tv1	-2.1	0
RPS13	RPS13	-2.2	0
KRT10	KRT10	-2.2	0
GLRX2	GLRX2_tv2	-2.2	0
CCDC58	CCDC58	-2.2	0
FKBP3	FKBP3_tv1	-2.2	0
DDX46	DDX46	-2.2	0.02
CROT	CROT_tv2	-2.2	0
CD24	CD24	-2.2	0
CSRNP3	CSRNP3_tv1	-2.2	0
NDUFB9	NDUFB9	-2.2	0
NUP54	NUP54	-2.2	0
COMMD8	COMMD8	-2.2	0
PSMA6	PSMA6_tv2	-2.2	0
HGF	HGF_tv1	-2.2	0
CREBRF	CREBRF_tv1	-2.2	0
SMNDC1	SMNDC1	-2.2	0
RAD51C	RAD51C_tv1	-2.2	0
WDR89	WDR89	-2.2	0
CMBL	CMBL	-2.2	0
ADH5	ADH5	-2.2	0
GPR34	GPR34_tv1	-2.2	0.04
POP5	POP5	-2.2	0
RPL5	RPL5	-2.2	0
PPA1	PPA1_tv1	-2.2	0
CHEK1	CHEK1_tv1	-2.2	0
RGS1	RGS1	-2.2	0.02
CDKN3	CDKN3_tv1	-2.2	0
NUDT2	NUDT2	-2.2	0
SH3BGRL2	SH3BGRL2	-2.2	0
ZNF709	ZNF709	-2.2	0
RBBP4	RBBP4	-2.2	0
NSUN3	NSUN3	-2.2	0
TMEM258	ENSSSCG00000027538	-2.2	0
ADAM10	ADAM10	-2.2	0
UNK156*	ENSSSCG00000048074	-2.2	0
ACYP2	ACYP2	-2.2	0
PRTFDC1	PRTFDC1_tv1	-2.2	0
VPS29	VPS29	-2.2	0
SNRPE	SNRPE	-2.2	0

ENSSSCG00000051580	ENSSSCG00000051580	-2.2	0.04
RTRAF	RTRAF	-2.2	0
TXNIP	TXNIP_tv1	-2.2	0
DUT	DUT	-2.2	0
ELOF1	ENSSSCG00000013613	-2.2	0.02
CMSS1	CMSS1_tv1	-2.2	0
MRPL46	MRPL46	-2.2	0
LOC102165822	ENSSSCG00000036524	-2.2	0.01
GNG10	GNG10_tv1	-2.2	0
INO80C	INO80C	-2.2	0.01
RPL34	RPL34	-2.2	0
GRIK1	GRIK1	-2.2	0.01
MZT1	MZT1	-2.2	0
RPS25	ENSSSCG00000036296	-2.2	0
TRIM33	TRIM33_tv	-2.2	0
RPL10A	ENSSSCG00000011206	-2.2	0.02
ARL5B	ARL5B	-2.2	0
PDCD1LG2	PDCD1LG2	-2.2	0.01
MRPS23	MRPS23	-2.2	0
RPL36AL	RPL36AL_tv	-2.2	0
GPN3	GPN3	-2.2	0
"MARCH7"	MARCH7_tvX1	-2.2	0
KLHL7	KLHL7_tv1	-2.2	0
RBBP8	RBBP8_tv1	-2.2	0.02
CKS2	CKS2	-2.2	0
SUCLG1	SUCLG1	-2.2	0
EBAG9	EBAG9_tv2	-2.2	0.03
COQ10B	COQ10B	-2.2	0
ATP5MC3	ATP5MC3	-2.2	0
AP3S1	AP3S1_tv	-2.2	0
CBX3	CBX3_tv1	-2.2	0.02
CASP6	CASP6_tva	-2.2	0
GTPBP10	GTPBP10	-2.2	0
ACTR6	ACTR6	-2.2	0
TMPO-AS1	TMPO-AS1	-2.2	0.05
WDR76	WDR76	-2.2	0
PRDX3	PRDX3	-2.2	0
NDUFS4	NDUFS4	-2.2	0
MAT2B	MAT2B	-2.2	0
SLC25A51	SLC25A51	-2.2	0
ENSSSCG00000035571	ENSSSCG00000035571	-2.2	0.04
TIMM9	TIMM9_tv1	-2.2	0
GGCT	ENSSSCG00000016679	-2.2	0
ARL6IP5	ARL6IP5	-2.2	0.02
ASB5	ASB5	-2.2	0.03
ENSSSCG00000049265	ENSSSCG00000049265	-2.2	0
MT-TP	ENSSSCG00000018096	-2.2	0

TANK	TANK_tv1	-2.2	0
ANO5	ANO5	-2.2	0
RAD17	RAD17	-2.2	0
EIF4A2	EIF4A2	-2.2	0
ELAVL2	ELAVL2	-2.2	0
ZNF32	ZNF32	-2.2	0
ARRDC3	ARRDC3_tv1	-2.2	0.01
NDUFA2	NDUFA2	-2.2	0
NAALAD2	NAALAD2	-2.2	0.01
IL13RA2	IL13RA2	-2.3	0.01
AZIN1	AZIN1	-2.3	0
ENSSSCG00000042997	ENSSSCG00000042997	-2.3	0
SMIM26	SMIM26	-2.3	0
TRIML2	TRIML2_tv2	-2.3	0.04
ZNF181	ZNF181_tv1	-2.3	0
ANKRD37	ANKRD37	-2.3	0.04
RBM7	ENSSSCG00000027905	-2.3	0
RRM2B	RRM2B_tv1	-2.3	0
TXNDC9	TXNDC9	-2.3	0
ZNF329	ZNF329	-2.3	0
SLITRK6	SLITRK6	-2.3	0.04
PDCD4	PDCD4	-2.3	0
LSM3	LSM3	-2.3	0
RPL12	RPL12	-2.3	0
ZBTB8OS	ZBTB8OS	-2.3	0
TRIM13	TRIM13	-2.3	0
ATP5PD	ATP5PD	-2.3	0
CCNG2	CCNG2	-2.3	0
ITPA	ITPA	-2.3	0
ZBTB6	ZBTB6	-2.3	0
EGFL6	EGFL6	-2.3	0.02
ATP5F1E	ENSSSCG00000007524	-2.3	0
ZBTB26	ZBTB26	-2.3	0
CLCN5	CLCN5	-2.3	0
GRB14	GRB14	-2.3	0
CC2D2B	CC2D2B_tvX1	-2.3	0.03
ARG1	ARG1_tv2	-2.3	0.01
POLM	ENSSSCG00000044483	-2.3	0
SENP8	SENP8	-2.3	0
COX5B	COX5B	-2.3	0
PLPP4	PLPP4	-2.3	0.02
RPL32	RPL32_tv1	-2.3	0
MYNN	MYNN	-2.3	0
ARAP2	ARAP2	-2.3	0
JADE3	JADE3	-2.3	0
ART3	ART3_tv1	-2.3	0.03
HEPACAM2	HEPACAM2	-2.3	0

LOC106510665	ENSSSCG00000051754	-2.3	0.01
GJA1	GJA1	-2.3	0
THOC5	THOC5	-2.3	0
HSPE1	HSPE1_tv1	-2.3	0
N4BP2L1	N4BP2L1_tv1	-2.3	0
DAZL	DAZL	-2.3	0.01
GTPBP8	GTPBP8_tv1	-2.3	0
HTRA4	HTRA4_tv1	-2.3	0.01
SKP1	SKP1	-2.3	0
SASS6	SASS6	-2.3	0
MCEE	MCEE	-2.3	0
BRK1	BRK1	-2.3	0
TMEM167B	TMEM167B	-2.3	0
ZFP14	ZFP14_tv1	-2.3	0
CDK1	CDK1_tv1	-2.3	0
C14orf119	C14orf119	-2.3	0
ATOX1	ATOX1	-2.3	0
ENSSSCG00000044841	ENSSSCG00000044841	-2.3	0
RPL27	RPL27	-2.3	0
DEPDC1	DEPDC1_tv1	-2.3	0
TYW5	TYW5	-2.3	0
UAP1	UAP1	-2.3	0
C9orf72	C9orf72	-2.3	0
IFRD2	IFRD2	-2.3	0
SRP9	SRP9	-2.3	0
ENSSSCG00000037067	ENSSSCG00000037067	-2.3	0.03
NDUFB5	NDUFB5	-2.3	0
CTRB2	CTRB2	-2.3	0
COQ3	COQ3_tv1	-2.3	0
HAPLN1	HAPLN1	-2.3	0
EMC3	EMC3	-2.4	0
FAM114A2	FAM114A2	-2.4	0
LSM8	LSM8	-2.4	0
PSMA5	PSMA5_tv1	-2.4	0
NAT1	NAT1	-2.4	0
NR0B1	NR0B1	-2.4	0
ZFP37	ENSSSCG00000005475	-2.4	0
UQCRC2	UQCRC2	-2.4	0
TNIP3	TNIP3_tv3	-2.4	0.05
RPS15	RPS15	-2.4	0
ITPRID1	ITPRID1	-2.4	0
RFC3	RFC3	-2.4	0
NXT2	NXT2_tv1	-2.4	0
SEC61G	SEC61G_tv1	-2.4	0
RPL9L*	ENSSSCG00000012427	-2.4	0
FAM96A	FAM96A_tv1	-2.4	0
ZC3H12B	ZC3H12B	-2.4	0.01

RPS27L	RPS27L	-2.4	0
RPL18Ps*	ENSSSCG00000031482	-2.4	0
DNAJC12	DNAJC12	-2.4	0.01
CRYZ	CRYZ	-2.4	0
HSD11B1L	HSD11B1L	-2.4	0
CYP39A1	CYP39A1_tv1	-2.4	0
ADCY1	ADCY1_tv1	-2.4	0.03
LINS1	LINS1	-2.4	0
MT-ND4L	MT-ND4L	-2.4	0
CENPN	CENPN_tv2	-2.4	0
ATP5MG	ATP5MG	-2.4	0
ZNF684L	ZNF684L	-2.4	0
DPYS	DPYS	-2.4	0.05
HAVCR2	HAVCR2	-2.4	0.01
NDUFC2	NDUFC2	-2.4	0
SUMO2	SUMO2	-2.4	0
RPL14	ENSSSCG00000011272	-2.4	0
RNF141	RNF141_tv1	-2.4	0
SUMO1	SUMO1	-2.4	0
DNAJC19	DNAJC19	-2.4	0
RPS18	RPS18	-2.4	0
IFIT1	IFIT1_tv1	-2.4	0
ADAM3B	ADAM3B	-2.4	0.03
LAMTOR2	LAMTOR2_tv1	-2.4	0
SMIM8	SMIM8	-2.4	0
RAB27B	RAB27B	-2.4	0
MOB4	MOB4_tv1	-2.4	0
ROMO1	ROMO1	-2.4	0
STMN1	STMN1	-2.4	0.02
KRR1	KRR1	-2.4	0
COPS2	COPS2	-2.4	0
MIS18A	MIS18A	-2.4	0
SELENOT	SELENOT	-2.4	0
ZNF658	ZNF658	-2.4	0
FNIP2	FNIP2	-2.4	0
RPS20	RPS20	-2.4	0
CCNB1IP1	CCNB1IP1	-2.4	0
LACTB2	LACTB2	-2.4	0
RPS11	RPS11	-2.4	0
RPL23A	RPL23A	-2.4	0
CCR2	CCR2	-2.4	0.03
MRO	MRO	-2.4	0
SGK3	SGK3_tv1	-2.4	0
THYN1	THYN1_tv1	-2.5	0.01
SAMD9_tv2	SAMD9_tv2	-2.5	0.01
Pseudogene	Pseudogene	-2.5	0
CCDC28A	CCDC28A	-2.5	0

C7H6orf125	C7H6orf125	-2.5	0
ERO1B	ERO1B	-2.5	0
INTU	INTU	-2.5	0
POMP	POMP	-2.5	0
NCK1	NCK1_tv	-2.5	0
COX7A2L	COX7A2L	-2.5	0
EIF2S3Y	EIF2S3Y	-2.5	0
RPS28	RPS28	-2.5	0
GEN1	GEN1	-2.5	0
H3F3B	H3F3B	-2.5	0
LOC100515582	ENSSSCG00000040019	-2.5	0.02
NDUFS5	NDUFS5	-2.5	0
FABP3	FABP3	-2.5	0
EFCAB10	EFCAB10_tv2	-2.5	0.01
STMN2	STMN2	-2.5	0.04
SNX16	SNX16	-2.5	0
ISCA2	ISCA2	-2.5	0
TMEM60	TMEM60	-2.5	0
BTG3	BTG3_tv2	-2.5	0
MRPS10	MRPS10	-2.5	0
AK6	AK6	-2.5	0.01
PET100	PET100	-2.5	0
ZNF300	ZNF300	-2.5	0
CCT6B	CCT6B	-2.5	0.03
C11orf71	C11orf71	-2.5	0.02
TRIM10	TRIM10	-2.5	0.04
PBK	PBK	-2.5	0
TUBE1	TUBE1	-2.5	0
CSKMT	CSKMT	-2.5	0.01
CHRNA6	CHRNA6_tv1	-2.5	0.03
ZFP82L*	ZFP82L*	-2.5	0
RPS18Ps2*	ENSSSCG00000046110	-2.5	0
CYP27B1	CYP27B1	-2.5	0
TIMM8A	TIMM8A	-2.5	0
RPS27	ENSSSCG00000031838	-2.5	0
SF3B6	SF3B6	-2.5	0
LncRNA	LncRNA	-2.6	0.03
SLC39A8	SLC39A8_tv2	-2.6	0
ENSSSCG00000034829	ENSSSCG00000034829	-2.6	0.02
ZNF501	ZNF501_tv1	-2.6	0.01
RPS7	RPS7	-2.6	0
UNK57*	UNK57	-2.6	0
MAGOH	MAGOH	-2.6	0
THAP5	THAP5	-2.6	0
PDCD10	PDCD10	-2.6	0
CNEP1R1	CNEP1R1_tv2	-2.6	0
LGALS1	LGALS1	-2.6	0

PLBD1	PLBD1	-2.6	0
RPL7	RPL7	-2.6	0
ERI2	ERI2_tv1	-2.6	0.01
PNO1	PNO1	-2.6	0
METTL18	METTL18	-2.6	0
RPS18Ps*	ENSSSCG00000008767	-2.6	0
MED21	MED21	-2.6	0
AOAH_tv1	AOAH_tv1	-2.6	0.02
LOC100621455	ENSSSCG00000041401	-2.6	0
NXNL2	NXNL2	-2.6	0.01
SLC6A15	SLC6A15	-2.6	0.01
RSL24D1	RSL24D1	-2.6	0
TSTD1	TSTD1	-2.6	0
MAEL	MAEL	-2.6	0.01
MRPL13	MRPL13	-2.6	0
TIAL1	TIAL1_tv2	-2.6	0.01
SLC25A14	SLC25A14_tvX3	-2.6	0
OXSM	OXSM_tv1	-2.6	0
ATP5PDL*	ENSSSCG00000016881	-2.6	0
CA7	CA7	-2.6	0.03
CST6	CST6	-2.7	0.01
LYRM2	ENSSSCG00000004324	-2.7	0
PTPN22	PTPN22	-2.7	0.01
RPL36A-HNRNPH2	RPL36A-HNRNPH2	-2.7	0
AKR1C1	AKR1C1	-2.7	0
RNASE12	RNASE12	-2.7	0
MBIP	MBIP	-2.7	0
MRPL50	MRPL50	-2.7	0
PCGF6	PCGF6_tv1	-2.7	0
CCNG1	CCNG1_tv	-2.7	0
LOC106509080	LOC106509080	-2.7	0.03
ZNF277	ZNF277	-2.7	0
LOC110257263	ENSSSCG00000041925	-2.7	0
TMEM210	TMEM210	-2.7	0.04
RPL39	ENSSSCG00000030849	-2.7	0
CDH3	CDH3	-2.7	0
LOC100627892	LOC100627892_tv1	-2.7	0
CAPZA2	CAPZA2	-2.7	0
C1orf146	C1orf146	-2.7	0.01
HIST1H4A	HIST1H4A	-2.7	0.01
RPS29	ENSSSCG00000038507	-2.7	0
BEX5	BEX5	-2.7	0
RPL17-C18orf32	RPL17-C18orf32	-2.7	0
FMO1	FMO1_tvX2	-2.7	0
UBB	UBB	-2.7	0
RPL22L1	ENSSSCG00000036114	-2.7	0
B2M	B2M	-2.7	0

MYL7	MYL7	-2.8	0
H2AFVPs*	ENSSSCG00000041469	-2.8	0.01
ZBBX	ZBBX	-2.8	0.05
LOC110262153	ENSSSCG00000047803	-2.8	0.01
FMC1	FMC1	-2.8	0
ATP5PO	ATP5PO	-2.8	0
LOC102159522	ENSSSCG00000041230	-2.8	0.01
RPL35A	RPL35A	-2.8	0
SAMD13	SAMD13	-2.8	0
ADAD1	ADAD1	-2.8	0
FAM200B	ENSSSCG00000041490	-2.8	0.02
SNAP23	SNAP23_tv2	-2.8	0.03
ARPP19Ps*	ENSSSCG00000037176	-2.8	0.02
ENSSSCG00000037551	ENSSSCG00000037551	-2.8	0.04
FOLH1	FOLH1_tv1	-2.8	0
CLECL1	CLECL1_tvX1	-2.8	0
MRPL39	MRPL39	-2.8	0
PSMA2	PSMA2	-2.8	0
RGS18	RGS18	-2.8	0
MORN2	MORN2	-2.8	0
FANCF	FANCF	-2.9	0.01
GTF2H5	GTF2H5	-2.9	0
PAGE2BL*	ENSSSCG00000049886	-2.9	0.02
ENSSSCG00000035654	ENSSSCG00000035654	-2.9	0
ENSSSCG00000042457	ENSSSCG00000042457	-2.9	0
CYB5R4	CYB5R4	-2.9	0
DDX25	DDX25	-2.9	0
LRRC39	LRRC39	-2.9	0
SLC44A5	SLC44A5_tv2	-2.9	0
AIF1	AIF1_tv3	-2.9	0
LIN9	LIN9_tv1	-2.9	0
BCL2A1	BCL2A1_tv1	-2.9	0.03
FASTKD2	FASTKD2_tv3	-2.9	0
KYNU	KYNU_tvX1	-2.9	0.02
IFI44L	IFI44L_tvX1	-2.9	0
C8orf37	C8orf37	-2.9	0
WFDC21L	WFDC21L	-2.9	0
PSMA8	PSMA8	-2.9	0.04
MT3	MT3	-2.9	0.01
LncRNA	LncRNA	-3	0
TSG101	ENSSSCG00000041063	-3	0.01
SMIM14	SMIM14	-3	0
ABHD18	ABHD18_tv4	-3	0.04
COX7B	COX7B	-3	0
CD36L1	CD36L1	-3	0
TMC2	TMC2	-3	0.04
ENSSSCG00000041281	ENSSSCG00000041281	-3	0

UBD	UBD	-3	0
MTRNR2L8	MTRNR2L8	-3	0
ZDBF2	ZDBF2	-3	0
WDR49	WDR49	-3	0.02
LY96	LY96	-3	0
ENSSSCG00000043633	ENSSSCG00000043633	-3	0.01
SETD9	SETD9	-3	0
SUMO4	SUMO4	-3	0
ABRACL	ABRACL	-3.1	0
COCH	COCH	-3.1	0
AP1S3_tv1	AP1S3_tv1	-3.1	0.04
RPS21	RPS21	-3.1	0
ETFRF1	ETFRF1	-3.1	0
SLIRP	SLIRP	-3.1	0
ATG4C	ATG4C_tv1	-3.1	0
GTPBP3	GTPBP3_tvIII	-3.1	0.03
PABPC5	PABPC5	-3.1	0
RPL27Ps*	ENSSSCG00000007500	-3.1	0
LOC110256941	ENSSSCG00000050742	-3.1	0.01
PRPS2	PRPS2_tv2	-3.1	0.02
RPS16	RPS16	-3.2	0
RPL14L*	ENSSSCG00000029003	-3.2	0
SRGN	SRGN_tv1	-3.2	0
DNAH8	DNAH8	-3.2	0.05
CALCB	ENSSSCG00000035649	-3.2	0.02
PCLAF	PCLAF_tv1	-3.2	0
ENSSSCG00000019832	ENSSSCG00000019832	-3.2	0.03
ZNF709L2*	ZNF709L2*	-3.2	0
FCGR3A	FCGR3A_tv3	-3.2	0
TFRC	TFRC_tv1	-3.2	0
MDP1	MDP1	-3.2	0.01
LY75	LY75	-3.2	0.03
RPS23L*	ENSSSCG00000012200	-3.2	0
FXN	FXN_tv1	-3.3	0.04
RPS15A	RPS15A	-3.3	0
LOC106508846	ENSSSCG00000043973	-3.3	0.04
SCGB2A2	SCGB2A2	-3.3	0.03
ENSSSCG00000042551	ENSSSCG00000042551	-3.3	0.05
RPL11L	RPL11L	-3.3	0
ARHGAP15	ARHGAP15_tv1	-3.3	0
RSAD2	RSAD2	-3.3	0.01
NDUFA1	NDUFA1	-3.3	0
H4C9	H4C9	-3.4	0
SLX4IP	SLX4IP	-3.4	0
GZMA	GZMA	-3.4	0.01
LOC100738624	ENSSSCG00000048214	-3.4	0.03
ZPBP2	ZPBP2	-3.4	0

KHDC1	ENSSSCG00000050380	-3.4	0.04
LOC110257033	ENSSSCG00000047522	-3.4	0.03
RPA3	RPA3	-3.4	0
RPS23Ps3*	ENSSSCG00000039020	-3.4	0
C8orf89	C8orf89	-3.5	0.02
ENSSSCG00000043724	ENSSSCG00000043724	-3.5	0.02
RPL22L1Ps*	ENSSSCG00000015460	-3.5	0
SLC30A10	SLC30A10	-3.5	0.03
CXCL10	CXCL10	-3.5	0
IL20RB	IL20RB	-3.5	0
PAGE2B	PAGE2B	-3.5	0
GDAP1L1	GDAP1L1	-3.5	0.05
C4BPA	C4BPA_tv1	-3.6	0
TMSB15B	TMSB15B_tv2	-3.6	0
LOC110261477	LOC110261477	-3.6	0
LCMT1	LCMT1_tv1	-3.6	0.03
IL18	IL18_tv1	-3.6	0.01
ENSSSCG00000042764	ENSSSCG00000042764	-3.6	0.05
PON2	PON2_tv1	-3.6	0
GLRX	GLRX_tv1	-3.6	0
KCNMB3	KCNMB3	-3.6	0
IFI6	IFI6	-3.6	0
PLAC8	PLAC8_tv2	-3.7	0
ZNF345	ZNF345_tv1	-3.7	0
ARPC3	ARPC3	-3.7	0
ACSM4	ACSM4_tv1	-3.7	0.04
IL1R2	IL1R2_tv1	-3.8	0
FRYL	FRYL_tvX4	-3.8	0.04
UGT2A1	UGT2A1_tv1	-3.8	0
IFI27	ISG12(A)	-3.8	0
TMPRSS15	TMPRSS15	-3.8	0.03
MEP1B	MEP1B	-3.8	0.04
IGJ	IGJ_tv1	-3.8	0
CYP3A46	CYP3A46	-3.8	0.03
MFF	MFF_tv6	-3.9	0.02
C12orf50	C12orf50	-3.9	0.03
LOC106505711	ENSSSCG00000050456	-3.9	0
ENSSSCG00000043294	ENSSSCG00000043294	-4	0.03
CUBN	ENSSSCG00000036046	-4	0.05
EYS	EYS_tv1	-4	0
MGAT4C	MGAT4C	-4	0
SLC16A6	SLC16A6	-4	0
RPS24Ps*	ENSSSCG00000016840	-4	0
MROH5L*	ENSSSCG00000033737	-4	0.01
GPR174	GPR174	-4	0.03
SFR1	SFR1	-4	0
PRKACB	PRKACB_tv1	-4.1	0

ALK	ALK	-4.1	0
TMSB15A	ENSSSCG00000028695	-4.1	0
CBR4	CBR4_tv1	-4.2	0
TAGLN3	TAGLN3	-4.2	0
SLC1A1	SLC1A1	-4.2	0
LOC102165335	ENSSSCG00000014186	-4.2	0.03
NUGGC	NUGGC	-4.2	0.03
IGSF8	IGSF8	-4.2	0.04
ZFAND4	ZFAND4	-4.2	0.04
SV2B	SV2B	-4.2	0.01
RPS16Ps*	ENSSSCG00000005432	-4.3	0
ENSSSCG00000048222	ENSSSCG00000048222	-4.3	0.05
C3orf14	C3orf14	-4.3	0
ZNF616	ZNF616	-4.4	0.04
ANKRD22	ANKRD22	-4.4	0
CIDEA	CIDEA_tv1	-4.4	0
CTRB2L	CTRB2L	-4.4	0.03
DEFB123	DEFB123_tv1	-4.5	0
RPL23AL*	RPL23AL*	-4.5	0
SVBP	SVBP_tvX1	-4.5	0
SPESP1	SPESP1	-4.5	0.05
DYNLT2B	DYNLT2B_tv2	-4.6	0
RDH16	RDH16	-4.6	0
CA5B	CA5B	-4.6	0.03
DEFB122	DEFB122	-4.7	0
ADH1C	ADH1C	-4.7	0.03
SNX14	SNX14_tv1	-4.7	0.01
NT5C3	NT5C3	-4.7	0.03
GNAT3	GNAT3	-4.7	0.01
PNLIPRP2	PNLIPRP2	-4.8	0
MIR1244-1	MIR1244-1	-4.8	0
TGM5	TGM5	-4.8	0.01
FAAP24	FAAP24_tv1	-4.8	0.03
PJVK	PJVK	-4.8	0
SDR16C5	SDR16C5_tv2	-4.9	0.02
APOH	APOH	-4.9	0.01
FGL1	FGL1	-5	0
S100A12	S100A12	-5	0
RPS27APs*	ENSSSCG00000035890	-5	0
ABCD3	ABCD3_tv1	-5.2	0.01
Y_RNA_4	Y_RNA_4	-5.2	0
lncRNA	lncRNA	-5.5	0
CASP1	CASP1	-5.5	0
LOC102166448	ENSSSCG00000042624	-5.9	0.01
NR1I2	NR1I2	-5.9	0
LYZ	LYZ-1	-6	0
C5orf58	C5orf58	-6.2	0

CYP3A29	CYP3A29	-6.2	0
SNX31	SNX31	-6.3	0
TSACC	TSACC	-6.9	0
T	T_tvX1	-7.2	0.02
SAA3	SAA3_tv1	-8.1	0.01
CLPS	CLPS_tv1	-8.6	0
RBM18	RBM18_tv1	-9.5	0.01
LAGE3	ENSSSCG00000049540	-9.7	0.01
KCNV2	KCNV2	-11.5	0.03
RPH3A	RPH3A	-13.4	0
CDX4	CDX4	-14.6	0
GZMH	GZMH	-17.4	0
LOC102167708	ENSSSCG00000034022	-20.2	0
PHYHIPL	PHYHIPL	-27.6	0
Endonuclease/reverse transcriptase	Endonuclease/reverse transcriptase	-30.8	0
ENSSSCG00000047702	ENSSSCG00000047702	-31.7	0.04
Endonuclease/reverse transcriptase	ENSSSCG00000040992	-57.9	0
UNK95*	UNK95*	-80.6	0
