# Supplementary material for: Soy Formula Is Not Estrogenic and Does Not Result in Reproductive Toxicity in Male Piglets: Results from a Controlled Feeding Study
Source: Nutrients. 2022 Mar 7;14(5):1126. doi: 10.3390/nu14051126 (PMC8912539; doi:10.3390/nu14051126)
Supplement: Supplementary file 1 [file nutrients-14-01126-s001.zip › Supplemental table 3.pdf]

Reannotated Gene	Gene	Fold Change	FDR
SMAD6	SMAD6	7.6	0.01
PCDHGA4	PCDHGA4_tv1	7.5	0
LOC100514340	ENSSSCG00000032383	7.4	0.04
MT-TV	ENSSSCG00000018062	6.9	0
NRIP2	NRIP2	5.8	0.02
SPTA1	SPTA1	4.5	0.03
UGT1A1	ENSSSCG00000036274	4.2	0.01
GUSB	GUSB	4	0.02
TMEM37	TMEM37	3.9	0
RNF183	RNF183	3.9	0.04
LOC110260790	ENSSSCG00000049984	3.6	0.04
APLN	APLN	3.2	0.01
NIM1K	NIM1K	3.2	0
TNR	TNR	3.1	0.01
LINGO1	LINGO1	3	0.04
NTF3	NTF3	3	0.02
CARNS1	CARNS1	2.6	0.01
CDK18	CDK18_tv3	2.6	0.04
GRIN2C	GRIN2C	2.4	0.04
TP53INP2	TP53INP2_tv1	2.4	0
APLNR	APLNR	2.4	0.04
LDLR	LDLR_tv1	2.4	0.04
ASCC2	ASCC2	2.2	0.04
IRAK2	IRAK2	2.1	0.04
CTRB2	CTRB2	-2.1	0.05
DIPK2A	DIPK2A	-2.2	0.04
NUF2	NUF2	-2.2	0.04
PDK4	PDK4	-2.2	0.01
SLF1	SLF1	-2.2	0.03
TXLNB	TXLNB	-2.3	0.04
TXNIP	TXNIP_tv1	-2.3	0
CCNG2	CCNG2	-2.3	0.03
PLBD1	PLBD1	-2.5	0
FAM72A	ENSSSCG00000039370	-2.5	0.02
TNNT1	TNNT1	-2.5	0.04
PPM1K	PPM1K	-2.6	0
GRB14	GRB14	-2.6	0.02
ALK	ALK	-2.7	0.01
VSIG1	VSIG1_tv2	-2.7	0
ARRDC3	ARRDC3_tv1	-2.8	0
RPS24Ps*	ENSSSCG00000016840	-2.8	0.03
RPS23L*	ENSSSCG00000012200	-2.8	0.01
IGJ	IGJ_tv1	-2.9	0.05
MGAT4C	MGAT4C	-2.9	0.01
LOC110261477	LOC110261477	-2.9	0.02
SELENOP	SELENOP	-3	0

TDH	ENSSSCG00000021767	-3.1	0
SPP1	SPP1_tv1	-3.3	0
RGS18	RGS18	-3.4	0.01
LRRC39	LRRC39	-3.4	0
COCH	COCH	-3.6	0
FCGR3A	FCGR3A_tv3	-3.6	0.03
ENSSSCG00000035654	ENSSSCG00000035654	-3.6	0
IFI27	ISG12(A)	-3.9	0
BEX2	ENSSSCG00000039273	-3.9	0
LY96	LY96	-4.3	0
FMO1	FMO1_tvX2	-4.8	0
SNX31	SNX31	-4.8	0.04
ENSSSCG00000042146	ENSSSCG00000042146	-5	0.03
IFI6	IFI6	-5.1	0
lncRNA	lncRNA	-5.2	0.01
GZMH	GZMH	-6.2	0.05
LOC106505425	ENSSSCG00000048436	-6.2	0.04
ANKRD22	ANKRD22	-6.3	0.03
ENSSSCG00000045049	ENSSSCG00000045049	-7.2	0.01
TMPRSS15	TMPRSS15	-7.5	0.04
KLK1	KLK1	-8	0.01
CYP3A29	CYP3A29	-8.2	0.05
CXCL13	CXCL13	-9.6	0
S100A12	S100A12	-13.3	0
UNK95*	UNK95*	-13.3	0.01
RNASE12	RNASE12	-23.7	0.01
Endonuclease/reverse transcriptase	Endonuclease/reverse transcriptase	-23.9	0
PHYHIPL	PHYHIPL	-40	0
ENSSSCG00000046140	ENSSSCG00000046140	-91.8	0
