# Supplementary material for: Soy Formula Is Not Estrogenic and Does Not Result in Reproductive Toxicity in Male Piglets: Results from a Controlled Feeding Study
Source: Nutrients. 2022 Mar 7;14(5):1126. doi: 10.3390/nu14051126 (PMC8912539; doi:10.3390/nu14051126)
Supplement: Supplementary file 1 [file nutrients-14-01126-s001.zip › Supplemental table 4.pdf]

Ingenuity Canonical Pathways	-log(p-value)
Mitochondrial Dysfunction	5.93
Oxidative Phosphorylation	4.97
Citrulline-Nitric Oxide Cycle	3.66
Arginine Biosynthesis IV	3.48
Urea Cycle	3.48
Superpathway of Citrulline Metabolism	2.65
Superpathway of Cholesterol Biosynthesis	2.11
Sirtuin Signaling Pathway	1.95
L-serine Degradation	1.85
Trans, trans-farnesyl Diphosphate Biosynthesis	1.73
Ferroptosis Signaling Pathway	1.68
GP6 Signaling Pathway	1.68
Tyrosine Degradation I	1.63
Ceramide Biosynthesis	1.55
Zymosterol Biosynthesis	1.55
Semaphorin Signaling in Neurons	1.5
Mineralocorticoid Biosynthesis	1.43
Estrogen Receptor Signaling	1.39
Glucocorticoid Biosynthesis	1.38
Glucocorticoid Receptor Signaling	1.36
